# Fantastic microbes and where to find them: evaluating learning-by-doing outcomes in a crowdfunded metagenomics workshop

**DOI:** 10.1093/femsle/fnag093

**Published:** 2026-08-17

**Authors:** Giulia Ghisleni, Ellen Dow, Teresa Iovino, Pamela Jael Colman-Vega, Alessia Dicesare, Emanuele Guanella, Yodit Maria Bacchi, Althea Colombo, Maristella Leccese, Matilde Marzucchi, Margherita Emma Gorla, Alessandra Caracciolo, Alessio Sala, Polina Makarycheva, Sharon Cristal Rubrica, Andrea Ferrier, Alice Armanni, Sara Fumagalli, Elisha Wood-Charlson, Antonia Bruno

**Affiliations:** Department of Biotechnology and Biosciences, University of Milano-Bicocca, 20126 Milan, Italy; Environmental Genomics and Systems Biology Division, E.O. Lawrence Berkeley National Laboratory, Berkeley, CA 94720, United States; Department of Biotechnology and Biosciences, University of Milano-Bicocca, 20126 Milan, Italy; Catalan Institute for Water Research (ICRA-CERCA), 17003 Girona, Spain; Department of Biotechnology and Biosciences, University of Milano-Bicocca, 20126 Milan, Italy; Department of Material Science and Nanotechnology, University of Milano-Bicocca, 20125 Milano, Italy; Department of Biotechnology and Biosciences, University of Milano-Bicocca, 20126 Milan, Italy; Department of Biotechnology and Biosciences, University of Milano-Bicocca, 20126 Milan, Italy; Department of Biotechnology and Biosciences, University of Milano-Bicocca, 20126 Milan, Italy; Department of Biotechnology and Biosciences, University of Milano-Bicocca, 20126 Milan, Italy; Dipartimento di Scienze della Terra e del Mare, University of Palermo, 90128 Palermo, Italy; Department of Life sciences, University of Siena, 53100 Siena, Italy; Department of Biotechnology and Biosciences, University of Milano-Bicocca, 20126 Milan, Italy; Department of Biotechnology and Biosciences, University of Milano-Bicocca, 20126 Milan, Italy; Department of Biotechnology and Biosciences, University of Milano-Bicocca, 20126 Milan, Italy; School of Biosciences and Veterinary Medicine, University of Camerino, 62032 Camerino, Italy; Department of Biotechnology and Biosciences, University of Milano-Bicocca, 20126 Milan, Italy; Department of Agricultural, Forest and Food Sciences, University of Turin, 10124 Turin, Italy; Department of Biotechnology and Biosciences, University of Milano-Bicocca, 20126 Milan, Italy; Department of Biotechnology and Biosciences, University of Milano-Bicocca, 20126 Milan, Italy; Environmental Genomics and Systems Biology Division, E.O. Lawrence Berkeley National Laboratory, Berkeley, CA 94720, United States; Department of Biotechnology and Biosciences, University of Milano-Bicocca, 20126 Milan, Italy

**Keywords:** microbiome education, metagenomics, participatory science, citizen science, crowdfunding, educational evaluation

## Abstract

Metagenomics offers a powerful framework for authentic, interdisciplinary learning, yet it remains underrepresented in undergraduate education due to technical and infrastructural barriers. We hypothesized that a research-based, learning-by-doing metagenomics workshop supported by accessible bioinformatics tools could enhance students’ perceived skills, self-efficacy, and conceptual understanding of metagenomic analysis. To test this hypothesis, we designed and evaluated a hybrid hands-on workshop in which undergraduate and postgraduate students analyzed real environmental shotgun metagenomic datasets generated from soil samples collected during a citizen science initiative. Using the graphical workflow platform KBase, participants completed an end-to-end metagenomic analysis, from quality control and assembly to genome reconstruction, taxonomic classification, functional annotation, and scientific presentation of results. Educational outcomes were assessed through validated retrospective pre-post questionnaires, self-efficacy scales, and an open-ended conceptual understanding task. Participants showed significant increases in perceived metagenomic skills and confidence in performing metagenomic analyses, while gains in perceived learning showed a positive trend. Conceptual understanding improved across educational levels, particularly among participants with limited prior experience. Together, these findings demonstrate that authentic, data-driven metagenomics activities can effectively lower barriers to computational biology and foster meaningful learning through hands-on research experiences.

## Teaser

A community-supported metagenomics workshop showed that learning through real research can make advanced microbiome science accessible to students with little prior experience, while building confidence, skills, and understanding.

## Introduction

Understanding and leveraging the microbiome requires the integration of biological, computational, and statistical expertise, as well as educational, societal, and interdisciplinary perspectives. Microbiome research sits at the intersection of biology, ecology, computational sciences, statistics, and technology, while also demanding skills in collaboration, communication, and scientific reasoning. As such, training in this field inherently involves a multidisciplinary approach that extends beyond traditional boundaries (Rosen and Hammrich 2020). At the same time, microbiome science presents unique challenges for both researchers and learners. The field is characterized by high levels of uncertainty, rapidly evolving methodologies, and complex data interpretation, which complicate both scientific practice and public understanding (Lorimer et al. 2019).

Despite its scientific and societal relevance, investigating microbiomes through metagenomic approaches remains underrepresented in formal educational curricula. Nearly two decades ago, Jurkowski et al. emphasized the urgency of integrating metagenomics into biology education (Jurkowski et al. 2007); however, a significant didactic transposition delay persists (Quessada and Clément 2007, Carvalho and Lima 2022). When included, metagenomics is typically confined to marginal components within broader courses (Rosen and Hammrich 2020), or to simplified classroom activities that expose students only to basic analytical steps without engaging them in authentic, end-to-end research workflows (Goller and Ott 2020).

Course-based Undergraduate Research Experiences (CUREs) have emerged as an effective framework by integrating research into teaching. These approaches allow students to participate in authentic scientific practice while simultaneously developing technical skills, critical thinking, and career-relevant competencies (Parks et al. 2020). Importantly, effective CURE designs emphasize accessibility for students with diverse backgrounds, the use of tools that lower technical barriers (e.g. minimizing the need for programming skills), and explicit engagement with the limitations and uncertainties of scientific knowledge (Goller and Ott 2020). Participation in authentic research experiences has also been associated with increased student persistence in STEM fields and higher levels of self-efficacy, particularly when activities involve active learning and hands-on engagement (Sanders and Hirsch 2014, Cottone and Yoon 2020). These benefits are especially relevant in computational biology, where perceived barriers to entry, such as programming requirements, can discourage participation.

Metagenomics is particularly well-suited for CURE-based implementation. Its inherently interdisciplinary nature and reliance on real-world data make it an ideal platform for experiential learning, enabling students to engage in discovery-based, technology-driven research (Sanders and Hirsch 2014). By engaging students in real data analysis, these approaches foster not only technical competencies but also an understanding of the epistemological dimensions of science, including uncertainty, reproducibility, and the complexity of interpreting biological systems (Alexander et al. 2024). Moreover, introducing students to emerging scientific fields has been shown to promote deeper engagement with fundamental scientific questions and to foster independent inquiry (Jurkowski et al. 2007). In this sense, learning becomes a process of knowledge co-production, where students actively construct understanding through interaction with data and scientific tools. In this context platforms, such as U.S. Department of Energy Systems Biology Knowledgebase (KBase) (Arkin et al. 2018, Wood-Charlson et al. 2026), Cyverse (Swetnam et al. 2024) and Galaxy (The Galaxy Community 2024) provide graphical user interfaces (GUI) and computational resources for building complex bioinformatics workflows, offer an opportunity to reduce technical barriers and broaden participation in metagenomics.

The number of educational initiatives integrating metagenomics into undergraduate and graduate curricula have grown in recent years. These include inquiry-based laboratory modules, modular CUREs, and stand-alone courses that combine wet-lab activities with bioinformatics analyses of authentic sequencing datasets, often using open-source, accessible platforms such as KBase, Galaxy, or other GUI-based tools (Kruchten 2020, Muth and Caplan 2020, Rosen and Hammrich 2020, Baker et al. 2021, Goller et al. 2021, Ho Pao et al. 2021, Morsink et al. 2025). Other initiatives have expanded metagenomics education through flexible teaching modules, case studies focused on research design and ethics, and online or blended training programs; demonstrating the adaptability of metagenomics education across diverse institutional contexts and learner populations (Duarte et al. 2020, Alexander et al. 2024, Whitham and Goller 2026).

Despite this strong alignment between metagenomics and CURE-based approaches, a critical gap remains: their implementation in educational settings remains limited. Faculty members have reported that successful CURE implementation requires both research experience and general proficiency in the relevant scientific domain to effectively guide their students’ projects. In addition, integrating complex laboratory techniques and equipment while allocating sufficient time and resources according to best practices within a course can be difficult (Shortlidge et al. 2016, Wang 2017). More broadly, time and financial constraints, together with the uncertainty of adapting research projects to the CURE format, have been identified as major barriers to implementation, particularly in life science courses (Govindan et al. 2020, Bell et al. 2025). Specific to metagenomics, barriers are not only logistical but also structural: limited access to sequencing technologies, computational infrastructure, and user-friendly analytical tools, as well as financial constraints, can prevent the adoption of such frameworks, particularly in resource-constrained institutions (Helmy et al. 2016, Işık et al. 2023). In addition, the perceived complexity of bioinformatics workflows and the need for programming expertise for both instructors and students may further discourage their integration into teaching (Al-Ageel et al. 2015, Drew et al. 2023). Moreover, metagenomics-based educational interventions are rarely accompanied by systematic, multi-dimensional evaluation.

As a result, it remains unclear how different components of learning, such as perceived skills, self-efficacy, and conceptual understanding, develop and interact in authentic, data-driven research environments.

**Figure 1:**
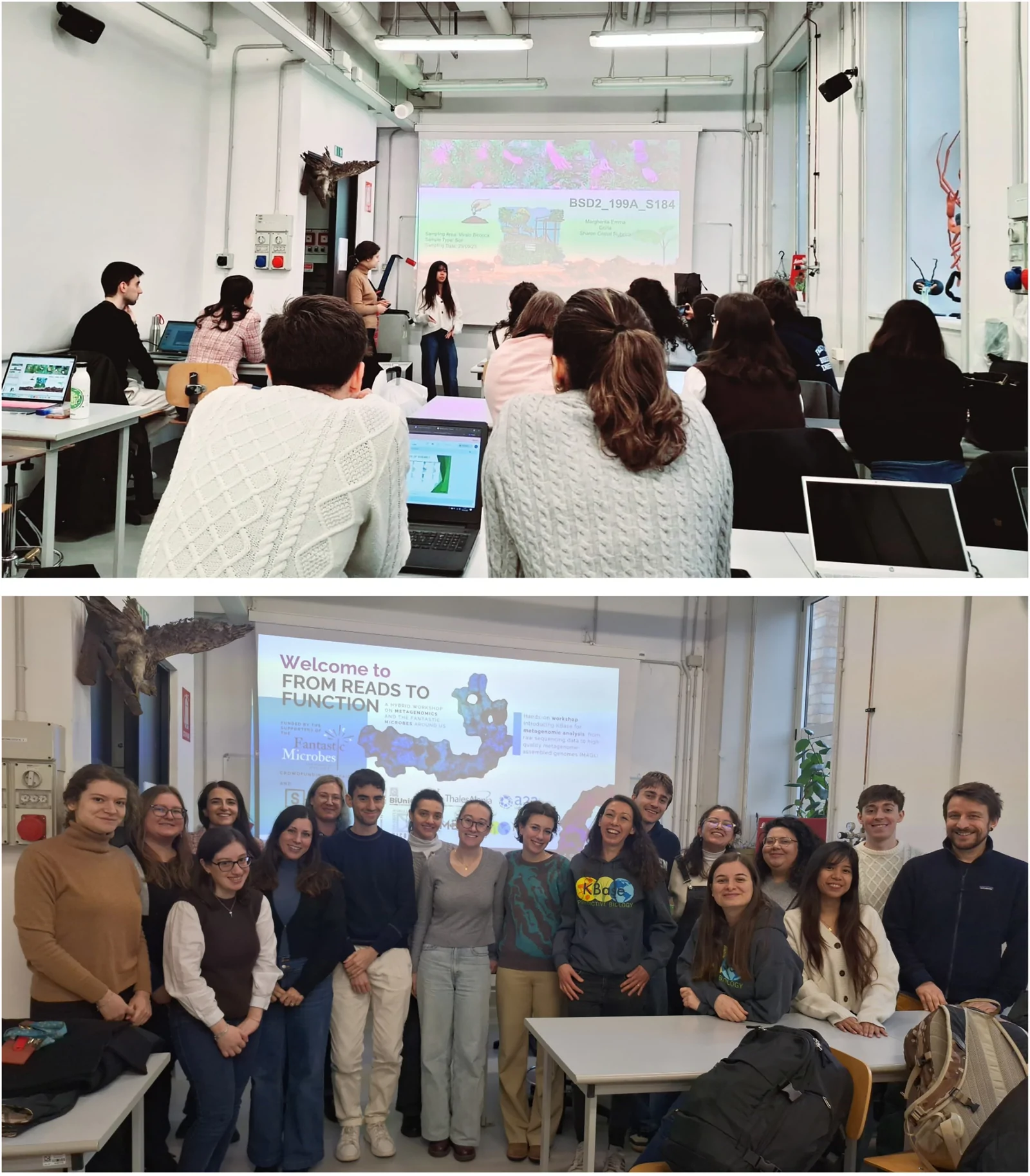
Settings and creative atmosphere during the workshop. First edition of the Workshop *From Reads to Function—Hybrid Workshop on Metagenomics and the Fantastic Microbes Around Us*. All participants provided signed consent for image release.

To address this gap, we designed and evaluated a hands-on, research-based workshop (Fig. 1, “From Reads to Function”) in which students performed metagenomic analyses on real environmental data collected by students (Ghisleni et al. 2025) through the KBase platform. The workshop was explicitly structured to provide an accessible yet authentic research experience, integrating computational tools with uncertainty, collaborative problem solving and inquiry-based learning. The objective of this study is to assess the educational impact of this intervention by examining changes in (i) perceived metagenomic skills, (ii) self-efficacy for learning and performing metagenomic analysis, and (iii) conceptual understanding of metagenomic workflows. Additionally, we investigated how these dimensions relate to each other, providing insight into the structure of learning gains in interdisciplinary, data-driven educational contexts.

## Materials and methods

### Sequencing and data generation

Six soil samples were collected from the open-air classroom, an outdoor educational setting within the plant nursery of the Vivaio Bicocca by students across three dates: 22 June 2023, 29 September 2023, and 27 March 2024, as part of the *Bicocca Sampling Days* (Ghisleni et al. 2025). DNA was extracted from 250 mg aliquots of each sample with the DNeasy PowerSoil Pro Kit (QIAGEN), and a final elution volume of 75 μL.

Shotgun metagenomic sequencing at 5Gbp per sample was provided through SeqCenter (Pittsburgh, PA). Illumina sequencing libraries were prepared using the tagmentation-based and PCR-based Illumina DNA Prep kit and custom IDT 10 bp unique dual indices (UDI) with a target insert size of 280 bp. No additional DNA fragmentation or size selection steps were performed. Illumina sequencing was performed on an Illumina NovaSeq X Plus sequencer in one or more multiplexed shared-flow-cell runs, producing 2 × 151 bp paired-end reads. Demultiplexing, quality control and adapter trimming was performed with Illumina bcl-convert1 (v4.2.4).

The metagenomic workflow was carried out using the free and open-source platform KBase (Arkin et al. 2018, Wood-Charlson et al. 2026) and followed an established metagenome-assembled genome analysis (Chivian et al. 2023) with default settings for each application, unless otherwise specified. FastQC v0.12.1 (Babraham Bioinformatics—FastQC A Quality Control tool for High Throughput Sequence Data 2023) assessed read quality, and PRINSEQ v0.20.4 (Schmieder and Edwards 2011) processed read trimming and quality control of the raw reads filtering out poly-G tails. Reads were assembled using MEGAHIT v1.2.9 with the meta-large setting (Li et al. 2015). Binning was performed using MaxBin2 v2.2.4 (Wu et al. 2014, 2016), MetaBAT2 v1.7 (Kang et al. 2015), and CONCOCT v1.1 (Alneberg et al. 2014). DasTOOL v1.1.2 (Camacho et al. 2009, Hyatt et al. 2010, Buchfink et al. 2015, Sieber et al. 2018) refined all bins for consensus. Resulting bins were filtered for quality at 50% completion rate with 10% contamination via CheckM v1.0.18 (Parks et al. 2015) and were annotated using RASTtk v1.073 (Aziz et al. 2008, Overbeek et al. 2014, Brettin et al. 2015), then ran through DRAM (Shaffer et al. 2020) and antiSMASH version 7.0 secondary metabolite prediction program (Blin et al. 2023, Medema et al. 2011)). Taxonomic identification was performed using the GTDB-Toolkit v2.3.2 (Chaumeil et al. 2022 ).

### Workshop design, outline, and contents

The ‘From Reads to Function’ workshop was designed to utilize the student-curated metagenomic data for students to go step-by-step through analysis with an overall goal to make connections between diversity and function of the metagenome-assembled genomes (MAGs) representing environmental microbiomes. Using KBase to introduce computational biology data analysis to undergraduates has been successfully implemented with the platform features of data sharing, reproducible workflows, and embedding documentation within analysis workflows-akin to laboratory notebooks (Dow et al. 2021). The platform uses a GUI, which removes barriers to participation, such as pre-requisite coding experience.

The hybrid workshop consisted of five parts to optimize time with students while running the various analysis tools in real time (Table 1). The first two sections were virtual—introducing students to the KBase platform (narrative.kbase.us), and running data analysis through the assembly stage. During the first virtual session, students were introduced to the Narrative, KBase workflow interface. Through a brief overview and hands-on introductory activity, students worked through the concepts behind data objects and analysis applications using GUI-wrapped tools, and editing/documenting steps with Markdown cells. In the second session, students were introduced to the data collected during the *Bicocca Sampling Days*. Data were pre-imported into template Narratives (https://kbase.us/n/257010/5/) that outlined the metagenomic analysis workflow steps. Within each Narrative, steps and available tools were introduced, alongside instructions for students to select the appropriate tool and run the analysis on their assigned data. This analysis framework was paired with lecture, the hands-on activity of walking through the analysis together, and prompts to answer at each stage. Students were organized into small, collaborative groups to analyze their sample. Students began with quality control and processed (trimmed and filtered) raw sequencing data, followed by read assembly. At this stage students ran read-based taxonomy tools to review later. They then proceeded to the analysis stage and prepared for the in-person workshop days.

**Table 1:** Overview of workshop contents, along with analysis steps and goals.

Part	Topic	Goals/steps
February 2 at 4pm Milan (7am PT)—onboarding 60–75 min		
1	Webinar—introduction to KBase ● What is KBase? ● Platform setup ● How to use data/apps	● Set up student accounts ● Add students to Org (data access)
February 3 at 4pm Milan (7am PT)–90 mins of workshop (time to run tools and trouble shoot)		
2	Webinar—metagenomics set-up ● Read quality control ● Process reads ● Read-based taxonomy ● Re-check processed reads ● Set up assembly run(s)	● Establish a standard for data quality; why sequences need to be trimmed/filtered after sequencing. ● Interpret read quality and review standards. ● Reasoning behind testing multiple assembly tools.
February 16 at 9am–5pm P.zza della Scienza, 2–Milan U3-BIOS Building Room 2010		
3	Workshop day 1 9:00–compare assemblies 9:45–set up binning tools 11:15–interpret taxonomy 12–13:00–lunch 13:00–interpret bin optimization results 13:45–assess bin quality 15:00–filter medium/high quality bins 16:00 set up functional annotation	● Understand and interpret relative quality of assemblies. ● Understand the concept of binning and the reasoning behind optimizing multiple tools. ● Compare read-based taxonomy to amplicon data. ● Understand bin optimization results. ● Define bin quality and cut-offs. ● Define metagenome-assembled genomes.
February 18 at 9am–5pm P.zza della Scienza, 2–Milan U3-BIOS Building Room 2010		
4	Workshop day 2 09:00–set up MAG taxonomy 09:30–interpret functional annotation 10:30–interpret taxonomy 11:15–build phylogenetic trees 12–13:00–lunch 13:00–synthesis 16:00–prepare presentations ● Review how to set up a scientific presentation ● Present results ● Asking questions	● Interpret DRAM (functional annotation) results. ● Understand estimation of taxonomic classification of bins. ● Interpret overall results. ● Develop conclusions and insights with context of data. ● Develop questions from results.
February 19 at 9am–12pm P.zza della Scienza, 2–Milan U3-BIOS Building Room 2010		
5	Workshop day 3–$\frac{1}{2}$ day 09:00–complete presentations, set-up 10:30–student Presentations	● Explain results and findings with contextual information to peers.

Over the first two in-person workshop days, students returned to their analysis workflows by comparing assembly quality. Students determined the relatively highest quality assemblies based on total length, longest read length, and N50/L50 values. With their chosen assemblies, students binned the contiguous sequences with several tools and a bin-optimizer. While binning tools ran, students revisited their reads-based taxonomy results. Once contigs were binned, students assessed bin quality based on completeness and contamination. Students set-up annotation tools on their medium- and high-quality bins (Bowers et al. 2017) to run over night. On the second workshop day, students ran their MAGs through taxonomic classification tools, analyzed the functional annotation outputs, and set up tools to build phylogenetic trees. The outputs of the taxonomic identity and phylogenetic placement were compared to the functional annotation for each MAG. To conclude their analysis, students synthesized results within the context of the environment where the samples were collected.

As part of their synthesis, students prepared their findings within short oral reports (Supplementary Data 1). On the final workshop day, students completed their reports on their sample’s MAGs and presented them to fellow workshop participants.

### Educational impact evaluation

The educational evaluation process followed the validated *Bicocca Sampling Day (BSD)* model (Ghisleni et al. 2025), adapted to metagenomics-related topics. Participants received information about the study objectives, anonymized data collection procedures, and data protection measures, in accordance with European data protection regulations (EU Regulation 2016/679, GDPR). The full original survey is provided in Supplementary Data 2. All statistical analyses were performed using R (version 4.5.1; script available in Supplementary Data 3). Given the small sample size (N = 14), both statistical significance tests and effect sizes were reported to ensure a more robust interpretation of the results. Applied tests, significance and effect sizes are reported in all related plots.

#### Skills for metagenomic inquiry

Participants’ perceived ability in conducting metagenomic analyses was assessed by adapting the full 12-item Skills for Science Inquiry retrospective customizable scale to the workshop context (Phillips et al. 2017) (reported internal consistency Cronbach’s α = 0.912). Following the recommended retrospective pretest–posttest approach, participants completed both a retrospective assessment of their pre-workshop skills and a current assessment of their post-workshop skills. All items were measured on a five-point Likert scale and scoring has been assessed accordingly (Strongly Disagree = 1, Disagree = 2, Neutral = 3, Agree = 4, Strongly Agree = 5).

#### Feeling of learning and doing metagenomic analysis with KBase

Participants’ perceived self-efficacy in learning and performing metagenomic analyses was assessed using the customizable Self-Efficacy for Learning and Doing Science (SELDS) scale (reported internal consistency Cronbach’s α = 0.92) (Porticella et al. 2017). The scale includes two subscales: Feeling of Learning (FoL) and Feeling of Doing (FoD), each consisting of four items. Both current and retrospective versions were constructed following the authors’ guidelines. All items were rated on a five-point Likert scale (Strongly Disagree = 1, Disagree = 2, Neutral = 3, Agree = 4, Strongly Agree = 5, with item 3 reverse-coded).

#### Understanding “metagenomic data analysis using KBase”

Following the BSD model, key elements required to define “metagenomic data analysis using KBase” were identified. A total of 20 core concepts were used to develop a scoring rubric.

A modified scoring system was applied (0 = missing, 1 = partially present, 2 = complete), resulting in a total score ranging from 0 to 40. Participants were asked to describe the metagenomic analysis workflow in their own words in their own words, and scoring was assessed according to the presence of the preestablished elements (Supplementary Data 4).

#### Descriptive qualitative feedback analysis

Open-ended participant comments collected at the end of the workshop were examined using descriptive thematic categorization. Responses were manually reviewed to identify recurring themes related to perceived usefulness, accessibility of the platform, confidence in metagenomic analysis, and overall workshop satisfaction. Representative quotations were selected to illustrate each theme.

## Results

### Participants demographics

The workshop was designed to host up to 20 participants, including Bachelor’s, Master’s, and PhD students. A total of 24 students from international institutions (Italy, Spain, and Hungary) initially expressed interest; however, 4 were unable to attend the in-person sessions and were excluded.

Fourteen participants completed the full workshop (online and in-person): one Bachelor’s student, seven Master’s students, and six PhD students. Among them, 28.6% reported no prior experience in bioinformatics, 50% reported basic experience (e.g. coursework), 14.3% intermediate experience (independent analysis), and only one participant reported advanced experience (regular analysis). All participants had a background in biology or biotechnology, and none had prior experience with KBase.

### Participatory metagenomics increases participants’ skills, feeling of learning and doing, and understanding

The educational evaluation survey was administered to all workshop participants as the last workshop activity. The anonymous scores of all participants are provided in Supplementary Data 4.

#### Workshop participation increases perceived skills

**Figure 2:**
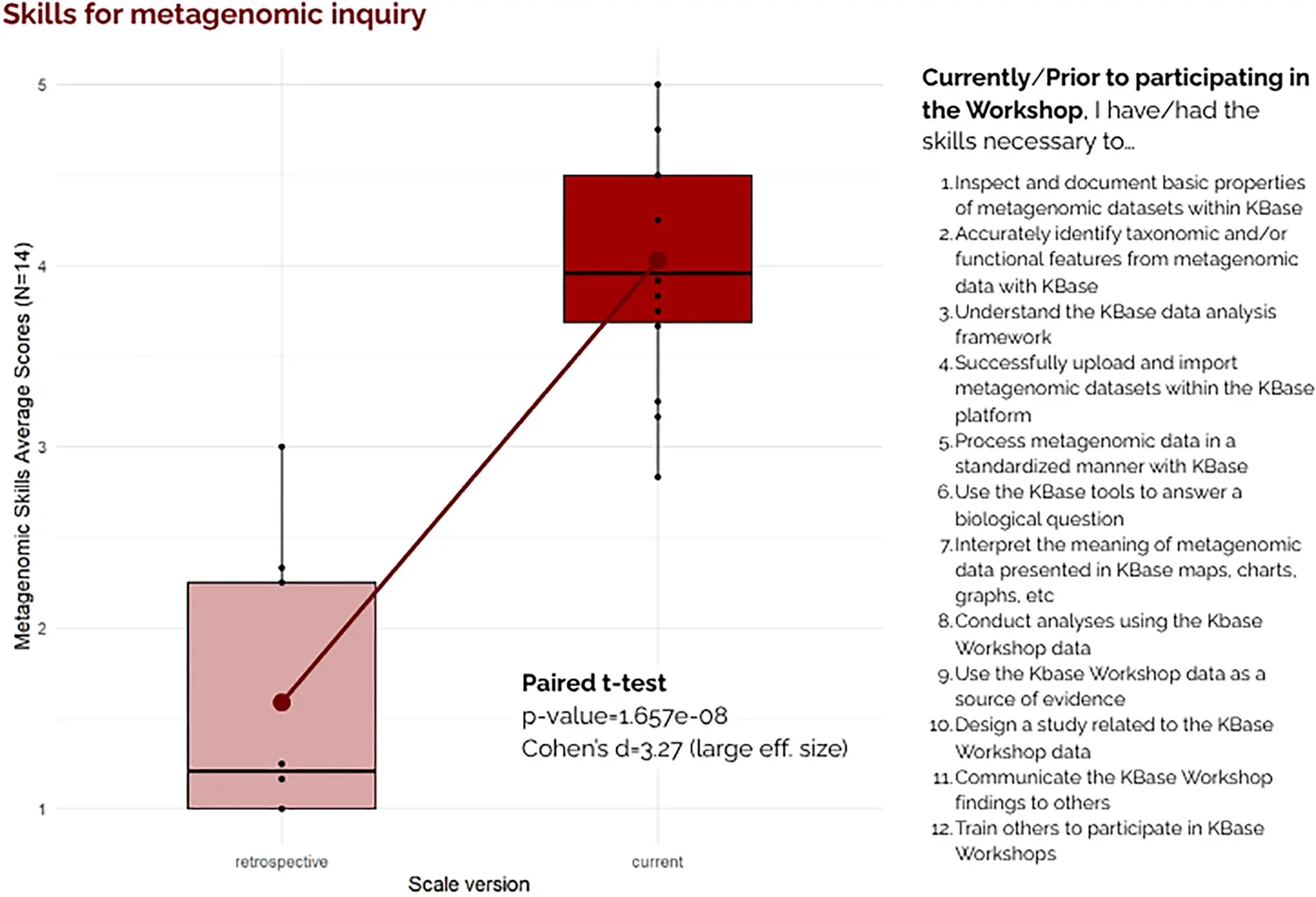
Skills for metagenomic inquiry. Retrospective and current individual average scores are shown as dots. Bigger dots represent the group mean, and the statistical test and significance used are reported. On the right, the items comprising the scale of 1 (Strongly Disagree) to 5 (Strongly Agree) are reported.

Participants’ perceived metagenomic skills were assessed using *current* and *retrospective* versions of the *Skills for metagenomic inquiry* scale. *Current* scores were significantly higher than *retrospective* scores (Wilcoxon signed-rank test, *P* = 0.00831, Strong Wilcoxon effect size r = 0.714), indicating a substantial increase in perceived skills following the workshop (Fig. 2). These results suggest the workshop effectively enhanced participants’ confidence to perform metagenomic analyses using KBase, including data exploration, MAG reconstruction, taxonomic and functional analysis, and interpretation and communication of results.

#### Workshop participation increases the feeling of doing more than learning

**Figure 3:**
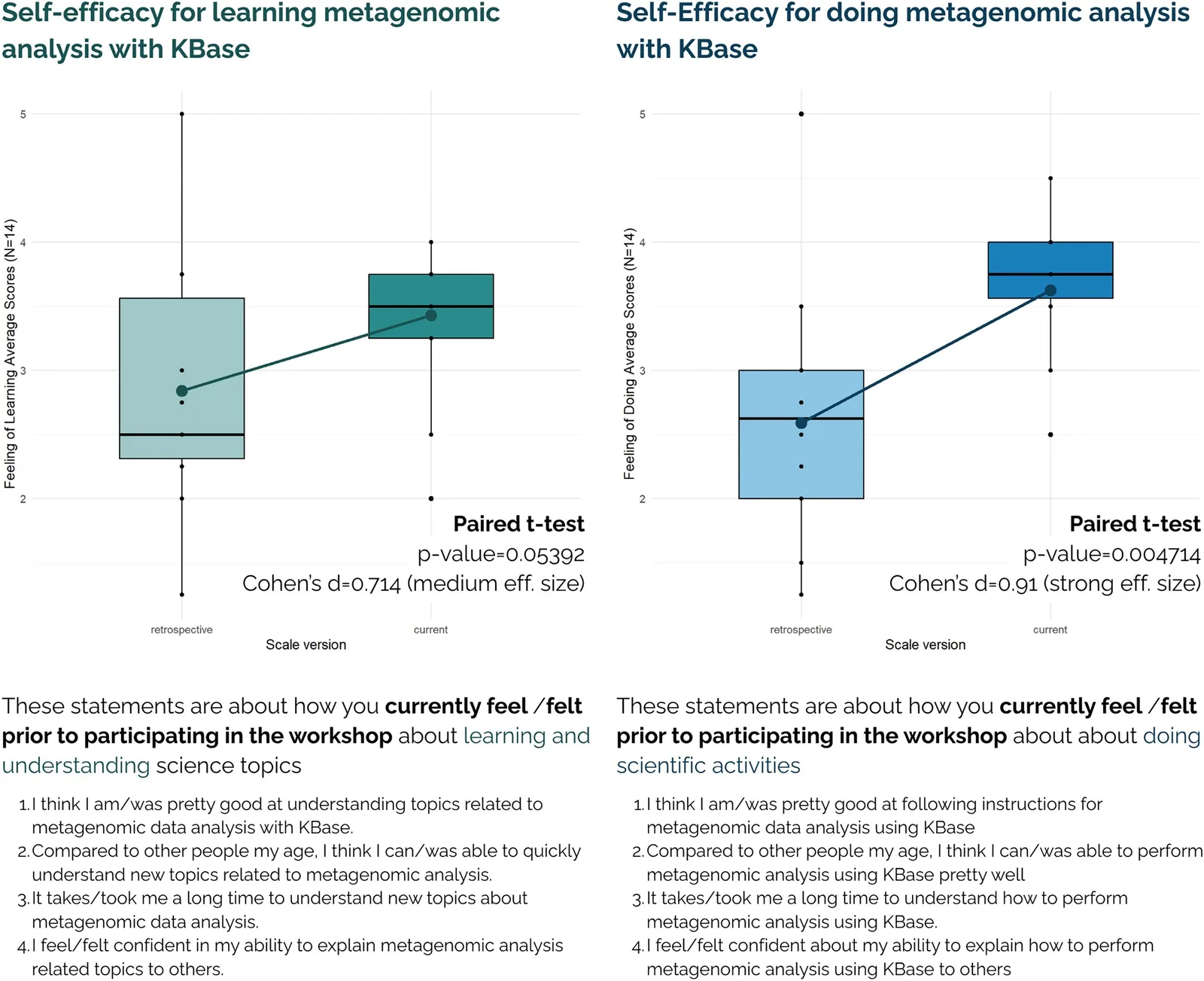
Self-efficacy for learning and doing metagenomic analysis with KBase. Retrospective and current individual average scores are shown as dots. Coloured dots represent the group mean and the statistical test and significance used are reported. Below each plot, the items comprising the respective scale of 1 (Strongly Disagree) to 5 (Strongly Agree) are reported. For item 3, the scale is reversed.

Self-efficacy for learning (FoL) and doing (FoD) metagenomics analysis were evaluated separately as previous work on participatory microbiome-related activities showed that students with scientific backgrounds benefit more from these activities on the practical side, and less on their perceived capability of learning scientific processes (Ghisleni et al. 2025). The increase in FoL showed a non-significant trend (Paired t-test, *P* = 0.05392, medium effect size Cohen’s d = 0.714), while FoD significantly increased after the workshop, indicating a strong effect (Paired t-test, *P* = 0.004714, medium effect size Cohen’s d = 0.91) (Fig. 3). These findings suggest hands-on, structured workshop activities particularly enhance participants’ confidence in performing metagenomic tasks, while improvements in perceived learning are present but less pronounced.

#### Workshop participation impacts on conceptual understanding

Participants’ understanding of “metagenomic data analysis using KBase” was assessed through an open-ended question. Due to the limited sample size, statistical comparisons between subgroups were not performed; however, descriptive patterns were examined.

**Figure 4:**
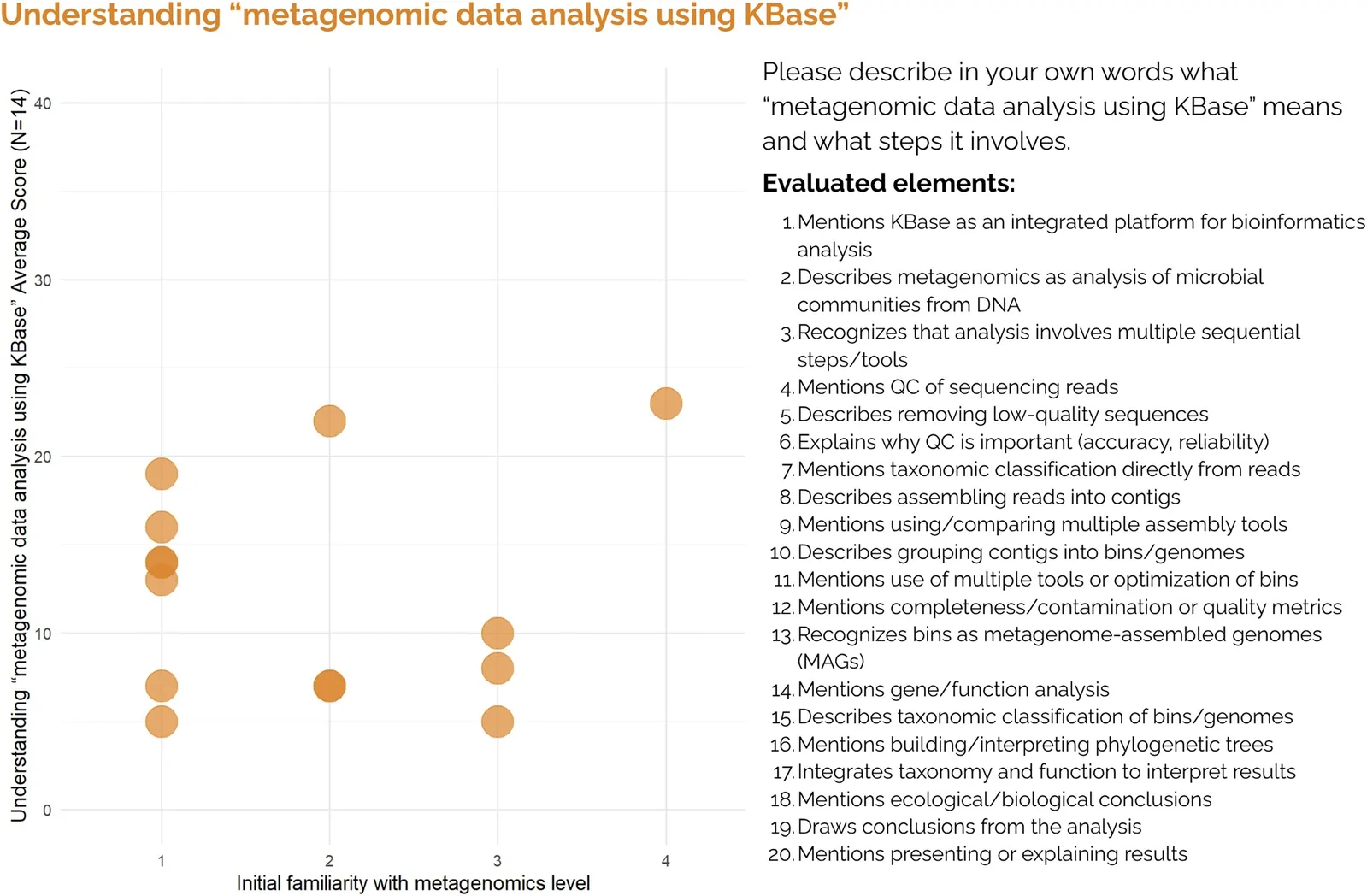
Understanding of “metagenomic data analysis using KBase”. Individual average scores are shown by initial levels of metagenomic analysis familiarity. On the right, the elements used for scoring are reported.

Understanding scores were interpreted in relation to participants’ initial familiarity with the topic and academic level (Fig. 4). Notably, participants with relatively high understanding scores (>10/40), have for the majority the lowest initial familiarity. This group included Bachelor’s, Master’s, and PhD students, suggesting that the workshop was effective in supporting conceptual understanding across different educational levels, particularly for participants with no prior experience.

#### Coherent structure of learning gains

**Figure 5:**
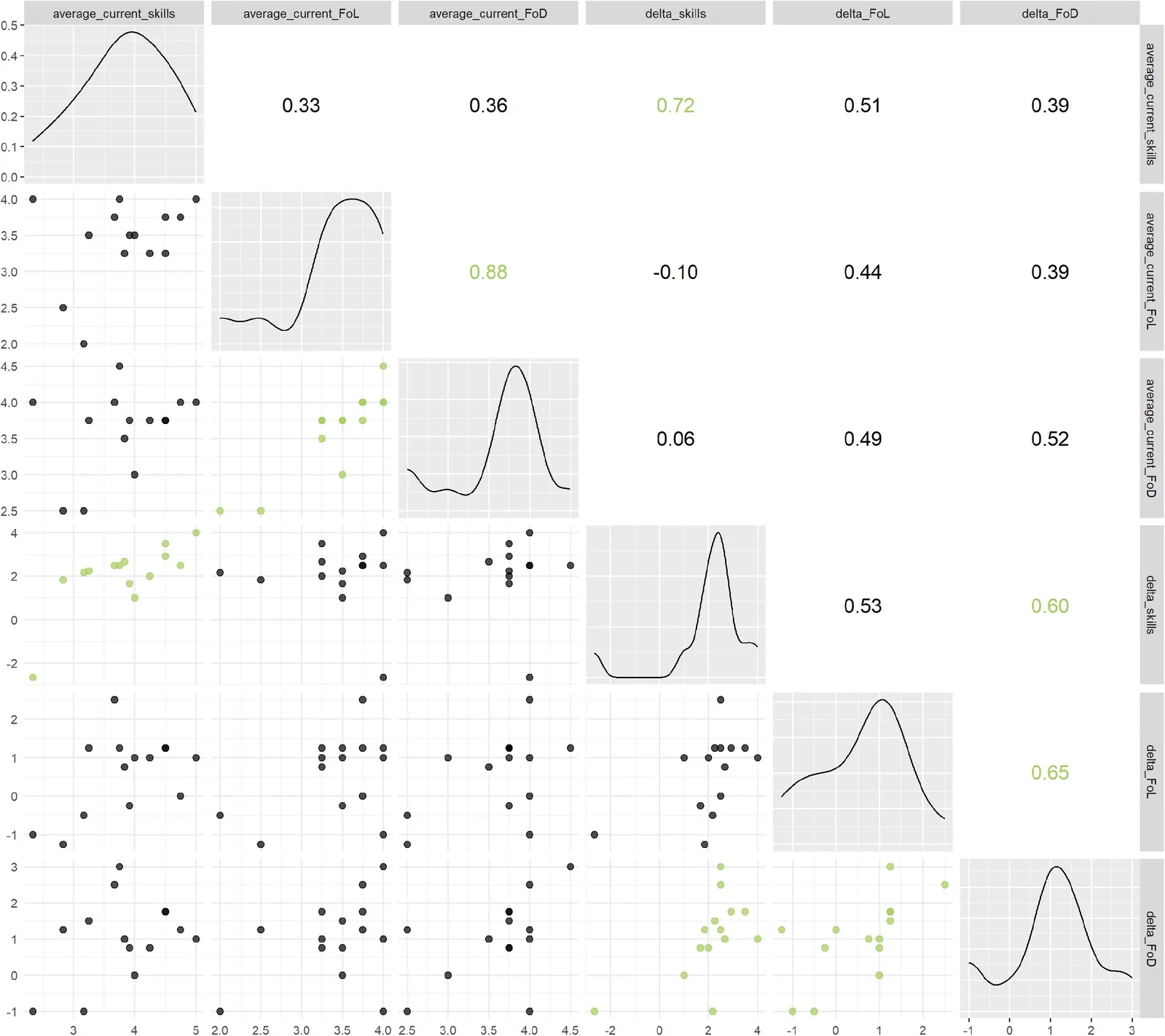
Correlation matrix of current scores and change (delta) measures. Pairwise relationships between variables are shown using scatterplots (lower panel), Pearson correlation coefficients (upper panel), and variable distributions (diagonal). Correlation coefficients with absolute values greater than 0.6 are highlighted in green. Current variables include perceived metagenomic skills, FoL, andFoD. Delta variables represent the change between post- and retrospective scores for each dimension.

Exploratory correlation analyses (Fig. 5) revealed a structured pattern of associations among post-intervention measures, while relationships between changes (delta scores) were more limited. At the post-intervention level, all constructs were positively associated. In particular, FoD and FoL showed a strong correlation (r = 0.88, *P* < 0.001), indicating that these dimensions are closely aligned in participants’ perceived learning experience. Perceived skills were moderately associated with both FoD (r = 0.59, *P* = 0.026) and FoL (r = 0.67, *P* = 0.008), suggesting that higher perceived competence tends to co-occur with stronger perceptions of both learning and doing. In contrast, analyses based on change scores (delta) provided weaker evidence for coordinated improvements across constructs. Changes in perceived skills were not significantly associated with changes in either FoD (r = 0.27, *P* = 0.35) or FoL (r = 0.26, *P* = 0.37). However, a significant positive association was observed between changes in FoD and FoL (r = 0.65, *P* = 0.013), indicating that individuals who reported greater increases in practical confidence also tended to report greater increases in perceived learning. Additionally, post-intervention skill levels were moderately associated with perceived gains in skills (r = 0.54, *P* = 0.045), suggesting that participants who reported higher final competence also tended to perceive larger improvements. Overall, these findings suggest that while post-intervention perceptions of skills, learning, and doing are coherently aligned, improvements across these domains may not fully co-vary, with the strongest coupling observed between the experiential dimensions of learning and doing.

#### Qualitative participant feedback supports quantitative findings

Open-ended participant feedback consistently reflected positive perceptions of the workshop experience and reinforced the quantitative evidence of educational benefit. Four recurrent themes emerged from participants’ comments: increased confidence in approaching metagenomic analysis, accessibility for beginners, perceived practical utility, and appreciation of the instructional support. Several participants described a substantial reduction in anxiety toward metagenomic analysis and bioinformatics tools. One participant reported being “completely lost” before the workshop but stated that participation made metagenomic analysis “feel way less scary,” highlighting the workshop’s role in lowering perceived entry barriers.

Accessibility was another recurring theme. Participants emphasized that the activities were clear and appropriately structured for beginners, with one student noting that “even without any prior knowledge in metagenome analysis I was able to understand all the subjects treated during the course.” This qualitative evidence aligns with the observed increases in perceived skills and self-efficacy.

Participants also highlighted the practical relevance of the workshop, reporting that the acquired knowledge and tools would support future independent analyses. As one respondent stated, the workshop provided “useful tools and knowledge that will allow me to perform data analysis in the future.” Finally, multiple comments emphasized the supportive learning environment and the value of direct interaction with instructors, suggesting that the participatory and guided structure of the workshop contributed substantially to the overall learning experience.

### Workshop’s scientific outputs

Six student groups went through the metagenomic analysis workflow using six datasets representing two replicates from the three BSD events (Ghisleni et al. 2026) (Table 2). While students performed their analysis, the data were co-assembled by combining the replicates within each BSD event (BSD1, BSD2, BSD4) to compare and improve MAG identification (https://kbase.us/n/245595/176/). Medium Quality (MQ) bins were filtered at >50% completeness and <10% contamination, with bins >90% completeness and <5% contamination considered as High Quality (HQ) (Bowers et al. 2017). Three individual samples yielded four distinct MAGs representing three bacterial classes. The co-assemblies yielded eight distinct MAGs representing six classes (one archaeal, five bacterial). On average, completion and contamination rates were 73.40% and 3.44%, respectively, with an average GC content of 60.13%. Each MAG had an average of 513 contigs and an average total length of 3.9 million base pairs.

**Table 2:** Medium and high (in bold) quality MAGs from assembled and co-assembled samples.

MAG	Taxonomy	Contigs	Comp. %	Cont. %	DRAM functional annotation
BSD1_189A_004	Xanthomonadaceae (f)	619	50.82	5.63	arsenate reductase (pt 1); respiratory nitrite reductase
BSD1_189B_002	*Microcoleus* sp.	363	98.03	0.44	arsenate reductase (pt 1); Photosystem I and II; alcohol dehydrogenase
BSD1_189B_003	Xanthomonadaceae (f)	509	87.21	2.44	acetate kinase; alcohol dehydrogenase
BSD1_189A-B_002	Acidimicrobiales	683	59.79	5.56	arsenate reductase (pt 1)
BSD1_189A-B_003	Xanthomonadaceae	439	64.52	2.48	acetyl-CoA synthetase
BSD1_189A-B_005	*Microcoleus* sp.	668	95.37	4.49	High affinity IV ETC complexes; Photosystem I and II; arsenate reductase (pt 1)
BSD1_189A-B_006	*Pseudoxanthomonas*	243	95.94	6.82	arsenate reductase (pt 1); acetyl-CoA synthetase
BSD1_189A-B_007	*Microlunatus* sp.	680	57.09	2.33	nitrite oxoreductase
BSD1_189A-B_009	*Xanthomonas prunicola*	308	67.18	2.29	arsenate reductase (pt 1)
BSD4_211C_002	Pyrinomonadaceae	621	91.31	2.61	arsenate reductase (pt 1); acetate to methane (pts 1,2,3)
BSD4_211B-C_001	Pyrinomonadaceae	717	91.45	1.76	arsenate reductase (pt 1); acetate to methane (pts 1,2,3)
BSD4_211B-C_003	Nitrososphaeraceae	366	55.86	7.77	respiratory nitrite reductase

Most of the predicted MAGs have many operons identified to have a range of low or no similarity to known secondary metabolites. There are 3 MAGs with 28–34 identified secondary metabolites. Notably, from the DRAM outputs, 5 MAGs from BSD1 samples contain arsenate reductase pt 1 (Xanthomonadaceae, Acidiimicrobia, *Microcoleus* sp. Pseudoxanthomonas, *Xanthomonas prunicola*). *Microcoleus* sp. MAG also contains both Photosystem subunits. From BSD4, Pyrinomonadaceae contains all three subunits of acetate to methane metabolism.

## Discussion

This study evaluated the educational impact of a hands-on, research-based metagenomics workshop and explored how different dimensions of learning (perceived skills, FoL, and FoD) develop and relate to each other in an authentic data-driven context. Our findings indicate that participatory metagenomics activities can effectively enhance students’ perceived skills and self-efficacy, while also revealing a structured but not fully synchronous pattern of learning gains across dimensions.

Engaging with a complete, end-to-end metagenomic workflow, from raw data processing to interpretation, supported the development of perceived operational competence, as reflected by the significant increase in perceived metagenomic skills following the workshop, with a strong effect size. Unlike simplified classroom activities, the use of real datasets and authentic analytical steps likely contributed to this outcome by exposing participants to the complexity and structure of actual research practices. In this sense, the workshop aligns with the principles of authentic research experiences, in which engagement with real scientific problems supports meaningful learning (Weng et al. 2022). An additional strength of the workshop was the possibility of engaging students with datasets derived from samples some of them had previously collected during the Bicocca Sampling Days initiative. This continuity between sample collection, data analysis, and interpretation may have reinforced participants’ sense of ownership and engagement with the scientific process, supporting the development of a more authentic research experience. Previous work in citizen science and research-based education suggests that involving participants across multiple stages of scientific inquiry can strengthen both learning and identification with scientific practices (Bonney et al. 2009, 2016).

Students were able to understand the impacts of sequencing depth on completeness of assembling MAGs from soil samples. Through using KBase, students explored the potential function of their identified MAGs for both completeness and presence. For example, one student group identified a highly complete Microcoleus MAG containing genes associated with Photosystems I and II, suggesting a potential photosynthetic lifestyle, while other groups detected functions related to carbon and energy metabolism, including alcohol dehydrogenases, acetate kinases, and acetyl-CoA synthetases. These observations encouraged students to move beyond workflow execution and engage in biological interpretation of metagenomic results. In addition, one surprising but interesting finding was identifying an arsenate reductase subunit across several MAGs. Students were able to contextualize this finding and connect the potential presence of metabolizing arsenic with soil samples collected from a previously industrialized location in the Bicocca District of Milan. Notably, the co-occurrence of both arsC (grx) and arsC (trx) variants, two convergently evolved, biochemically distinct arsenate reductase families, within MAGs from the same sample (e.g. BSD1_189A) suggested a redundant detoxification strategy consistent with sustained arsenic selective pressure, a pattern previously associated with industrially impacted soils (Ben Fekih et al. 2018, Dunivin et al. 2019, Rueangmongkolrat et al. 2024). These observations suggest that the workshop supported not only technical skill development but also the ability to contextualize and critically interpret metagenomic data.

Despite the small sample size (complemented with effect size estimation and graphical data visualization to strengthen the robustness of the analysis), the educational evaluation showed a differentiated pattern when considering self-efficacy dimensions. While both FoL and FoD increased, only FoD showed a statistically significant improvement, accompanied by a strong effect size. This suggests that hands-on engagement primarily strengthens participants’ confidence in their ability to perform tasks, while perceived learning may require longer or repeated exposure to consolidate. One possible interpretation is that practical activity provides an immediate and tangible sense of competence (“I can do this”) (Uzorka et al. 2024). Conversely, the perception of conceptual understanding may develop more gradually and be influenced by cumulative exposure to biological concepts across university training, potentially attenuating the observable short-term impact of the workshop on FoL. This distinction is particularly relevant in computational biology contexts, where initial barriers, such as unfamiliar tools or perceived technical difficulty, can be reduced through guided practice opposed to conceptual instruction alone.

The analysis of conceptual understanding further supports the effectiveness of the workshop, particularly for participants with limited prior experience. Descriptive patterns indicate that students with the lowest initial familiarity were able to achieve relatively higher understanding scores, regardless of academic level. This suggests that the workshop design, combining structured guidance, collaborative work, and accessible computational tools, may function as an equalizing mechanism, enabling participants with diverse backgrounds to engage meaningfully with complex topics.

Exploratory correlation analyses, that do not imply causal relationships between variables, provide preliminary insight into the structure of learning processes. At the post-intervention level, all constructs were positively associated, with a particularly strong relationship between FoD and FoL. This finding indicates that participants who feel more confident in performing metagenomic tasks also tend to perceive themselves as learning more effectively, suggesting that these experiential dimensions are closely aligned within the learning experience. Perceived skills showed moderate associations with both FoD and FoL, indicating that competence, learning, and doing are interconnected, though not interchangeable constructs.

In contrast, analyses based on change scores (delta) revealed a more nuanced pattern. Improvements in perceived skills were not significantly associated with changes in either FoD or FoL, suggesting that gains in these dimensions do not necessarily co-occur at the individual level. However, a significant positive association was observed between changes in FoD and FoL, indicating that increases in practical confidence tend to be accompanied by increases in perceived learning. Together, these findings suggest that while post-intervention perceptions form a coherent system, the processes underlying improvement may be partially decoupled. In particular, experiential engagement (doing) may represent a central mechanism linking learning and confidence, whereas perceived skill development may follow a more complex trajectory.

The observed positive association between post-intervention skill levels and perceived gains in skills further supports this interpretation. Participants who reported higher final competence also reported larger improvements, suggesting a relationship between outcome level and perceived progress. This may reflect increased metacognitive awareness, whereby individuals with greater competence are better able to recognize and evaluate their own development (Tuononen et al. 2023).

Qualitative feedback further supports the role of KBase in lowering perceived barriers to participation in computational biology and reinforces the quantitative findings. Recurring themes included increased confidence in approaching metagenomic analysis, perceived accessibility of the tools, and the practical relevance of the acquired skills. Several participants explicitly reported a reduction in initial anxiety and uncertainty, highlighting the role of the workshop in lowering perceived barriers to entry. These observations are consistent with the quantitative increase in FoD and support the interpretation that hands-on, guided experiences are particularly effective in fostering confidence in computationally intensive domains.

Some limitations should be considered. The use of self-reported measures might introduce the possibility of response bias, particularly in retrospective assessments. Previous studies have shown that students are generally able to accurately identify their own learning through retrospective self-assessment approaches, and that these methods may better exclude perceived changes in topics that were not actually taught (Bhanji et al. 2012). Therefore, while subjective by nature, the adopted measures remain appropriate for capturing participants’ perceived educational outcomes and self-efficacy changes. Nevertheless, this approach is widely adopted in citizen science and educational research contexts, where perceived skills and self-efficacy represent key outcomes of interest, particularly in the light of building confidence. Importantly, all scales used in this study are previously validated instruments, supporting the reliability and comparability of the measurements.

The design of the workshop likely contributed to the observed outcomes. The use of real-world data, combined with a structured yet flexible workflow, provided an authentic research context while maintaining accessibility. The adoption of a GUI platform reduced technical barriers associated with programming (Huang et al. 2025), allowing participants to focus on conceptual understanding and analytical reasoning. Additionally, collaborative group work and continuous instructor support may have contributed to a supportive learning environment, facilitating both engagement and persistence.

These findings are consistent with previous research on CUREs, which highlights the benefits of authentic, active learning in promoting self-efficacy and engagement (Goller and Ott 2020, Parks et al. 2020). However, this study extends existing literature by examining not only the magnitude of learning gains but also the relationships between different dimensions of learning. The combined use of retrospective pre-post measures and exploratory correlation analyses provides a more nuanced understanding of how skills, learning, and doing co-evolve in interdisciplinary, data-driven contexts.

The study provides relevant implications for both educational practice and research. First, it supports the use of metagenomics as an effective platform for integrating research and education, particularly when combined with accessible computational tools. Second, it highlights the importance of hands-on, experiential learning in fostering confidence and engagement, especially in computational disciplines. Third, it suggests that evaluating educational interventions through multiple dimensions, including their interrelationships, can provide deeper insight into learning processes than single-outcome measures.

Future research should further investigate the temporal dynamics of learning gains, the role of prior experience, and the extent to which perceived improvements align with objective skill development. Exploring different instructional designs and comparing workshop-based approaches with other educational models may further clarify how to optimize strategies for teaching complex, interdisciplinary topics.

In conclusion, this study demonstrates that participatory, research-based metagenomics workshops can effectively enhance perceived skills, confidence, and understanding, while revealing a structured yet complex pattern of learning development. These findings underscore the value of authentic, data-driven educational experiences in preparing students to engage with the challenges of modern computational biology.

## Supplementary Material

fnag093_Supplemental_Files

## References

[bib1] Babraham Bioinformatics—FastQC A quality control tool for high throughput sequence data. 2023. https://www.bioinformatics.babraham.ac.uk/projects/fastqc/ (5 May 2026, date last accessed).

[bib2] Al-Ageel N, Al-Wabil A, Badr G et al. Human factors in the design and evaluation of bioinformatics tools. Procedia Manuf. 2015;3:2003–10. 10.1016/j.promfg.2015.07.247

[bib3] Alexander CC, Gaudier-Diaz MM, Kleinschmit AJ et al. A case study to engage students in the research design and ethics of high-throughput metagenomics. J Microbiol Biol Educ. 2024;25:e00074–23. 10.1128/jmbe.00074-23 38661414 PMC11044643

[bib4] Alneberg J, Bjarnason BS, Bruijn Id et al. Binning metagenomic contigs by coverage and composition. Nat Methods. 2014;11:1144–6. 10.1038/nmeth.3103 25218180

[bib5] Arkin AP, Cottingham RW, Henry CS et al. KBase: the United States Department of Energy Systems Biology Knowledgebase. Nat Biotechnol. 2018;36:566–9. 10.1038/nbt.4163 29979655 PMC6870991

[bib6] Aziz RK, Bartels D, Best AA et al. The RAST server: rapid annotations using subsystems technology. Bmc Genomics [Electronic Resource]. 2008;9:75. 10.1186/1471-2164-9-75 18261238 PMC2265698

[bib7] Baker SS, Alhassan MS, Asenov KZ et al. Students in a course-based undergraduate research experience course discovered dramatic changes in the bacterial community composition between summer and winter lake samples. Front Microbiol. 2021;12:579325. 10.3389/fmicb.2021.579325 33679627 PMC7929996

[bib8] Bell J, Martinez-Vaz BM, Bell E. What Is a CURE? CUREs as High Impact Practices for all Students. Washington, D.C.: ACS Publications. 2025. 10.1021/bk-2025-1493.ch001 published online 31 Jan.

[bib9] Ben Fekih I, Zhang C, Li YP et al. Distribution of arsenic resistance genes in prokaryotes. Front Microbiol. 2018;9:2473. 10.3389/fmicb.2018.02473 30405552 PMC6205960

[bib10] Bhanji F, Gottesman R, Grave Wd et al. The retrospective pre–Post: a practical method to evaluate learning from an educational program. Acad Emerg Med. 2012;19:189–94. 10.1111/j.1553-2712.2011.01270.x 22320369

[bib11] Blin K, Shaw S, Augustijn HE et al. antiSMASH 7.0: new and improved predictions for detection, regulation, chemical structures and visualisation. Nucleic Acids Res. 2023;51:W46–W50. 10.1093/nar/gkad344 37140036 PMC10320115

[bib12] Bonney R, Ballard H, Jordan R et al. Public participation in scientific research: defining the field and assessing its potential for informal science education. A CAISE Inquiry Group report. In: Online Submission. CAISE, Washington DC, 2009. https://eric.ed.gov/?id=ED519688 (21 Mar. 2024, date last accessed).

[bib13] Bonney R, Phillips TB, Ballard HL et al. Can citizen science enhance public understanding of science?. Public Underst Sci. 2016;25:2–16. 10.1177/0963662515607406 26445860

[bib14] Bowers RM, Kyrpides NC, Stepanauskas R et al. Minimum information about a single amplified genome (MISAG) and a metagenome-assembled genome (MIMAG) of bacteria and archaea. Nat Biotechnol. 2017;35:725–31. 10.1038/nbt.3893 28787424 PMC6436528

[bib15] Brettin T, Davis JJ, Disz T et al. RASTtk: a modular and extensible implementation of the RAST algorithm for building custom annotation pipelines and annotating batches of genomes. Sci Rep. 2015;5:8365. 10.1038/srep08365 25666585 PMC4322359

[bib16] Buchfink B, Xie C, Huson DH. Fast and sensitive protein alignment using DIAMOND. Nat Methods. 2015;12:59–60. 10.1038/nmeth.3176. 25402007

[bib17] Camacho C, Coulouris G, Avagyan V et al. BLAST+: architecture and applications. BMC Bioinf. 2009;10:421. 10.1186/1471-2105-10-421. PMC2803857 20003500

[bib18] Carvalho GS, Lima N. Chapter 2–public perception of microorganisms and microbiology education: a need for enhancing society’s microbiology literacy. In: Kurtböke İ (ed.), Importance of Microbiology Teaching and Microbial Resource Management for Sustainable Futures. Academic Press, 2022, 31–45. 10.1016/B978-0-12-818272-7.00006-7.

[bib19] Chaumeil PA, Mussig AJ, Hugenholtz P et al. GTDB-Tk v2: memory friendly classification with the genome taxonomy database. Bioinformatics. 2022;38:5315–6. 10.1093/bioinformatics/btac672. 36218463 PMC9710552

[bib20] Chivian D, Jungbluth SP, Dehal PS et al. Metagenome-assembled genome extraction and analysis from microbiomes using KBase. Nat Protoc. 2023;18:208–38. 10.1038/s41596-022-00747-x. 36376589

[bib21] Cottone AM, Yoon S. Improving the design of undergraduate biology courses toward the goal of retention: the case of real-world inquiry and active learning through metagenomics. J Microbiol Biol Educ. 2020;21: 1–10. 10.1128/jmbe.v21i1.1965 PMC7148147 32313595

[bib22] Dow EG, Wood-Charlson EM, Biller SJ et al. Bioinformatic teaching resources—For educators, by educators—Using KBase, a free, user-friendly, open source platform. Front Educ. 2021;6:711535. 10.3389/feduc.2021.711535

[bib23] Drew J, Morgan W, Galindo S et al. Revisiting barriers to implementation of bioinformatics into life sciences education. Front Educ. 2023;8:1317191. 10.3389/feduc.2023.1317191

[bib24] Duarte ASR, Stärk KDC, Munk P et al. Addressing learning needs on the use of metagenomics in antimicrobial resistance surveillance. Front Public Health. 2020;8:38. 10.3389/fpubh.2020.00038 32158739 PMC7051937

[bib25] Dunivin TK, Yeh SY, Shade A. A global survey of arsenic-related genes in soil microbiomes. BMC Biol. 2019;17:45. 10.1186/s12915-019-0661-5. 31146755 PMC6543643

[bib26] Ghisleni G, Armanni A, Fumagalli S et al. The Bicocca sampling days model: a participatory citizen science approach to environmental microbiome research and education. ISME Commun. 2025;5:ycaf220. 10.1093/ismeco/ycaf220. 41403706 PMC12704432

[bib27] Ghisleni G, Dow ED, Iovino T et al. From Reads to Function Workshop—Milano 2026. U.S. Department of Energy Systems Biology Knowledgebase (KBASE), 15 July 2026. 10.25982/243825.51/3374872

[bib28] Goller CC, Ott LE. Evolution of an 8-week upper-division metagenomics course: diagramming a learning path from observational to quantitative microbiome analysis. Biochem Molecular Bio Educ. 2020;48:391–403. 10.1002/bmb.21349. 32294307

[bib29] Goller CC, Srougi MC, Chen SH et al. Integrating bioinformatics tools into inquiry-based Molecular biology laboratory education modules. Front Educ. 2021;6:711403. 10.3389/feduc.2021.711403 PMC8758113 35036827

[bib30] Govindan B, Pickett S, Riggs B. Fear of the CURE: a beginner’s guide to overcoming barriers in creating a course-based undergraduate research experience. J Microbiol Biol Educ. 2020;21:50. 10.1128/jmbe.v21i2.2109. PMC7243983 32528607

[bib31] Helmy M, Awad M, Mosa KA. Limited resources of genome sequencing in developing countries: challenges and solutions. Appl Transl Genom. 2016;9:15–19. 10.1016/j.atg.2016.03.003. 27354935 PMC4911431

[bib32] Ho Pao C, Choi SCT, Lok SY et al. Inquiry-driven bioinformatics laboratory research module: metagenomic study of student oral microbes. J Microbiol Biol Educ. 2021;22:1–10. 10.1128/jmbe.00136-21 PMC8442007 34594441

[bib33] Huang YN, Munteanu V, Love MI et al. Perceptual and technical barriers in sharing and formatting metadata accompanying omics studies. Cell Genomics. 2025;5:100845. 10.1016/j.xgen.2025.100845. 40215974 PMC12143318

[bib34] Hyatt D, Chen GL, Locascio PF et al. Prodigal: prokaryotic gene recognition and translation initiation site identification. BMC Bioinf. 2010;11:119. 10.1186/1471-2105-11-119. PMC2848648 20211023

[bib35] Işık EB, Brazas MD, Schwartz R et al. Grand challenges in bioinformatics education and training. Nat Biotechnol. 2023;41:1171–4. 10.1038/s41587-023-01891-9. 37568018

[bib36] Jurkowski A, Reid AH, Labov JB. Metagenomics: a call for bringing a new science into the classroom (While It’s Still New). LSE. 2007;6:260–5. 10.1187/cbe.07-09-0075. PMC2104496 18056294

[bib37] Kang DD, Froula J, Egan R et al. MetaBAT, an efficient tool for accurately reconstructing single genomes from complex microbial communities. PeerJ. 2015;3:e1165. 10.7717/peerj.1165. 26336640 PMC4556158

[bib38] Kruchten AE. A curricular bioinformatics approach to teaching undergraduates to analyze metagenomic datasets using R. Front Microbiol. 2020;11:578600. 10.3389/fmicb.2020.578600 33013816 PMC7511545

[bib39] Li D, Liu CM, Luo R et al. MEGAHIT: an ultra-fast single-node solution for large and complex metagenomics assembly via succinct de Bruijn graph. Bioinform (Oxf, Engl). 2015;31:1674–6. 10.1093/bioinformatics/btv033. 25609793

[bib40] Lorimer J, Hodgetts T, Grenyer R et al. Making the microbiome public: participatory experiments with DNA sequencing in domestic kitchens. Trans Inst British Geog. 2019;44:524–41. 10.1111/tran.12289.

[bib41] Medema MH, Blin K, Cimermancic P et al. antiSMASH: rapid identification, annotation and analysis of secondary metabolite biosynthesis gene clusters in bacterial and fungal genome sequences. Nucleic Acids Res. 2011;39:W339–346. 10.1093/nar/gkr466 21672958 PMC3125804

[bib42] Morsink MC, Schaik ENv, Bossers K et al. Metagenomics education in a modular CURE format positively affects students’ scientific discovery perception and data analytical skills. Biochem Molecular Bio Educ. 2025;53:311–20. 10.1002/bmb.21888. 39876557

[bib43] Muth TR, Caplan AJ. Microbiomes for all. Front Microbiol. 2020;11:593472. 10.3389/fmicb.2020.593472 33281791 PMC7688743

[bib44] Overbeek R, Olson R, Pusch GD et al. The SEED and the rapid annotation of microbial genomes using Subsystems Technology (RAST). Nucl Acids Res. 2014;42:D206–14. 10.1093/nar/gkt1226. 24293654 PMC3965101

[bib45] Parks DH, Imelfort M, Skennerton CT et al. CheckM: assessing the quality of microbial genomes recovered from isolates, single cells, and metagenomes. Genome Res. 2015;25:1043–55. 10.1101/gr.186072.114. 25977477 PMC4484387

[bib46] Parks S, Joyner JL, Nusnbaum M. Reaching a large urban undergraduate population through microbial ecology course-based research experiences. J Microbiol Biol Educ. 2020;21:1–10. 10.1128/jmbe.v21i1.2047 PMC7148149 32313597

[bib47] Phillips T, Porticella N, Bonney R. Skills for Science Inquiry Scale (Custom). Technical Brief Series. Ithaca NY: Cornell Lab of Ornithology, 2017.

[bib48] Porticella N, Phillips T, Bonney R. Self-Efficacy for Learning and Doing Science Scale (SELDS, Custom). Technical Brief Series. Ithaca NY: Cornell Lab of Ornithology, 2017.

[bib49] Quessada MP, Clément P. An epistemological approach to French Syllabi on Human origins during the 19th and 20th centuries. Sci & Educ. 2007;16:991–1006. 10.1007/s11191-006-9051-9.

[bib50] Rosen GL, Hammrich P. Teaching microbiome analysis: from design to computation through inquiry. Front Microbiol. 2020;11:528051. 10.3389/fmicb.2020.528051 33193120 PMC7658192

[bib51] Rueangmongkolrat N, Uthaipaisanwong P, Kusonmano K et al. The role of microbiomes in cooperative detoxification mechanisms of arsenate reduction and arsenic methylation in surface agricultural soil. PeerJ. 2024;12:e18383. 10.7717/peerj.18383. 39494289 PMC11531259

[bib52] Sanders ER, Hirsch AM. Immersing undergraduate students into research on the metagenomics of the plant rhizosphere: a pedagogical strategy to engage civic-mindedness and retain undergraduates in STEM. Front Plant Sci. 2014;5:1–4. 10.3389/fpls.2014.00157 PMC4012186 24817868

[bib53] Schmieder R, Edwards R. Quality control and preprocessing of metagenomic datasets. Bioinform (Oxf, Engl). 2011;27:863–4. 10.1093/bioinformatics/btr026. PMC3051327 21278185

[bib54] Shaffer M, Borton MA, McGivern BB et al. DRAM for distilling microbial metabolism to automate the curation of microbiome function. Nucleic Acids Res. 2020;48:8883–900. 10.1093/nar/gkaa621. 32766782 PMC7498326

[bib55] Shortlidge EE, Bangera G, Brownell SE. Faculty perspectives on developing and teaching course-based undergraduate research experiences. Bioscience. 2016;66:54–62. 10.1093/biosci/biv167.

[bib56] Sieber CMK, Probst AJ, Sharrar A et al. Recovery of genomes from metagenomes via a dereplication, aggregation and scoring strategy. Nat Microbiol. 2018;3:836–43. 10.1038/s41564-018-0171-1. 29807988 PMC6786971

[bib57] Swetnam TL, Antin PB, Bartelme R et al. CyVerse: cyberinfrastructure for open science. PLoS Comput Biol. 2024;20:e1011270. 10.1371/journal.pcbi.1011270. 38324613 PMC10878509

[bib58] The Galaxy Community . The Galaxy platform for accessible, reproducible, and collaborative data analyses: 2024 update. Nucleic Acids Res. 2024;52:W83–94. 10.1093/nar/gkae410. 38769056 PMC11223835

[bib59] Tuononen T, Hyytinen H, Räisänen M et al. Metacognitive awareness in relation to university students’ learning profiles. Metacognition Learn. 2023;18:37–54. 10.1007/s11409-022-09314-x.

[bib60] Uzorka A, Akiyode O, Isa SM. Strategies for engaging students in sustainability initiatives and fostering a sense of ownership and responsibility towards sustainable development. Discov Sustain. 2024;5:320. 10.1007/s43621-024-00505-x.

[bib61] Wang JTH. Course-based undergraduate research experiences in molecular biosciences—Patterns, trends, and faculty support. FEMS Microbiol Lett. 2017;364:fnx157. 10.1093/femsle/fnx157. 28859321

[bib62] Weng X, Chiu TKF, Tsang CC. Promoting student creativity and entrepreneurship through real-world problem-based maker education. Think Skills Creat. 2022;45:101046. 10.1016/j.tsc.2022.101046.

[bib63] Whitham JM, Goller CC. Improving knowledge of metagenome-assembled genomes (MAGs) through bioinformatics and article annotation. J Microbiol Biol Educ. 2026;27:e00226–25. 10.1128/jmbe.00226-25. 41744501 PMC13131013

[bib64] Wood-Charlson EM, Henry CS, Dehal PS et al. KBase: open-source Platform for Collaborative Biological Data Analysis and Publication. J Mol Biol. 2026;438:169676. 10.1016/j.jmb.2026.169676 41651014

[bib65] Wu YW, Simmons BA, Singer SW. MaxBin 2.0: an automated binning algorithm to recover genomes from multiple metagenomic datasets. Bioinformatics. 2016;32:605–7. 10.1093/bioinformatics/btv638. 26515820

[bib66] Wu YW, Tang YH, Tringe SG et al. MaxBin: an automated binning method to recover individual genomes from metagenomes using an expectation-maximization algorithm. Microbiome. 2014;2:26. 10.1186/2049-2618-2-26. 25136443 PMC4129434

