# Supplementary material for: Fantastic microbes and where to find them: evaluating learning-by-doing outcomes in a crowdfunded metagenomics workshop
Source: FEMS Microbiol Lett. 2026 Aug 17;373:fnag093. doi: 10.1093/femsle/fnag093 (PMC13501537; doi:10.1093/femsle/fnag093)
Supplement: Supplementary file 1 [file fnag093_supplemental_files.zip › 1_SupplMat.pdf]

# From Reads to Function Workshop - Milano 2026

## BSD4_211B

Sample: Vivaio Bicocca, 27.04.2024

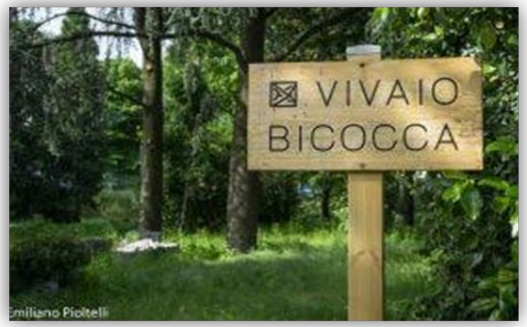


**Matilde Marzucchi**
**Yodit Bacchi**

# _Quality Control_

## Summary

- Basic Statistics
- Per base sequence quality
- Per tile sequence quality
- Per sequence quality scores
- Per base sequence content
- Per sequence GC content
- Per base N content
- Sequence Length Distribution
- Sequence Duplication Levels
- Overrepresented sequences
- Adapter Content

Overrepresented sequences

| Sequence | Count | Percentage | Possible Source |
|---|---|---|---|
| GGGGGGGGGGGGGGGGGGGGGGGGGGGGGGGGGGGGGGGGGGGGGGGGGG | 447599 | 1.444178892607217 | No Hit |

**We removed the PolyG tayls trough PRINSEQ**

# _Quality Control_

## Summary

- Basic Statistics
- Per base sequence quality
- Per tile sequence quality
- Per sequence quality scores
- Per base sequence content
- Per sequence GC content
- Per base N content
- Sequence Length Distribution
- Sequence Duplication Levels
- Overrepresented sequences
- Adapter Content

## Per base sequence quality

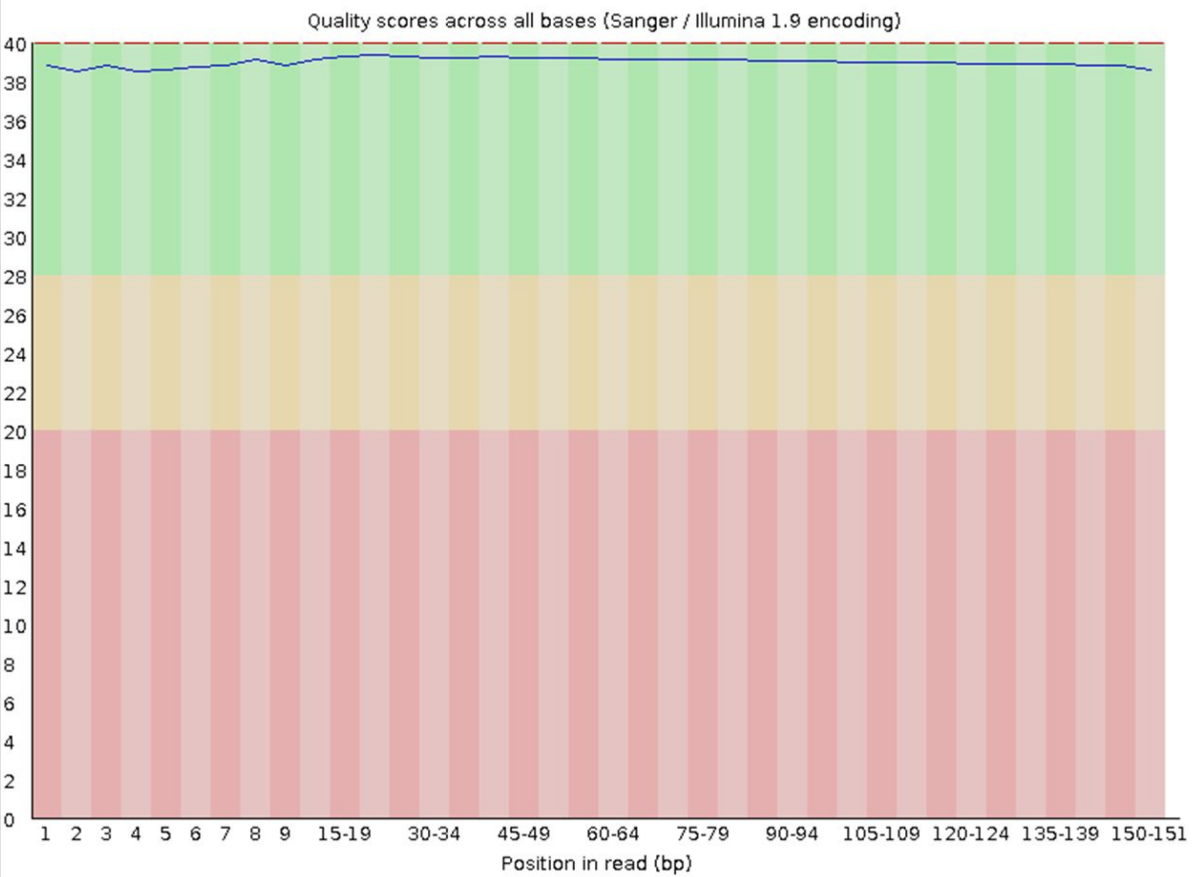

# _Assembly_

**We assembled the reads into contiguous fragments (contigs) to create the scaffolding of the whole genome using these four metagenomics assemblers.**

meta SPAdes

MEGA HIT

sensitive

MEGA HIT

large

IDBA-UD

# _Assembly_ Quality of Assembly _

| Statistics without reference | BSD4_211B_prinseq_MEGAHIT-sen... | BSD4_211B_prinseq_MEGAHIT-lar... | BSD4_211Bprinseq_metaSPAdes | BSD4_211B_prinseq_IDBA-UD |
|---|---|---|---|---|
| # contigs | 654 | 629 | 524 | 57 |
| # contigs (>= 0 bp) | 654 | 629 | 524 | 57 |
| # contigs (>= 1000 bp) | 654 | 629 | 524 | 57 |
| # contigs (>= 10000 bp) | 0 | 0 | 0 | 0 |
| # contigs (>= 100000 bp) | 0 | 0 | 0 | 0 |
| # contigs (>= 1000000 bp) | 0 | 0 | 0 | 0 |
| Largest contig | 5940 | 6784 | 6935 | 4312 |
| Total length | 1 618 235 | 1 572 527 | 1 342 598 | 149 770 |
| Total length (>= 0 bp) | 1 618 235 | 1 572 527 | 1 342 598 | 149 770 |
| Total length (>= 1000 bp) | 1 618 235 | 1 572 527 | 1 342 598 | 149 770 |
| Total length (>= 10000 bp) | 0 | 0 | 0 | 0 |
| Total length (>= 100000 bp) | 0 | 0 | 0 | 0 |
| Total length (>= 1000000 bp) | 0 | 0 | 0 | 0 |
| N50 | 2386 | 2398 | 2510 | 2515 |
| N75 | 2146 | 2156 | 2173 | 2227 |
| L50 | 279 | 266 | 219 | 24 |
| L75 | 459 | 440 | 363 | 40 |
| GC (%) | 61.95 | 63.39 | 61.85 | 54.14 |
| **Mismatches** | | | | |
| # N's | 0 | 0 | 300 | 0 |
| # N's per 100 kbp | 0 | 0 | 22.34 | 0 |
| **Predicted genes** | | | | |
| # predicted genes (unique) | 1599 | 1580 | 1339 | 121 |
| # predicted genes (>= 0 bp) | 1355 + 244 part | 1293 + 287 part | 1123 + 216 part | 104 + 17 part |
| # predicted genes (>= 300 bp) | 1162 + 224 part | 1116 + 263 part | 965 + 201 part | 82 + 16 part |
| # predicted genes (>= 1500 bp) | 140 + 31 part | 138 + 34 part | 101 + 35 part | 9 + 2 part |
| # predicted genes (>= 3000 bp) | 4 + 0 part | 0 + 2 part | 1 + 1 part | 0 + 0 part |

# _Binning _ metaSPAdes_

CONCOCT

Binned contigs: 221

Input contigs: 524

Number of bins: 15

MetaBAT2

Binned contigs: 155

Input contigs: 524

Number of bins: 1

**Having assembled the contigs, the next step is to cluster them into bins based on similarity. Each bin corresponds to a putative population genome.**
**Having binned the contigs with three methods, the next step is to improve the quality of the binning using consensus assignments.**

MaxBin2

Bins: 2

Input Contigs: 524

Binned Contigs: 494 (94.3%)

Unbinned Contigs: 30 (5.7%)

Contigs Too Short: 0 (0.0%)

Summed Length of Binned Contigs: 1268702 (94.5%)

Summed Length of Unbinned Contigs: 73896 (5.5%)

Summed Length of Short Contigs: 0 (0.0%)

**DAS tool**

Binned contigs: 65

Input contigs: 524

Number of bins: 1

# _Bi[illegible]ing _ metaSPAdes_

CONCOCT

Binned co[illegible]: 221

Input contigs: 524

Number of bins: 15

MetaBAT2

Binned contigs: 155

Input contigs: 524

N[illegible]er of bins: 1

**Having assembled the contigs, the next step is [illegible] cluster them into bins based on similarit[illegible]ach bin corresponds to a putative po[illegible]ation genome.**
**Having binned the contigs [illegible] three methods, the next step is to im[illegible]ove the quality of the binning usin[illegible]nsensus assignments.**

MaxBin2

Bins: 2

Input Contigs: 524

Binned Contigs: 494 (94.3%)

Unbinned Contigs: 30 (5.7%)

Contigs Too Short: 0 (0.0%)

Summed Length of Binned Contig[illegible]8702 (94.5%)

Summed Length of Unbin[illegible]ontigs: 73896 (5.5%)

Summed Length [illegible]ort Contigs: 0 (0.0%)

**DAS tool**

Binne[illegible]ontigs: 65

Input contigs: [illegible]

Number of bins: 1

# _Assembly_ Quality of Assembly _

| Statistics without reference | BSD4_211B_prinseq_MEGAHIT-sen... | BSD4_211B_prinseq_MEGAHIT-lar... | BSD4_211Bprinseq_metaSPAdes | BSD4_211B_prinseq_IDBA-UD |
|---|---|---|---|---|
| # contigs | 654 | 629 | 524 | 57 |
| # contigs (>= 0 bp) | 654 | 629 | 524 | 57 |
| # contigs (>= 1000 bp) | 654 | 629 | 524 | 57 |
| # contigs (>= 10000 bp) | 0 | 0 | 0 | 0 |
| # contigs (>= 100000 bp) | 0 | 0 | 0 | 0 |
| # contigs (>= 1000000 bp) | 0 | 0 | 0 | 0 |
| Largest contig | 5940 | 6784 | 6935 | 4312 |
| Total length | 1 618 235 | 1 572 527 | 1 342 598 | 149 770 |
| Total length (>= 0 bp) | 1 618 235 | 1 572 527 | 1 342 598 | 149 770 |
| Total length (>= 1000 bp) | 1 618 235 | 1 572 527 | 1 342 598 | 149 770 |
| Total length (>= 10000 bp) | 0 | 0 | 0 | 0 |
| Total length (>= 100000 bp) | 0 | 0 | 0 | 0 |
| Total length (>= 1000000 bp) | 0 | 0 | 0 | 0 |
| N50 | 2386 | 2398 | 2510 | 2515 |
| N75 | 2146 | 2156 | 2173 | 2227 |
| L50 | 279 | 266 | 219 | 24 |
| L75 | 459 | 440 | 363 | 40 |
| GC (%) | 61.95 | 63.39 | 61.85 | 54.14 |
| **Mismatches** | | | | |
| # N's | 0 | 0 | 300 | 0 |
| # N's per 100 kbp | 0 | 0 | 22.34 | 0 |
| **Predicted genes** | | | | |
| # predicted genes (unique) | 1599 | 1580 | 1339 | 121 |
| # predicted genes (>= 0 bp) | 1355 + 244 part | 1293 + 287 part | 1123 + 216 part | 104 + 17 part |
| # predicted genes (>= 300 bp) | 1162 + 224 part | 1116 + 263 part | 965 + 201 part | 82 + 16 part |
| # predicted genes (>= 1500 bp) | 140 + 31 part | 138 + 34 part | 101 + 35 part | 9 + 2 part |
| # predicted genes (>= 3000 bp) | 4 + 0 part | 0 + 2 part | 1 + 1 part | 0 + 0 part |

# _Binning_ MEGAHIT sensitive_

**DAS tool**

Binned contigs: 530

Input contigs: 654

Number of bins: 1

CONCOCT

Binned contigs: 215

Input contigs: 654

Number of bins: 7

MaxBin2

Bins: 2

Input Contigs: 654

Binned Contigs: 619 (94.6%)

Unbinned Contigs: 35 (5.4%)

Contigs Too Short: 0 (0.0%)

Summed Length of Binned Contigs: 1531872 (94.7%)

Summed Length of Unbinned Contigs: 86363 (5.3%)

Summed Length of Short Contigs: 0 (0.0%)

MetaBAT2

Binned contigs: 118

Input contigs: 654

Number of bins: 1

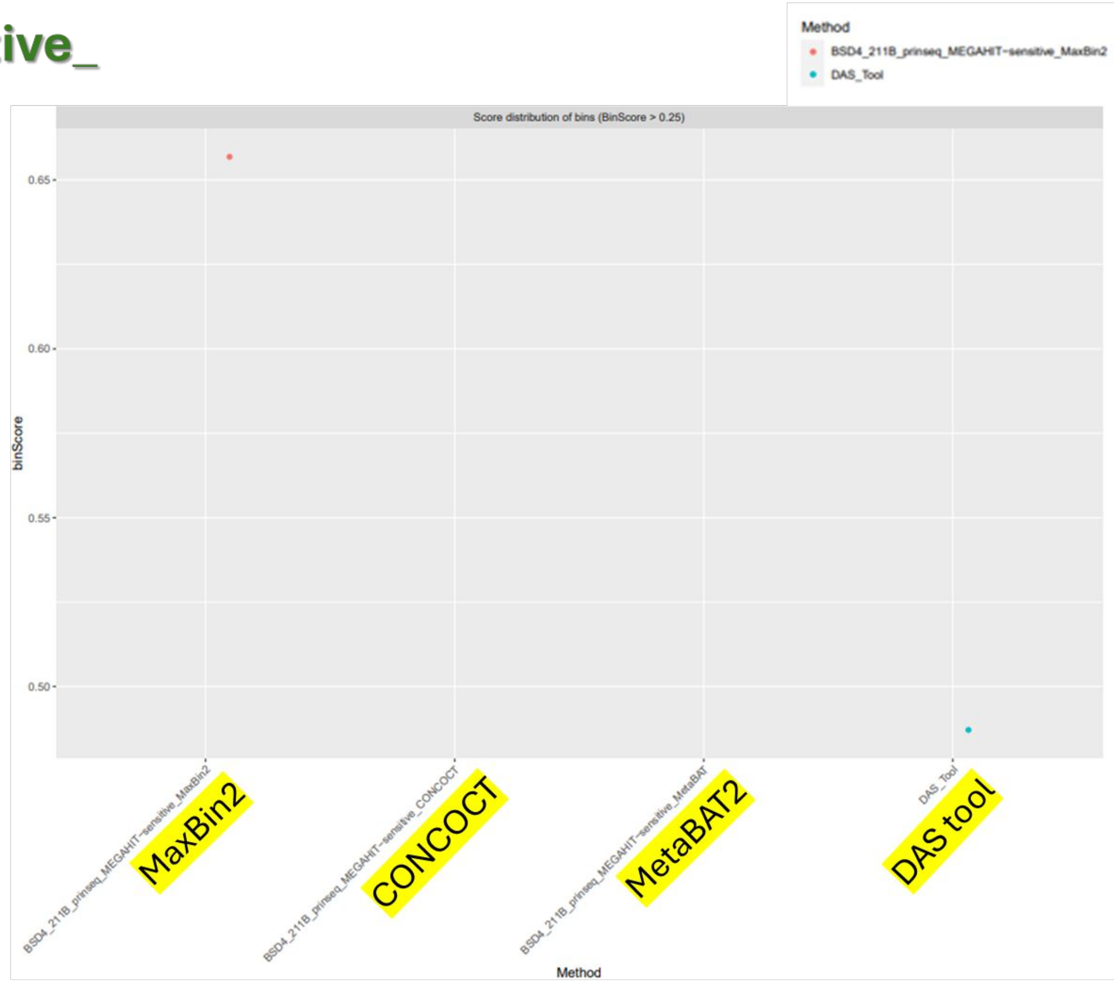

# _Bin Quality Control _

| | |
|---|---|
| KBase Object Name | bin.001.fastaBSD4_211B_prinseq_MEGAHIT-sensitive_dastool_assembly |
| Number of Contigs | 530 |
| Total GC Content | 65.74% |
| Total Length | 1,307,740 bp |

**CheckM PLOT** | CheckM Table

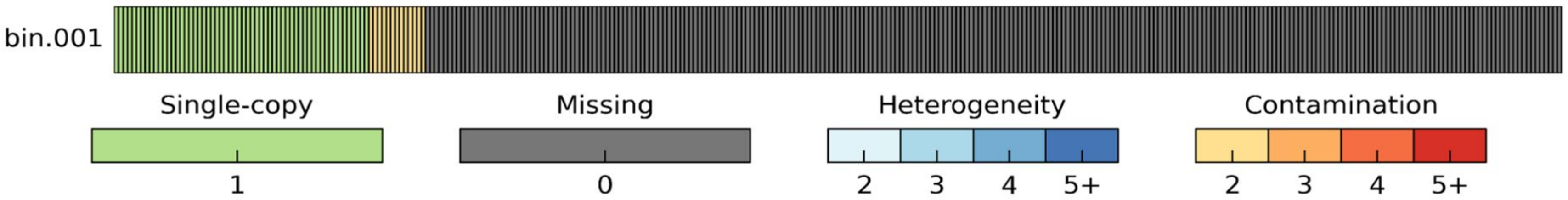


**Quality control is a necessary step at every level of analysis to ensure the highest quality outcome and to avoid error propagation.**

**THRESHOLD:**
**>50% completeness**
**<10% contamination**

CheckM Plot | **CheckM TABLE**

| Bin Name | Marker Lineage | # Genomes | # Markers | # Marker Sets | 0 | 1 | 2 | 3 | 4 | 5+ | Completeness | Contamination |
|---|---|---|---|---|---|---|---|---|---|---|---|---|
| bin.001 | o__Actinomycetales | 387 | 330 | 193 | 259 | 58 | 13 | 0 | 0 | 0 | 16.31 | 2.44 |

# _ Functional Annotation_ DRAM_

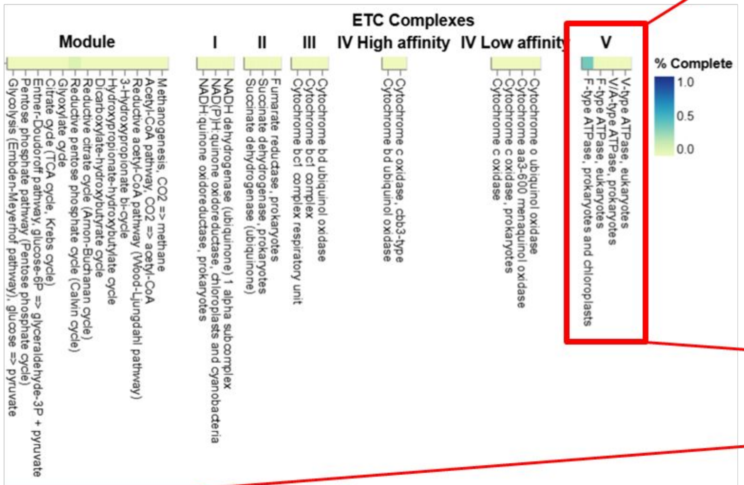


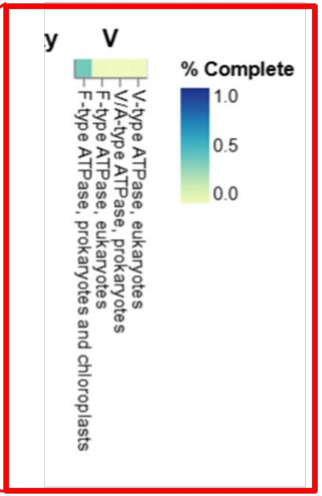


**Functional Annotation usign a genome annotation tool to investigate metabolic functional capacity.**

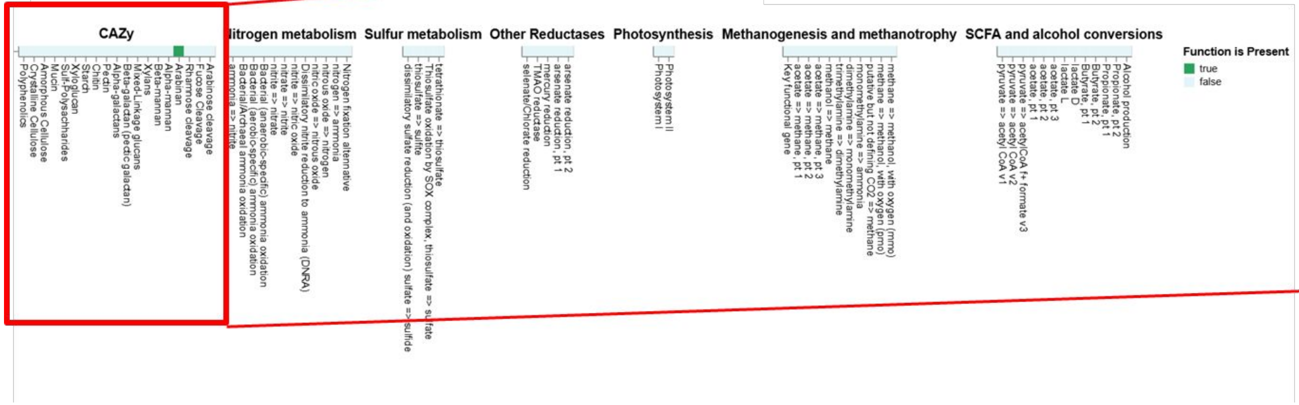


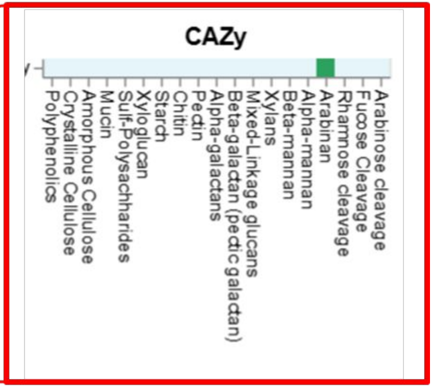

# _ Functional Annotation_
# HMMs of MicroTrait Bioelents _

| Nitrogen | C1 Compounds | Oxygen | Hydrogen | | |
|---|---|---|---|---|---|
| nirD | pqqC | cydA | hydrogenase_NiFeGroup4d | hydrogenase_NiFeGroup4h | hydrogenase_NiFeGroup4i |
| ● | ● | ● | ● | ● | ● |

| ALIGNMENT COVERAGE (HIT SEQ) | GENE ID | FUNCTION | GENOME | ALN_LEN | i-EVALUE | BIT SCORE | H_BEG-H_END |
|---|---|---|---|---|---|---|---|
| Hits to **nirD** | | | | | | | |
| | CDS.999 | Nitrite reductase [NAD(P)H] small subunit (EC 1.7.1.4) | bin.001.fastaBSD4_211B_prinseq_MEGAHIT-sensitive_dastool_assembly.RAST | 123 (82.0%) | 7.4e-39 | 129.4 | 1-119 |
| Hits to **pqqC** | | | | | | | |
| | CDS.48 | Pyrroloquinoline-quinone synthase (EC 1.3.3.11) | bin.001.fastaBSD4_211B_prinseq_MEGAHIT-sensitive_dastool_assembly.RAST | 240 (98.4%) | 1.1e-103 | 342.7 | 2-239 |
| Hits to **cydA** | | | | | | | |
| | CDS.297 | Cytochrome d ubiquinol oxidase subunit I (EC 1.10.3.-) | bin.001.fastaBSD4_211B_prinseq_MEGAHIT-sensitive_dastool_assembly.RAST | 310 (63.8%) | 2e-140 | 464.9 | 1-316 |
| Hits to **hydrogenase_NiFeGroup4d** | | | | | | | |
| | CDS.611 | NADH-ubiquinone oxidoreductase chain D (EC 1.6.5.3) | bin.001.fastaBSD4_211B_prinseq_MEGAHIT-sensitive_dastool_assembly.RAST | 260 (63.1%) | 4.3e-46 | 154.0 | 41-294 |
| Hits to **hydrogenase_NiFeGroup4h** | | | | | | | |
| | CDS.611 | NADH-ubiquinone oxidoreductase chain D (EC 1.6.5.3) | bin.001.fastaBSD4_211B_prinseq_MEGAHIT-sensitive_dastool_assembly.RAST | 252 (67.4%) | 5.4e-46 | 153.8 | 42-295 |
| Hits to **hydrogenase_NiFeGroup4i** | | | | | | | |
| | CDS.611 | NADH-ubiquinone oxidoreductase chain D (EC 1.6.5.3) | bin.001.fastaBSD4_211B_prinseq_MEGAHIT-sensitive_dastool_assembly.RAST | 256 (68.4%) | 2.4e-32 | 108.7 | 41-294 |

# _Taxonomy Classification_

**GTDB trees**

gtdbtk.backbone.bac120.classify-trimmed.tree

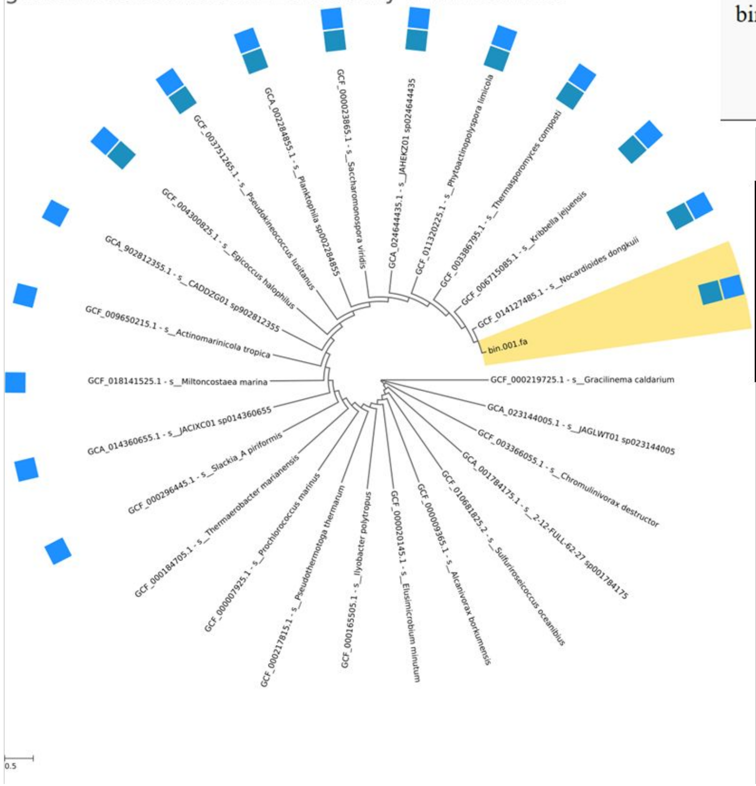


| User Genome | Classification | FastANI Reference | FastANI Reference Radius | FastANI Taxonomy | FastANI ANI | FastANI Alignment Fraction |
|---|---|---|---|---|---|---|
| bin.001.fa | d__Bacteria;<br>p__Actinomycetota;<br>c__Actinomycetia;<br>o__;<br>f__;<br>g__;<br>s__ | - | - | - | - | - |

| Closest Placement Reference | Closest Placement Taxonomy | Closest Placement ANI | Closest Placement Alignment Fraction |
|---|---|---|---|
| - | - | - | - |

**Phylogenetic taxonomy classification of the MAGs. The lowest taxonomy level is the class: Actinomycetia.**

| Classification Method | Note | Other Related References | MSA AA Percent | RED Value | Warnings |
|---|---|---|---|---|---|
| taxonomic classification fully defined by topology | classification based on placement in class-level tree | - | 18.81 | 0.52629 | - |

# _ Taxonomy Classification_

**GTDB trees**

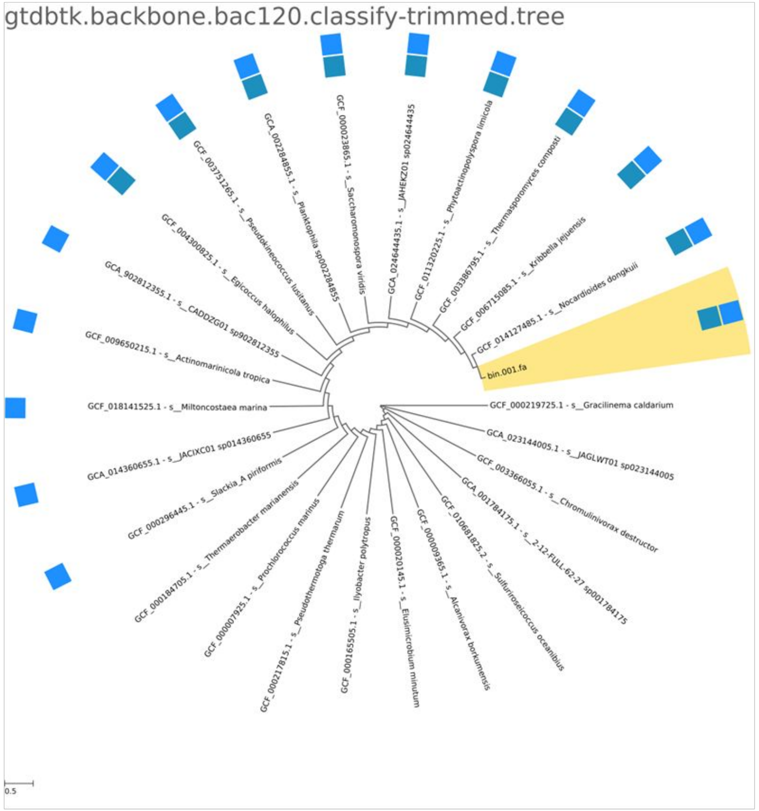


**GTDB trees is coherent with the KAIJU output**

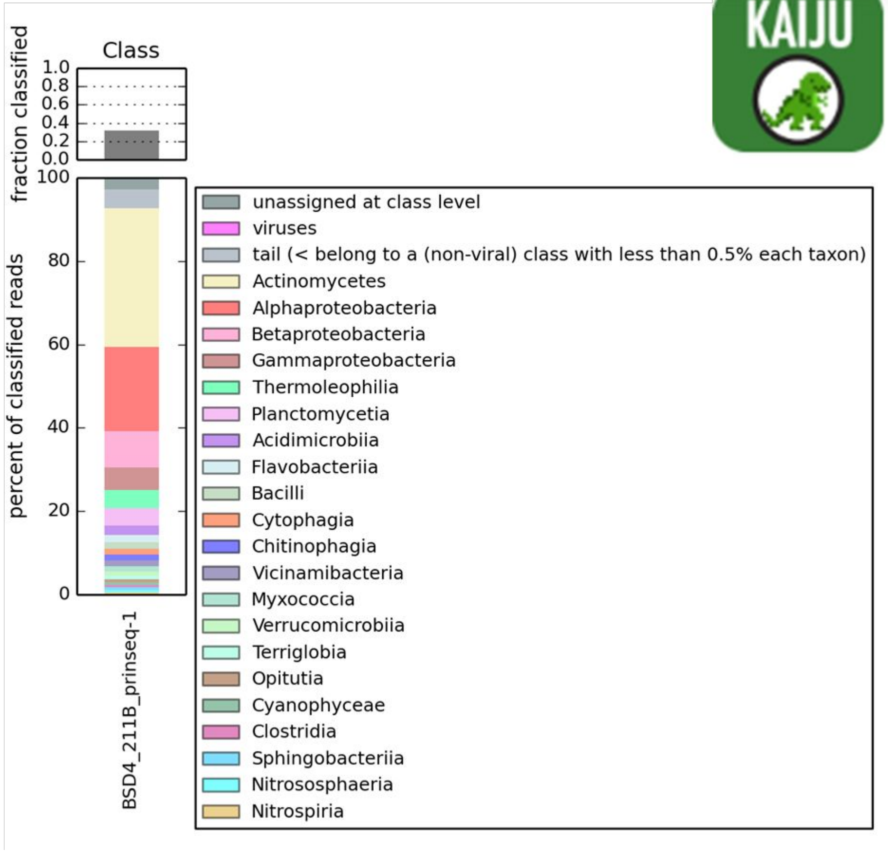

# _ Taxonomy Classification _ Build a Phylogenetic Trees _

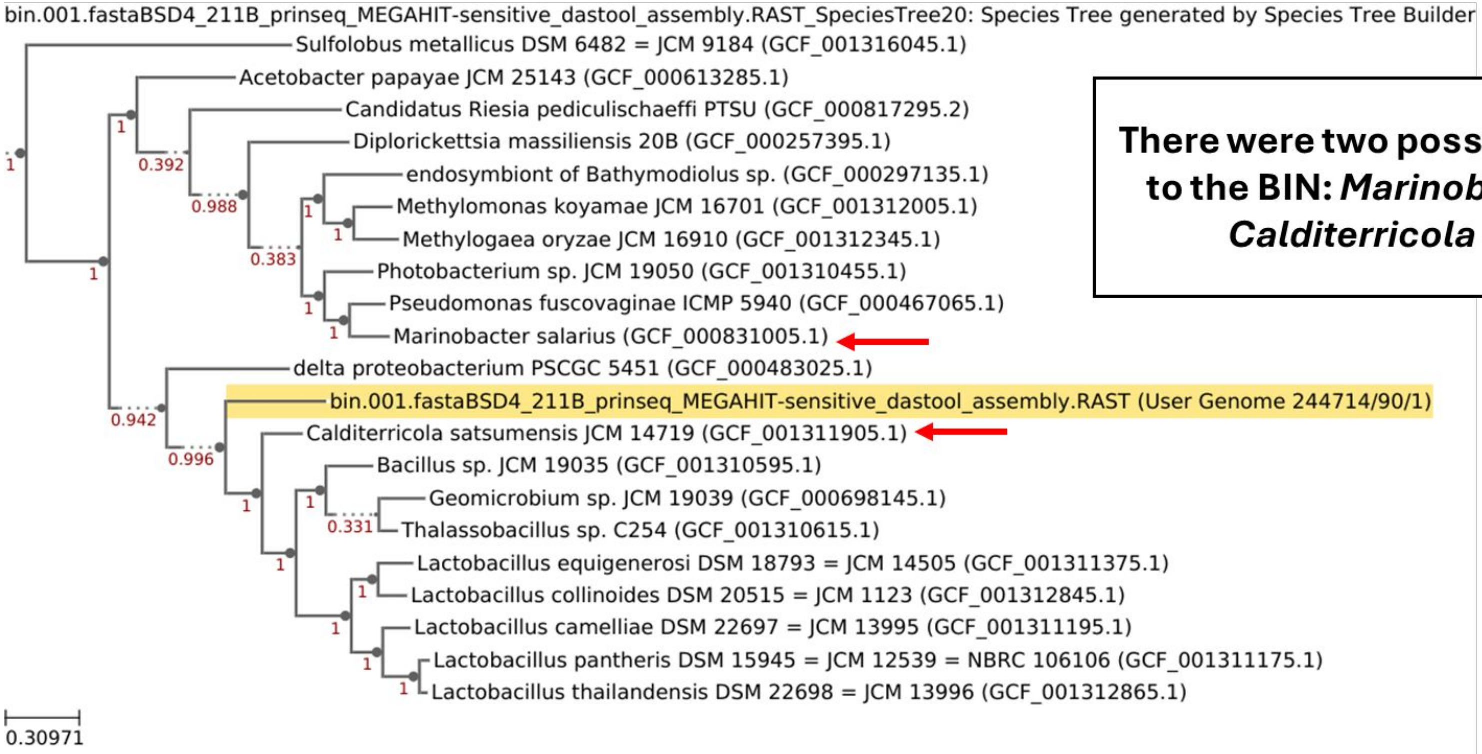


**There were two possibly species closest to the BIN: *Marinobacter salarius* and *Calditerricola astsumensis.***

# _ Taxonomy Classification _ Build a Phylogenetic Trees _

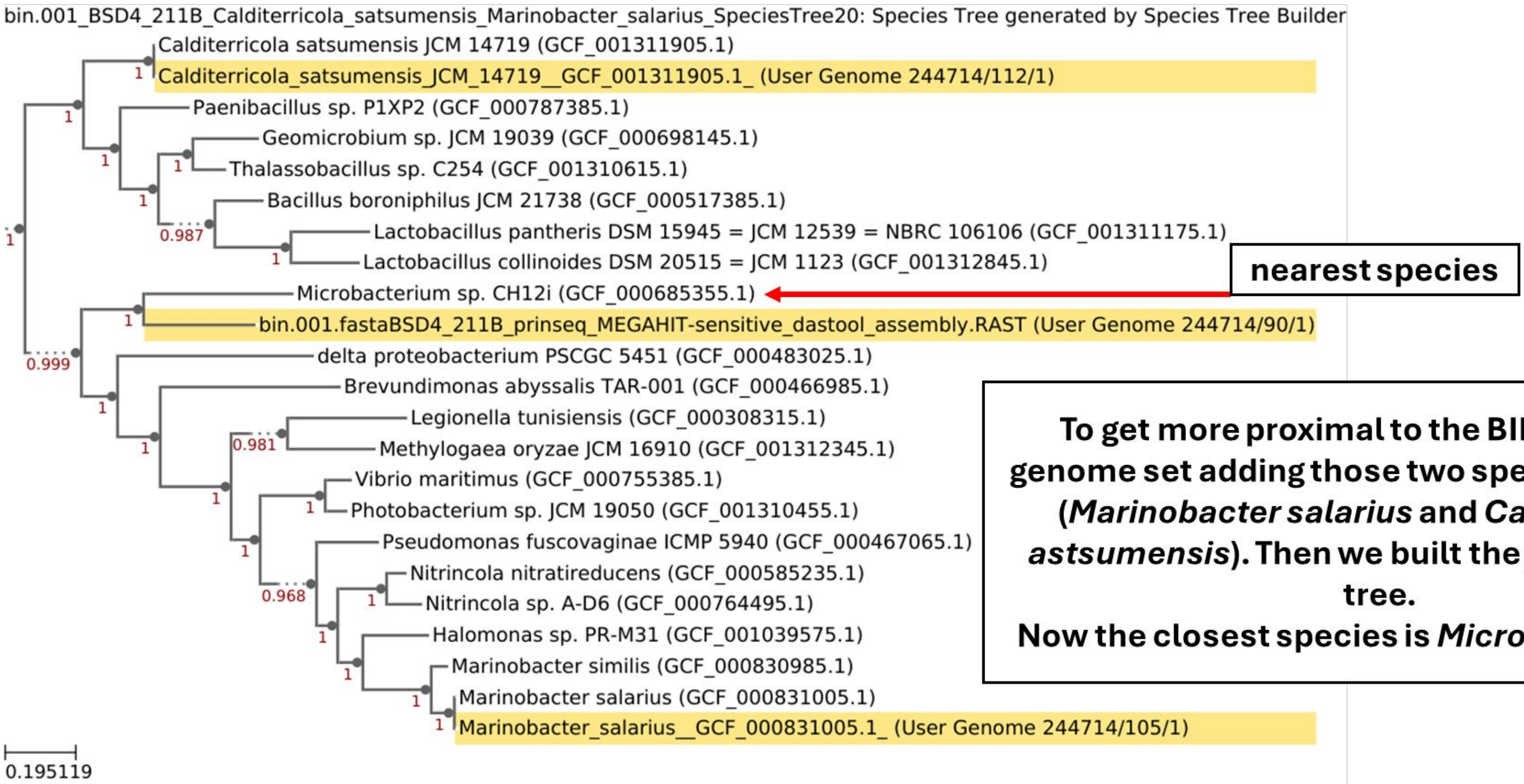


**To get more proximal to the BIN we built a genome set adding those two species to the run (*Marinobacter salarius* and *Calditerricola astsumensis*). Then we built the phylogenetic tree.**
**Now the closest species is *Microbacterium sp.***

# _Comparison with BDS4 co-assembly_

**BDS4_211B**

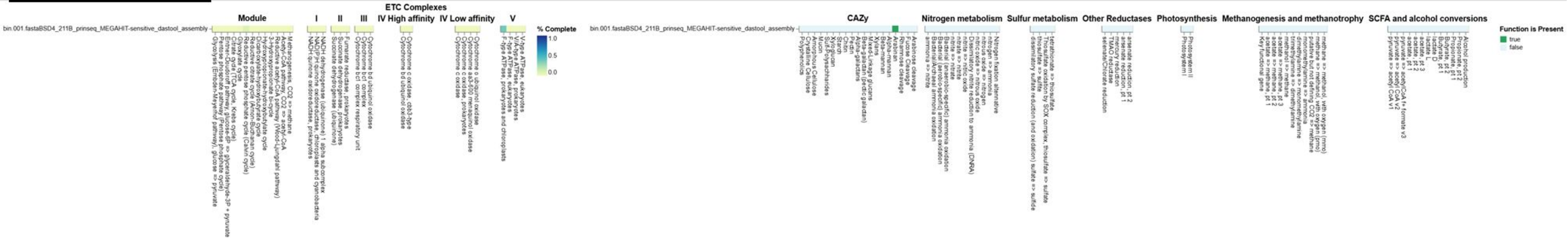


**BDS4_co-assembly**

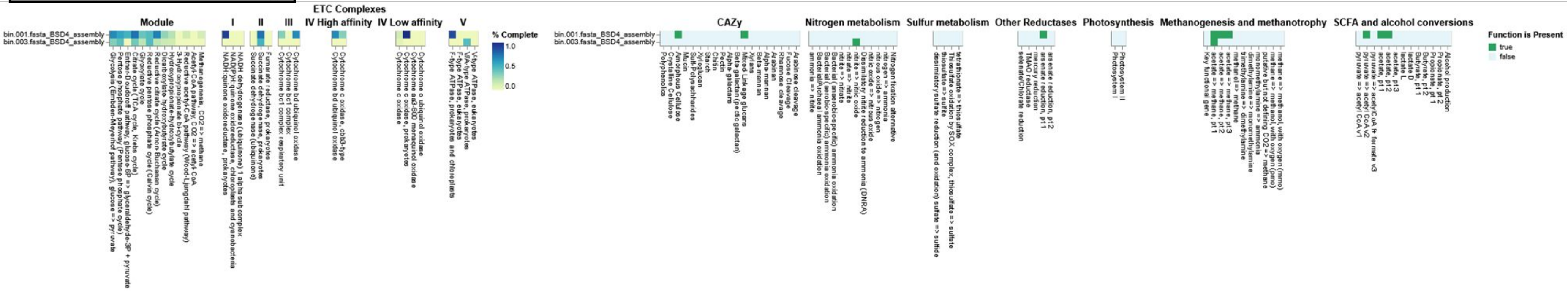

# _Comparison with BDS4  co-assembly_

**We found a taxa that actually is not present in the co-assembly.**

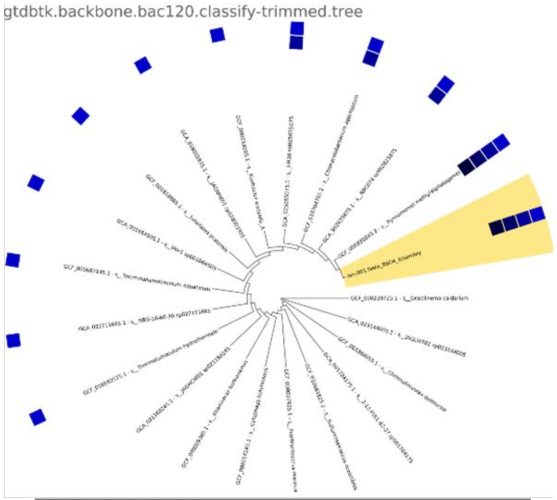


## BDS4_211B

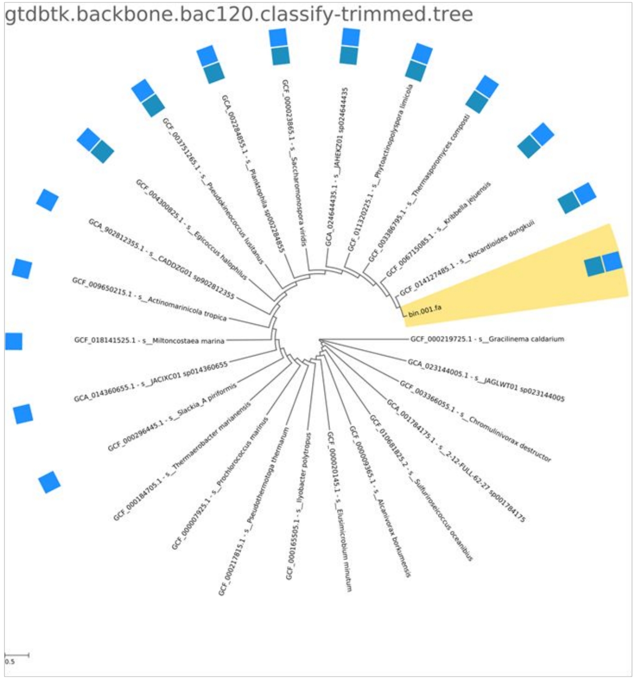


## BDS4_co-assembly

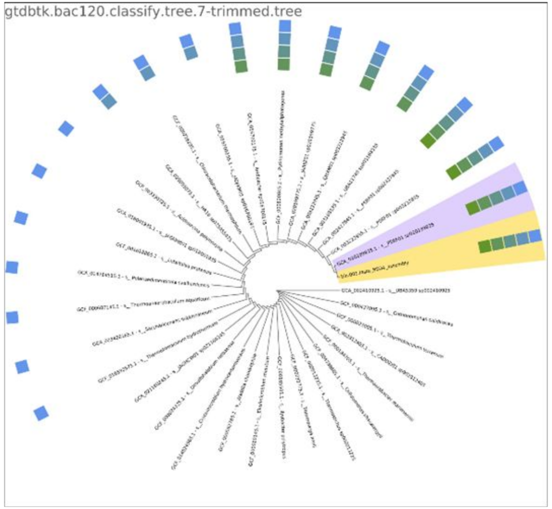


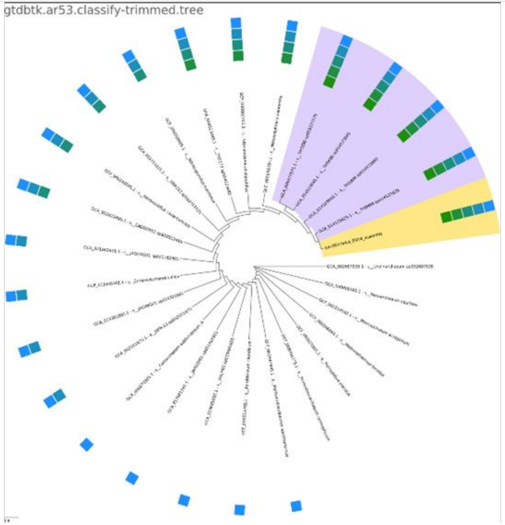

# _Comparison with BDS4 co-assembly_

**BDS4_co-assembly**

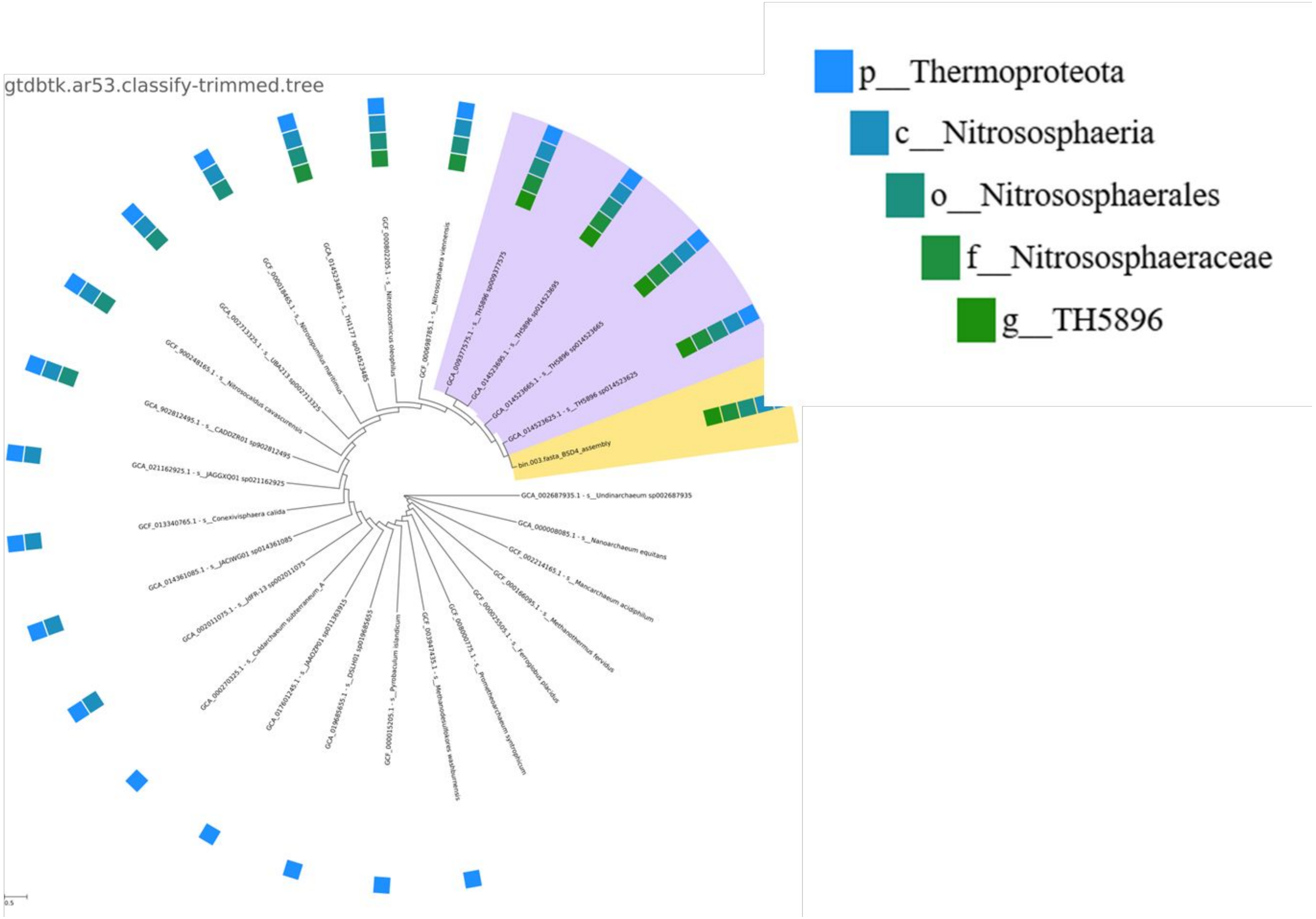

# _Comparison with BDS4  co-assembly_

**BDS4_co-assembly**

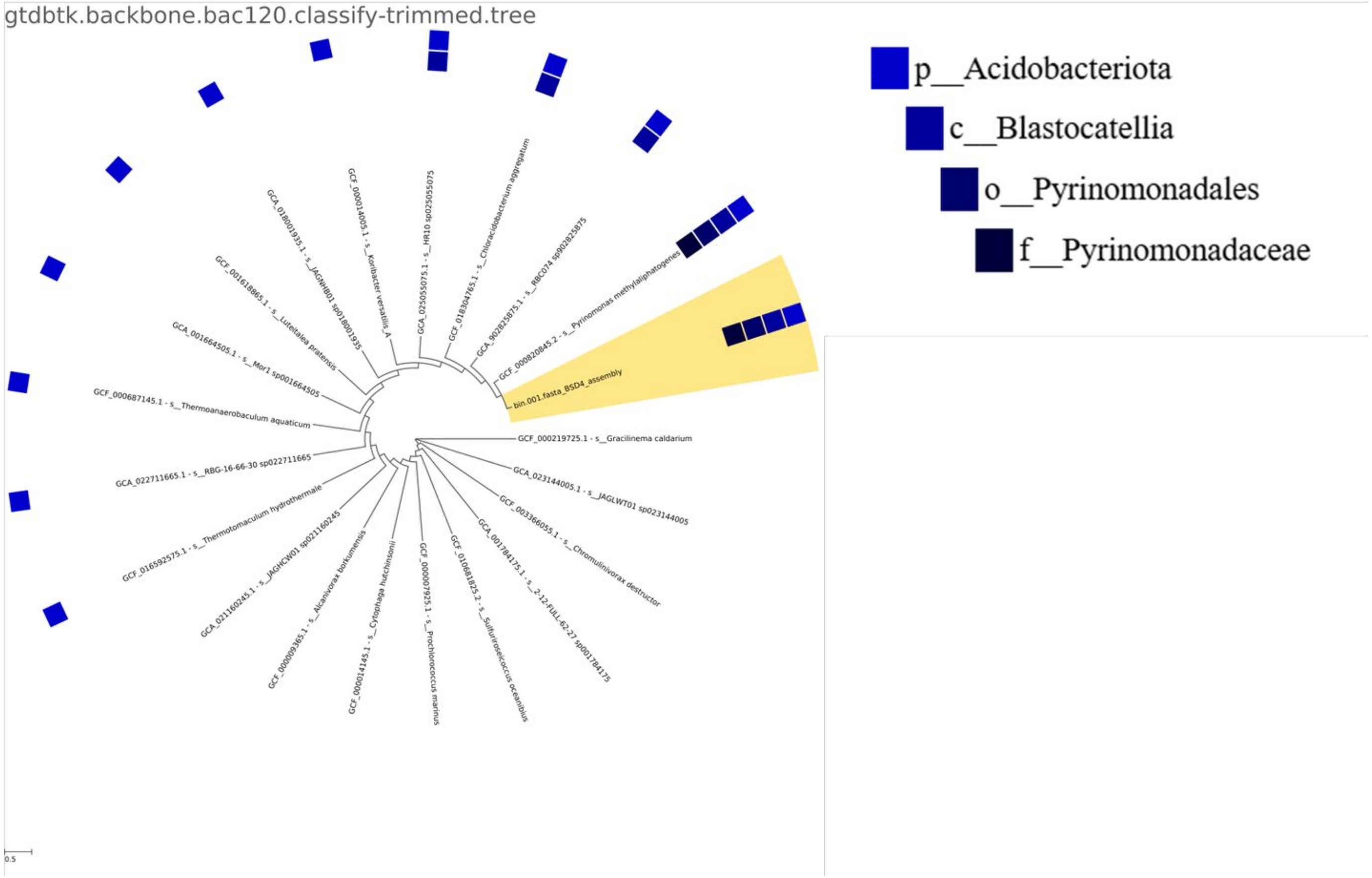

# _Comparison with BDS4 co-assembly_

**BDS4_co-assembly**

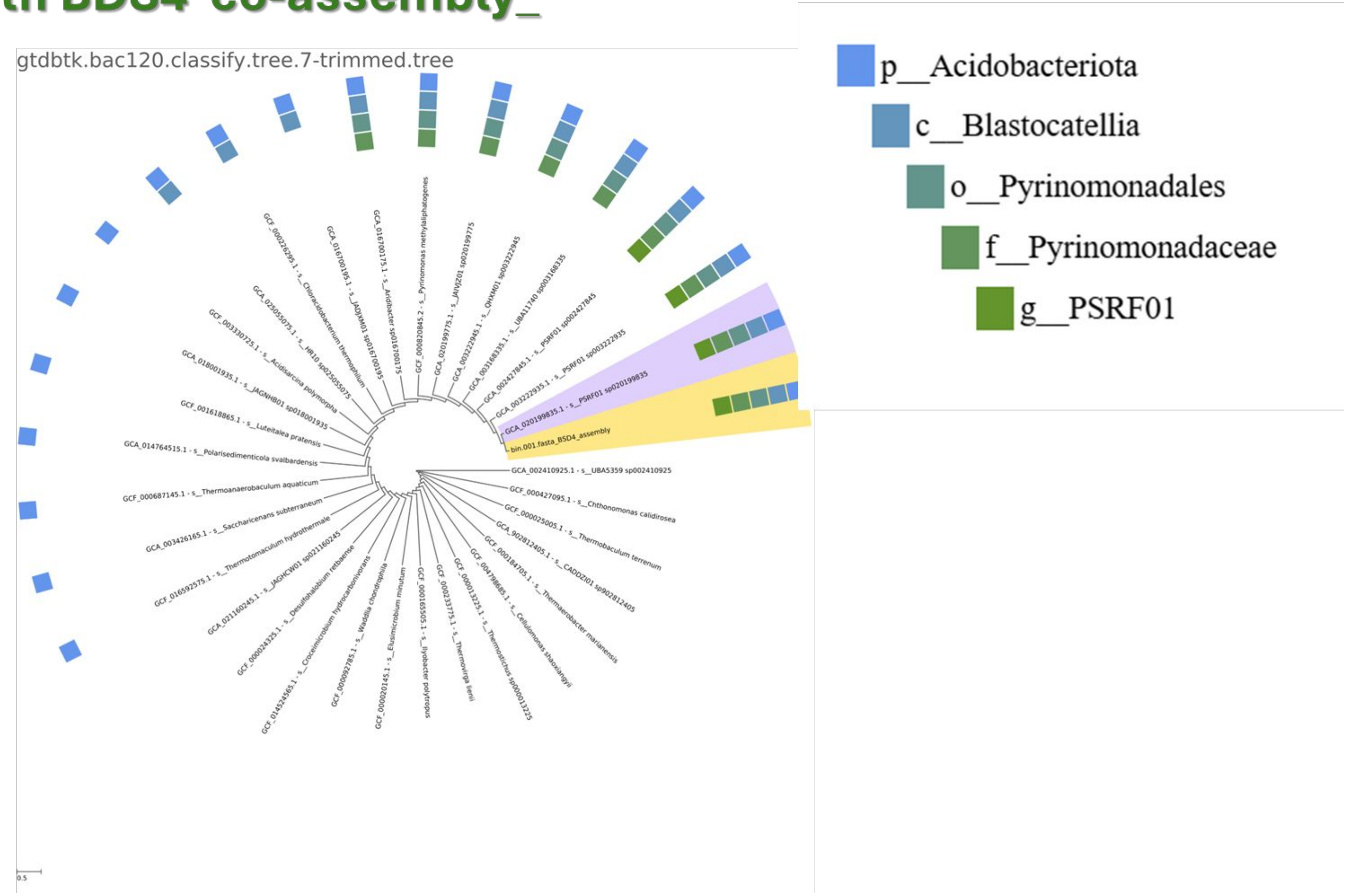

# _ Comparison with BDS4 co-assembly _

**BDS4_211B**

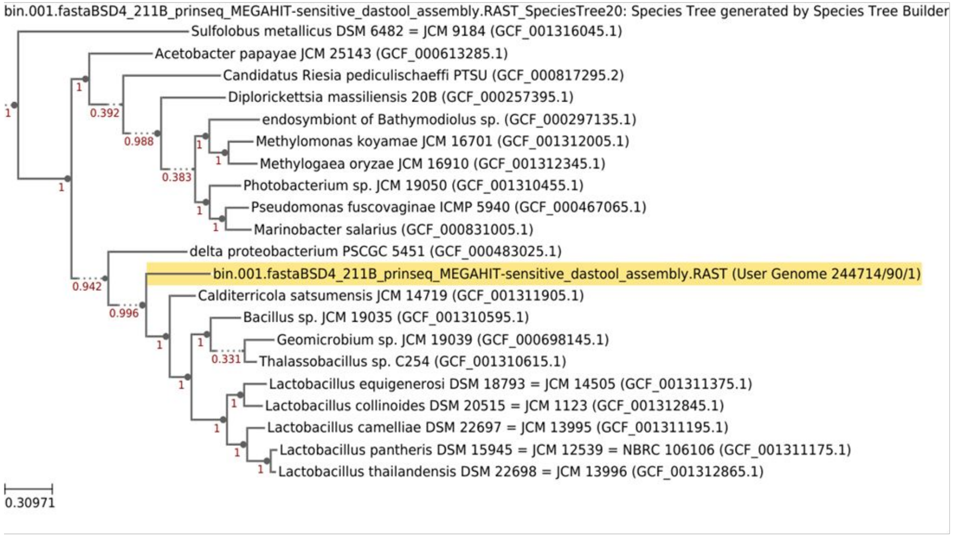


**BDS4_co-assembly**

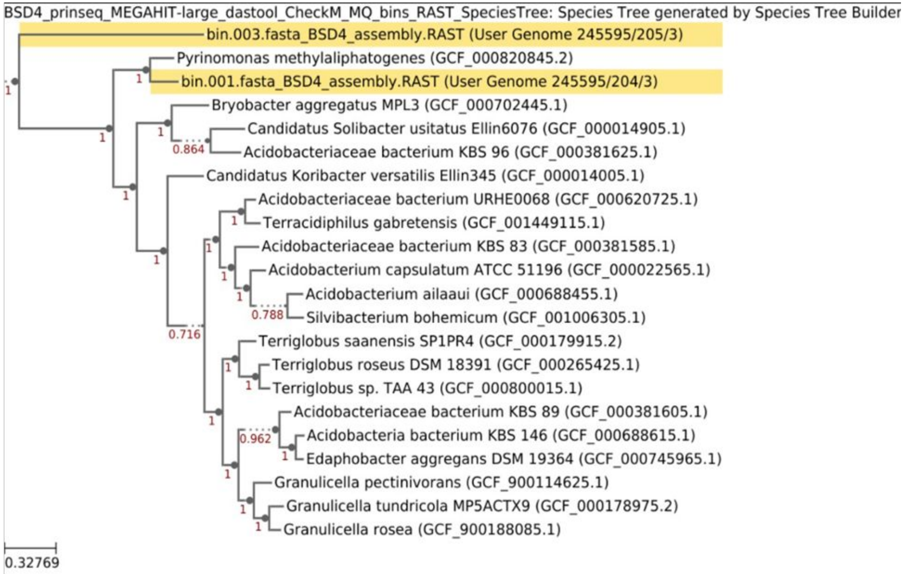


**Therefore also the phylogenetic tree does not correspond to ours.**

## _ Conclusion _

1 **BIN**: completeness 16.31%; contamination 2.44%

1 **bacterium**: Actinomycetia (class)

**Metabolic function**:

- ATP asi
- CAZ y enzime

**Genes**:

- nirD (nitrogen)
- pqqC (C1 Compounds)
- cydA (Oxygen)
- 3 hydrogenase

**Nearest species**:
*Microbacterium sp.*

# _ Conclusion _

1 **BIN**: completeness 16.31%; contamination 2.44%

1 **bacterium**: Actinomycetia (class)

**Metabolic function**:
- ATP asi
- CAZ y enzime

**Genes**:
- nirD (nitrogen)
- pqqC (C1 Compounds)
- cydA (Oxygen)
- 3 hydrogenase

**Nearest species**: *Microbacterium sp.*

The BIN does not respect the completeness cryteria, therefore all the analyses are not reliable

**Future prospective:**
repeat the sequencing and add others replicate to construct a stronger co-assembly.

The soil samples are very rich in material, so this could confuse the algorythm.

# _ Thanks for the attention _



Matilde Marzucchi
Yodit Bacchi

# The story of BSD2-199C By KBosses

- Edition number: **2**
- Location: **Vivaio Bicocca - Open Air Classroom**
- Sample type: **Soil**

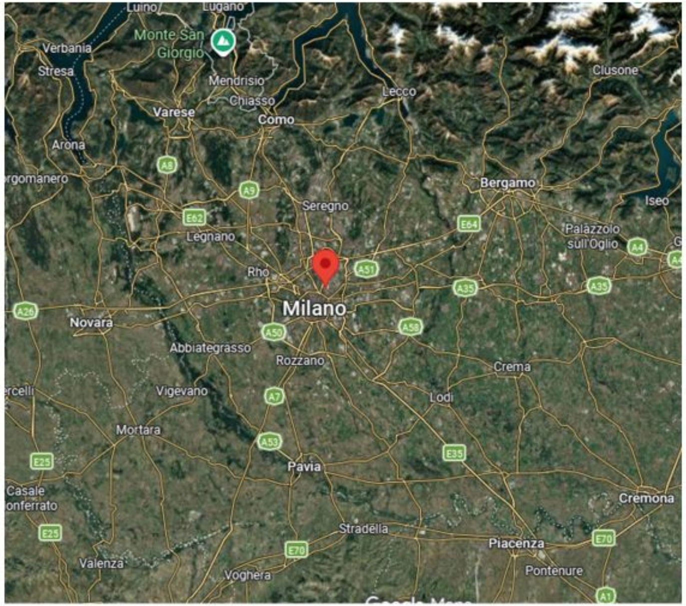


45.50801 9.20993 - 29/9/23 - 14:49

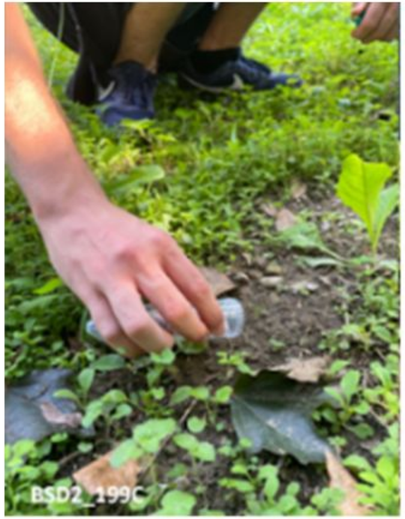

# QUALITY CONTROL OF READS

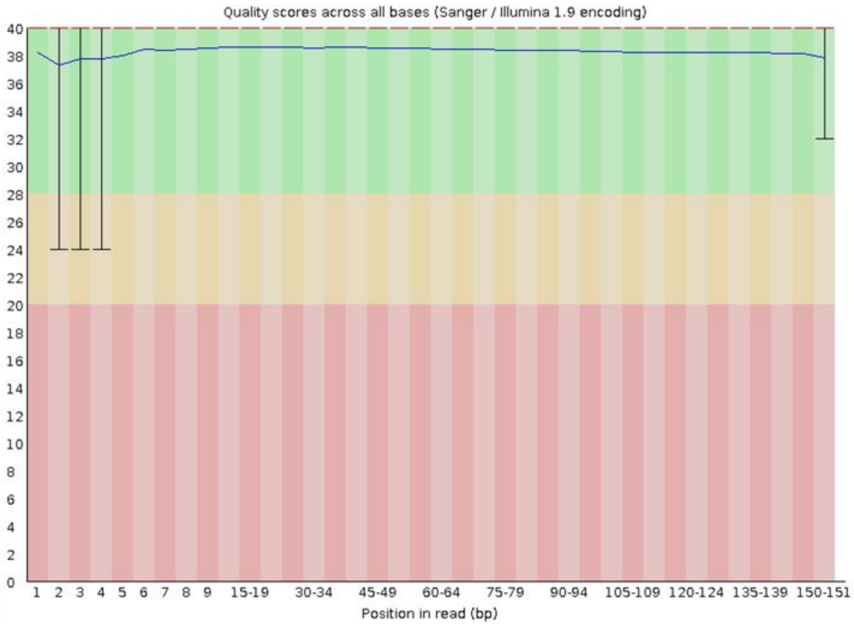


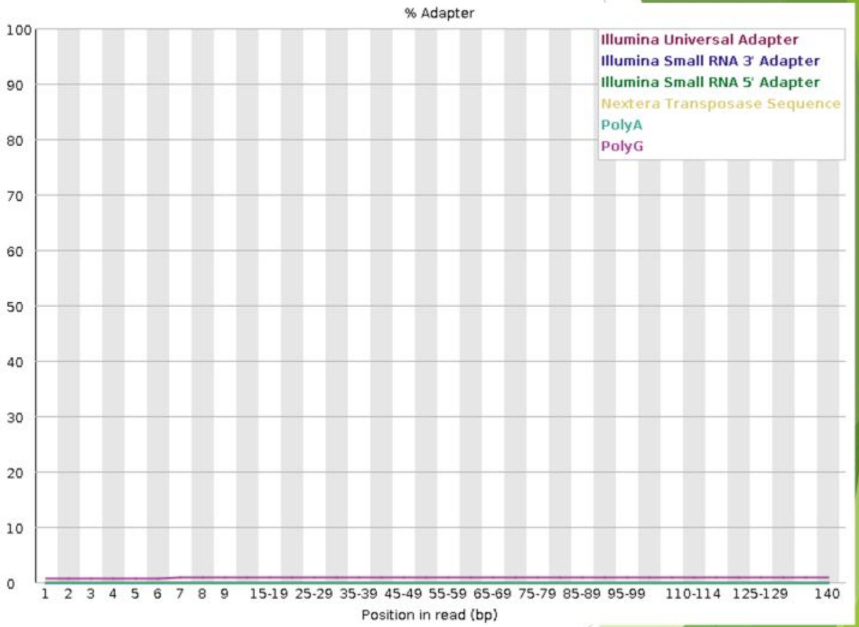


## Overrepresented sequences

| Sequence | Count | Percentage | Possible Source |
|---|---|---|---|
| GGGGGGGGGGGGGGGGGGGGGGGGGGGGGGGGGGGGGGGGGGGGGGGGGG | 296370 | 0.7940096889168256 | No Hit |

*Tool: FastQC*

# READ TAXONOMY

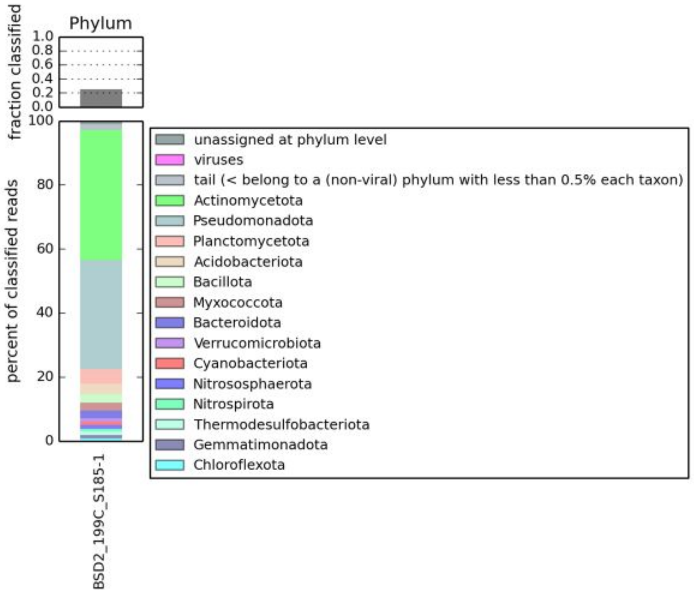


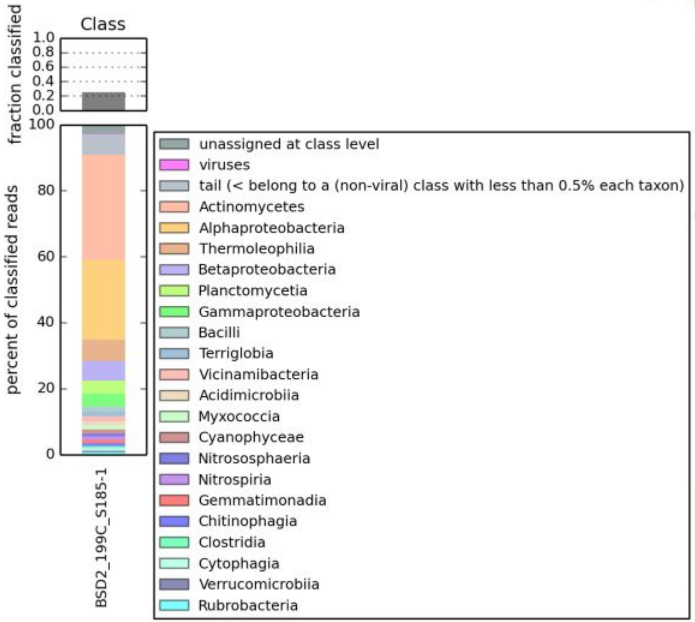


Mainly:

- **Actinomycetes** (Phylum Actinomycetota)
- **Alphaproteobacteria** (Phylum Pseudomonadota)

*Tool: KAIJU*

# QUALITY CONTROL OF ASSEMBLY

| Statistics without reference | BSD2_199C_MEGAHIT_sensitive | BSD2_199C_MEGAHIT_large | BSD2_199C_metaSPAdes | BSD2_199C_IDBA |
|---|---|---|---|---|
| # contigs | 4269 | 4390 | 3935 | 1073 |
| # contigs (>= 0 bp) | 4269 | 4390 | 3935 | 1073 |
| # contigs (>= 1000 bp) | 4269 | 4390 | 3935 | 1073 |
| # contigs (>= 10000 bp) | 8 | 6 | 12 | 5 |
| # contigs (>= 100000 bp) | 0 | 0 | 0 | 0 |
| # contigs (>= 1000000 bp) | 0 | 0 | 0 | 0 |
| Largest contig | 39 103 | 39 103 | 36 821 | 23 416 |
| Total length | 11 313 254 | 11 699 572 | 10 802 544 | 3 181 684 |
| Total length (>= 0 bp) | 11 313 254 | 11 699 572 | 10 802 544 | 3 181 684 |
| Total length (>= 1000 bp) | 11 313 254 | 11 699 572 | 10 802 544 | 3 181 684 |
| Total length (>= 10000 bp) | 123 603 | 94 352 | 163 430 | 75 025 |
| Total length (>= 100000 bp) | 0 | 0 | 0 | 0 |
| Total length (>= 1000000 bp) | 0 | 0 | 0 | 0 |
| N50 | 2534 | 2551 | 2616 | 2829 |
| N75 | 2209 | 2217 | 2245 | 2383 |
| L50 | 1716 | 1761 | 1541 | 406 |
| L75 | 2920 | 2997 | 2660 | 715 |
| GC (%) | 58.49 | 59.77 | 58.91 | 53.97 |
| **Mismatches** | | | | |
| # N's | 0 | 0 | 4560 | 0 |
| # N's per 100 kbp | 0 | 0 | 42.21 | 0 |

Chosen assembly = **metaSPAdes**, based on:

- Low number of contigs
- High lenght of the longest contig
- High total lenght of the assembly
- High N50
- Low L50

***Tool: QUAST***

# QUALITY CONTROL OF BINNING

| Bin Name | Marker Lineage | # Genomes | # Markers | # Marker Sets | 0 | 1 | 2 | 3 | 4 | 5+ | Completeness | Contamination |
|---|---|---|---|---|---|---|---|---|---|---|---|---|
| bin.001 | k__Bacteria | 88 | 230 | 148 | 141 | 72 | 15 | 1 | 1 | 0 | 43.36 | 9.8 |
| bin.002 | o__Actinomycetales | 268 | 395 | 220 | 129 | 191 | 62 | 9 | 3 | 1 | 68.36 | 24.49 |
| bin.003 | k__Archaea | 207 | 145 | 103 | 78 | 57 | 10 | 0 | 0 | 0 | 47.36 | 4.99 |
| bin.004 | k__Archaea | 207 | 145 | 103 | 83 | 62 | 0 | 0 | 0 | 0 | 44.8 | 0.0 |

Chosen Bin = **bin.004**, based on:

- Completeness
- Contamination

***Tool: CheckM***

# FUNCTIONAL ANNOTATION

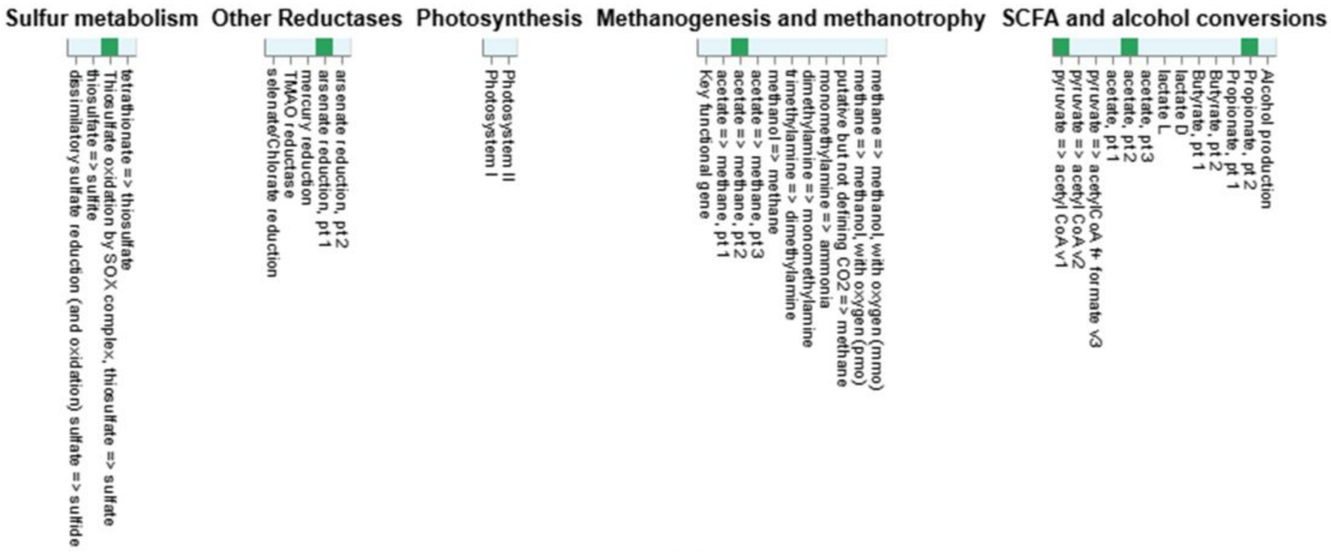


Metabolic functional capacities:

- Thiosulfate oxidation by SOX complex
- Arsenate reduction
- Methanogenesis from acetate
- Pyruvate conversion into Acetyl-CoA
- Acetate production
- Propionate production

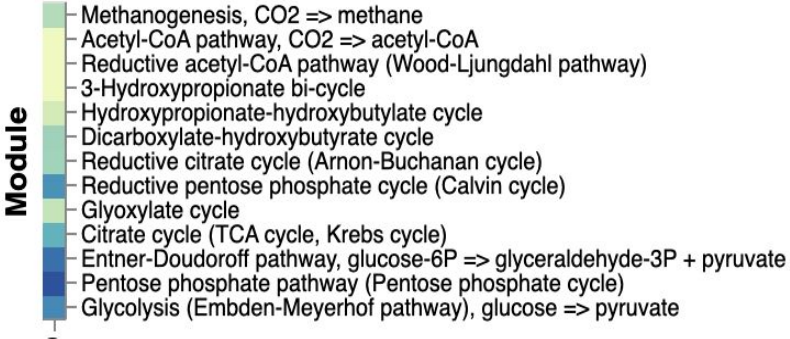


*Tool: DRAM*

# TAXONOMIC CLASSIFICATION

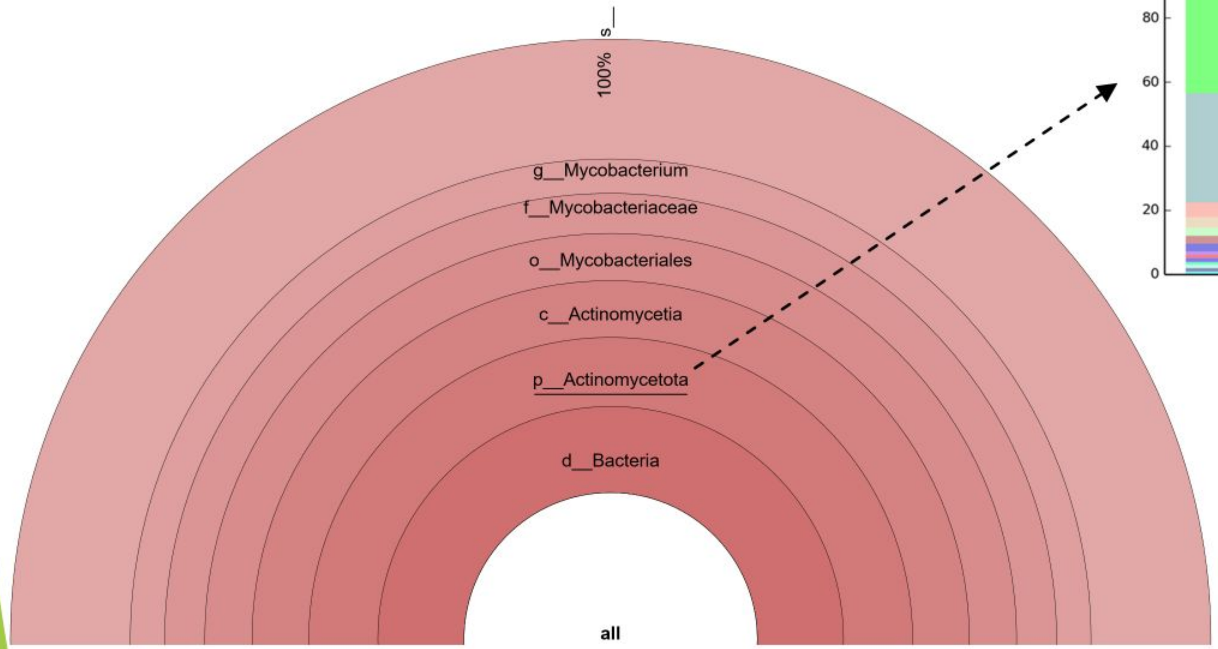


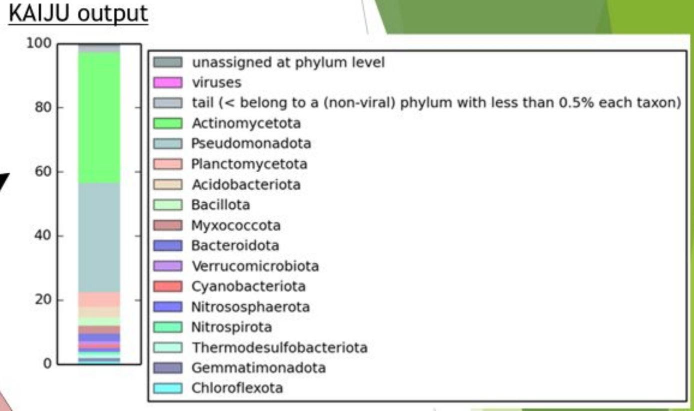


Closest placement taxonomy:
d__Bacteria
p__Actinomycetota
c__Actinomycetia
o__Mycobacteriales
f__Mycobacteriaceae
g__Mycobacterium
**s__Mycobacterium moriokaense_A**

***Tool: GTDB***

# PHYLOGENETIC TREE

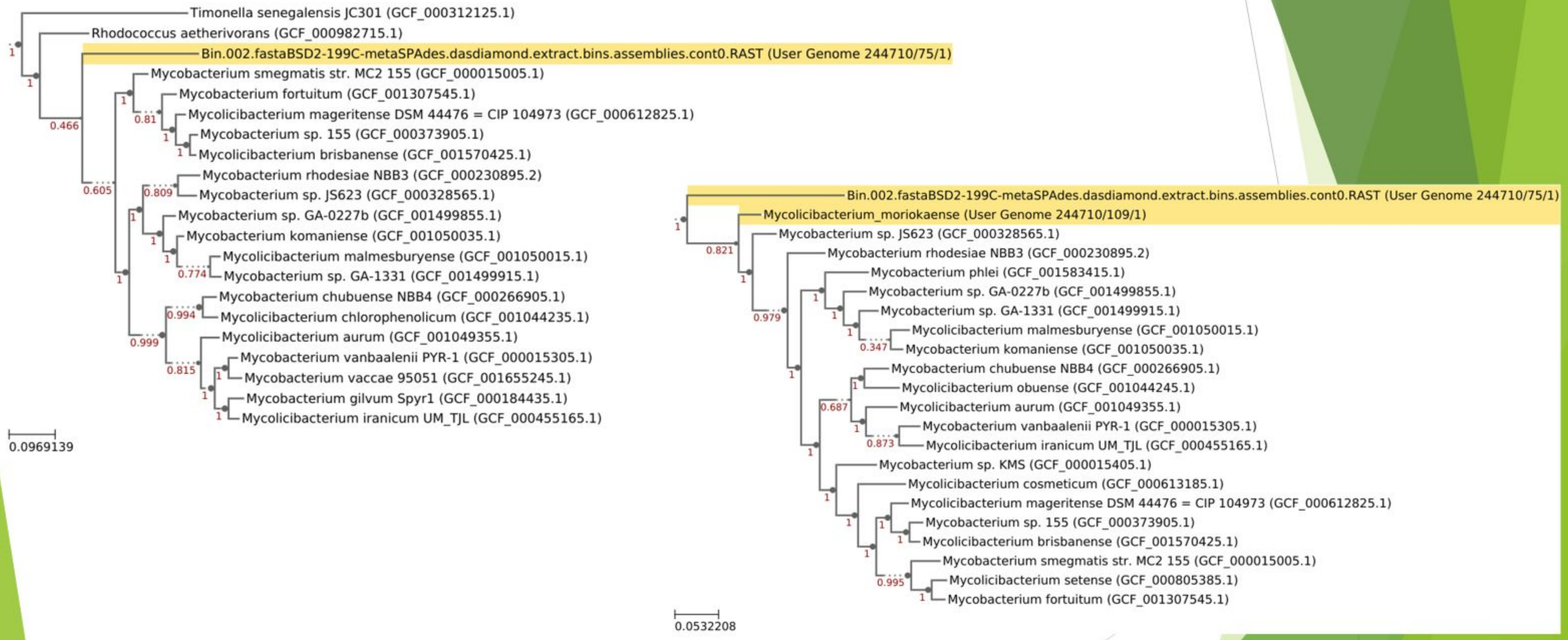


*Tool: SpeciesTree*

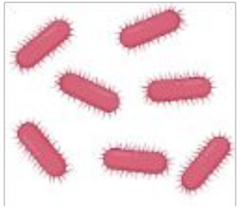

# Thank you for the attention!

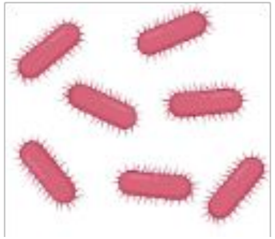

# BSD4_211C

# *What we understood so far :)*

Last but not Least

Emanuele and Alessia

| | | | | | | | | |
|---|---|---|---|---|---|---|---|---|
| BSD4 | 211C | BSD4_vivaio-bicocca_OpenAirClassroomSoil-1_r3 | vivaio-bicocca | soil | 45.50821 | 9.210036 | 27/3/24 | 14:48 |

1. Input: reads as fastq → BSD4_211C

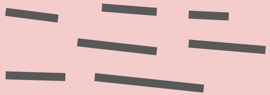

## Overrepresented sequences

| Sequence | Count | Percentage | Possible Source |
|---|---|---|---|
| GGGGGGGGGGGGGGGGGGGGGGGGGGGGGGGGGGGGGGGGGGGGGGGGGG | 421566 | 1.411618112038879 | No Hit |

# Analyses based on the QUANTITY of the reads

# *KAIJU*

Taxonomy prediction based on protein similarity on a subset of the reads → from phylum to genus.

We tried to amplify the subset percentage from 10 to 25, in order to find a match with the GTDB results …

didn't go well

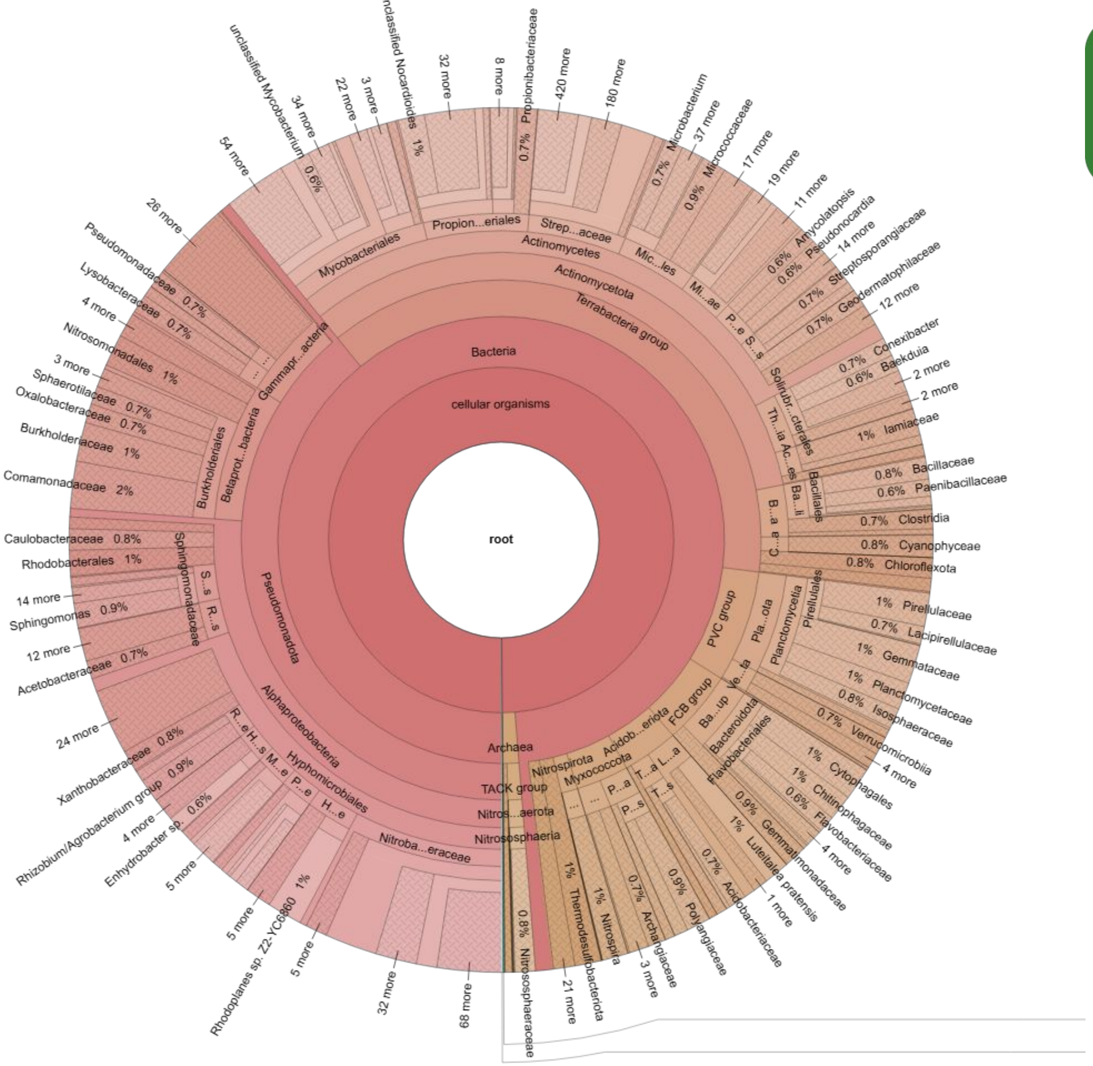

# FAMA

Functionality prediction based on a subset of the reads.

We made the tool run two times, (one searching for Nitrogen cycle genes, one for Universal single-copy marker genes), in order to find a match with DRAM & HMM results

Didn’t have luck neither here :(

## Fama report

Run info | BSD4_211C_S187PRINSEQ Functional profile | BSD4_211C_S187PRINSEQ Functional groups | BSD4_211C_S187PRINSEQ Taxonomy profile

| | | | NarC | NapF | NapB | NapA | cNor-C | NirB3 | NifH_AnfH_VnfH | UreA | NapD | UreB | NirA | NirT | NirD | AmoA_PmoA | AmoB_PmoB | NapC | NirB |
|---|---|---|---|---|---|---|---|---|---|---|---|---|---|---|---|---|---|---|---|
| | **Rank** | **Taxon name** | **efpkg** | **efpkg** | **efpkg** | **efpkg** | **efpkg** | **efpkg** | **efpkg** | **efpkg** | **efpkg** | **efpkg** | **efpkg** | **efpkg** | **efpkg** | **efpkg** | **efpkg** | **efpkg** | **efpkg** |
| **1** | norank | root | 4.156246e-02 | 0.113749 | 0.554455 | 0.872415 | 0.234790 | 0.007034 | 0.028677 | 2.379696 | 0.232300 | 2.463623e+00 | 1.189081e+00 | 0.009924 | 2.037351 | 0.269103 | 1.965754e-01 | 0.779588 | 2.649377 |
| **2** | norank | Unknown | | | | | | | | 0.038804 | | 4.488540e-02 | 3.637285e-03 | | | | | | |
| **3** | superkingdom | Bacteria | 4.007399e-02 | 0.113749 | 0.554455 | 0.872415 | 0.234790 | 0.007034 | 0.028677 | 2.200204 | 0.232300 | 2.243402e+00 | 1.162426e+00 | 0.009924 | 1.858940 | 0.098670 | 5.108694e-02 | 0.779588 | 2.649377 |
| **4** | phylum | Cyanobacteria | | | | | | | | | | 7.868129e-03 | 3.359564e-02 | | 0.002595 | | | | 0.008286 |
| **5** | order | Oscillatoriales | | | | | | | | | | | | | 0.002595 | | | | 0.001178 |
| **6** | family | Microcoleaceae | | | | | | | | | | | | | | | | | 0.001178 |
| **7** | genus | Microcoleus | | | | | | | | | | | | | | | | | 0.000592 |
| **8** | genus | Unclassified Microcoleaceae | | | | | | | | | | | | | | | | | 0.000586 |
| **9** | family | Unclassified Oscillatoriales | | | | | | | | | | | | | 0.002595 | | | | |
| **10** | order | Nostocales | | | | | | | | | | | 1.751590e-03 | | | | | | |
| **11** | family | Rivulariaceae | | | | | | | | | | | 8.778551e-04 | | | | | | |
| **12** | family | Hapalosiphonaceae | | | | | | | | | | | 8.737352e-04 | | | | | | |
| **13** | genus | Fischerella | | | | | | | | | | | 8.737352e-04 | | | | | | |
| **14** | family | Unclassified Nostocales | | | | | | | | | | | | | | | | | |
| **15** | order | Synechococcales | | | | | | | | | | 2.761829e-03 | | | | | | | |
| **16** | family | Synechococcaceae | | | | | | | | | | 2.761829e-03 | | | | | | | |
| **17** | genus | Synechococcus | | | | | | | | | | 2.761829e-03 | | | | | | | |

| Statistics without reference | BSD4_211C_prinseq_IDBA | BSD4_211C_prinseq_sensitive_m... | BSD4_211C_prinseq_metaSPAdes... | BSD4_211C_prinseq_large_megah... |
|---|---|---|---|---|
| # contigs | 1422 | 2745 | 2529 | 2756 |
| # contigs (>= 0 bp) | 1422 | 2745 | 2529 | 2756 |
| # contigs (>= 1000 bp) | 1422 | 2745 | 2529 | 2756 |
| # contigs (>= 10000 bp) | 115 | 238 | 247 | 239 |
| # contigs (>= 100000 bp) | 0 | 0 | 0 | 0 |
| # contigs (>= 1000000 bp) | 0 | 0 | 0 | 0 |
| Largest contig | 54 173 | 56 883 | 64 038 | 51 157 |
| Total length | 7 085 507 | 12 718 339 | 12 492 248 | 12 639 608 |
| Total length (>= 0 bp) | 7 085 507 | 12 718 339 | 12 492 248 | 12 639 608 |
| Total length (>= 1000 bp) | 7 085 507 | 12 718 339 | 12 492 248 | 12 639 608 |
| Total length (>= 10000 bp) | 1 708 861 | 4 089 741 | 4 649 764 | 3 947 810 |
| Total length (>= 100000 bp) | 0 | 0 | 0 | 0 |

| Bin Name | Marker Lineage | # Genomes | # Markers | # Marker Sets | 0 | 1 | 2 | 3 | 4 | 5+ | Completeness | Contamination |
|---|---|---|---|---|---|---|---|---|---|---|---|---|
| bin.001 | f__Streptomycetaceae | 60 | 460 | 233 | 293 | 158 | 9 | 0 | 0 | 0 | 31.49 | 1.6 |
| bin.002 | k__Bacteria | 2258 | 188 | 117 | 13 | 171 | 4 | 0 | 0 | 0 | 91.31 | 2.61 |

# Analyses based on the QUALITY of the reads (BIN)

# *DRAM*

Functionality identification based on our single BIN.

The output is a categorized heatma[illegible] darker the color means more steps present of the indicated pathway.

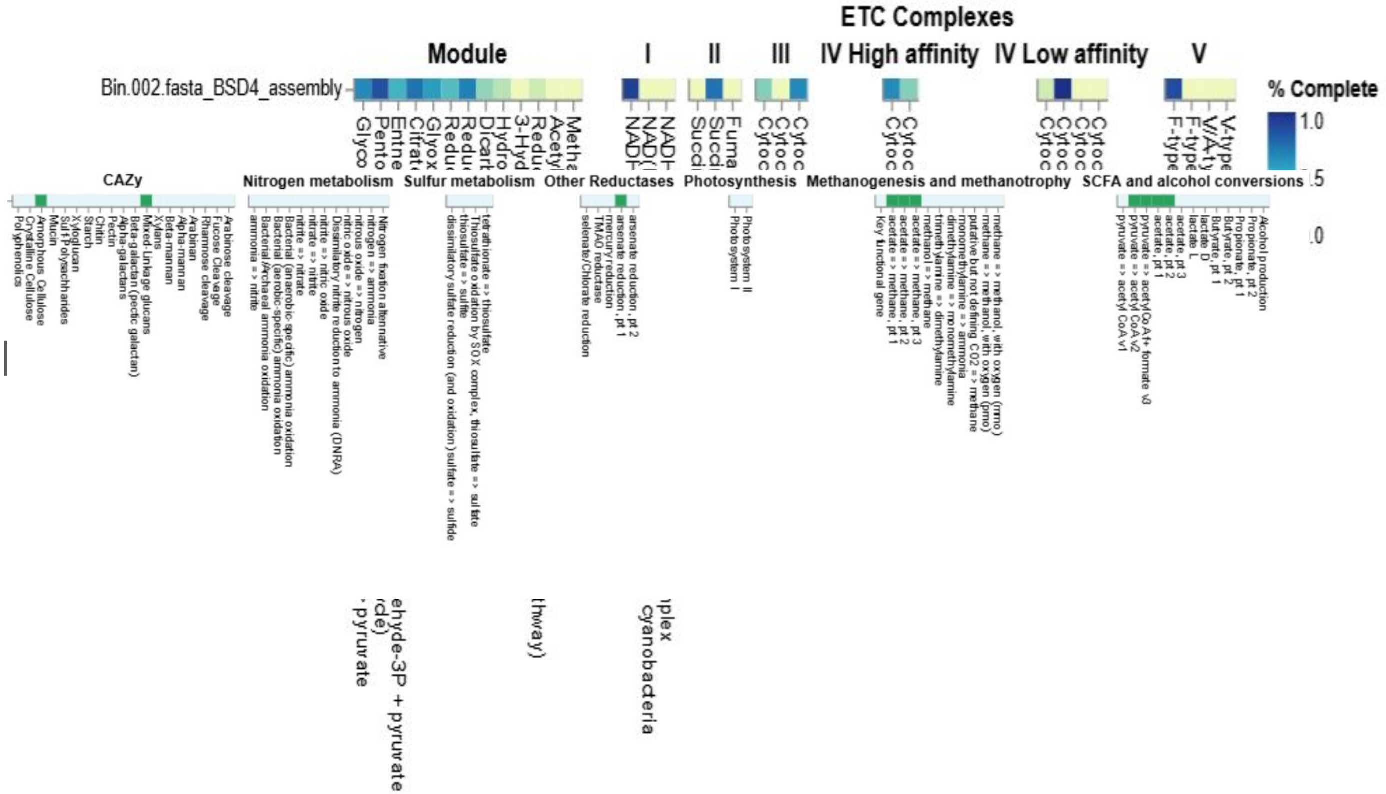

Bio Element | Phylo Marker

# *HMM*

Functionality identification based on our single BIN.

Runned both HMM Microtrait and Philogenetic Marker Tools.

The most interesting result is from the Microtrait tool → a module of an arsenic pathway

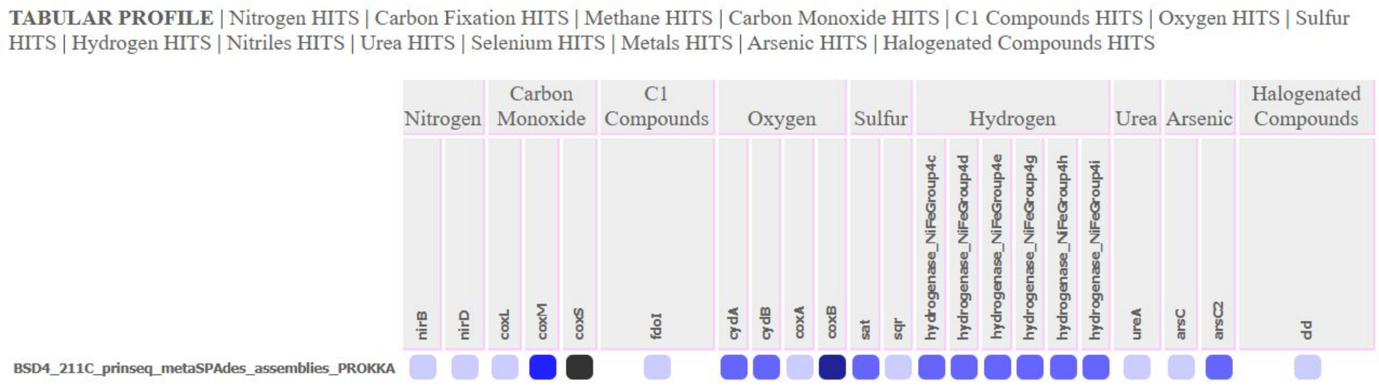


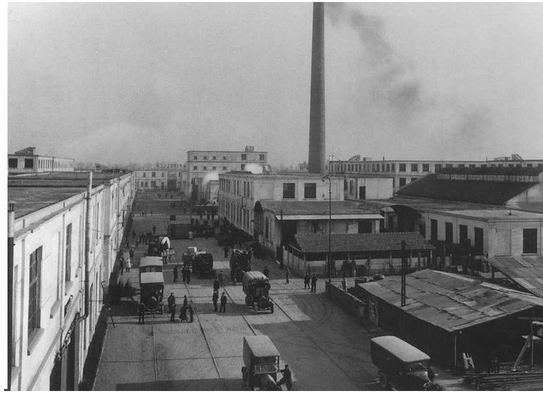

# *GTDB*

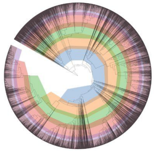

Taxonomy identification based on our single BIN.

Identified down to the genus.

Unfortunately, we couldn't make a comparison with kaiju :(

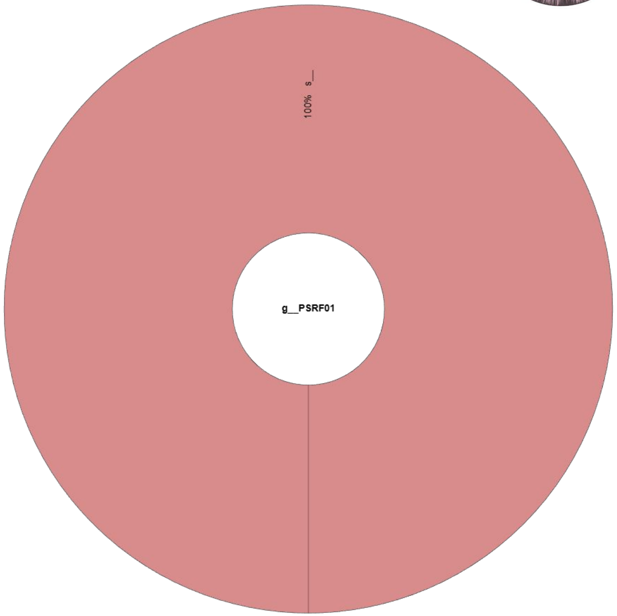


| User Genome | Classification |
|---|---|
| Bin.002.fa | d__Bacteria; p__Acidobacteriota; c__Blastocatellia; o__Pyrinomonadales; f__Pyrinomonadaceae; g__PSRF01; s__ |

| User Genome | Classification | FastANI Reference | FastANI Reference Radius | FastA Taxono | [illegible] | ...cement ...omy | Closest Placement ANI | Closest Placement Alignment Fraction | Classification Method | Note | Other Related References | MSA AA Percent | RED Value | Warnings |
|---|---|---|---|---|---|---|---|---|---|---|---|---|---|---|
| Bin.002.fa | d__Bacteria; p__Acidobacteriota; c__Blastocatellia; o__Pyrinomonadales; f__Pyrinomonadaceae; g__PSRF01; s__ | - | - | - | [illegible] | ...teriota; ...ellia; ...nadales; f__Pyrinomonadaceae; g__PSRF01; s__PSRF01 sp020199835 | 80.59 | 0.463 | taxonomic classification defined by topology and ANI | classification based on placement in class-level tree | - | 85.5 | 0.96222 | - |

# SPECIES TREE

Taxonomy identification based on our single BIN.

The tool identifies the 20 nearest neighbours to our BIN.

Because our contig was high quality, it wasn’t necessary to pair our BIN with the sequence of the closest one presente in Kbase database; therefore, we created only one tree.

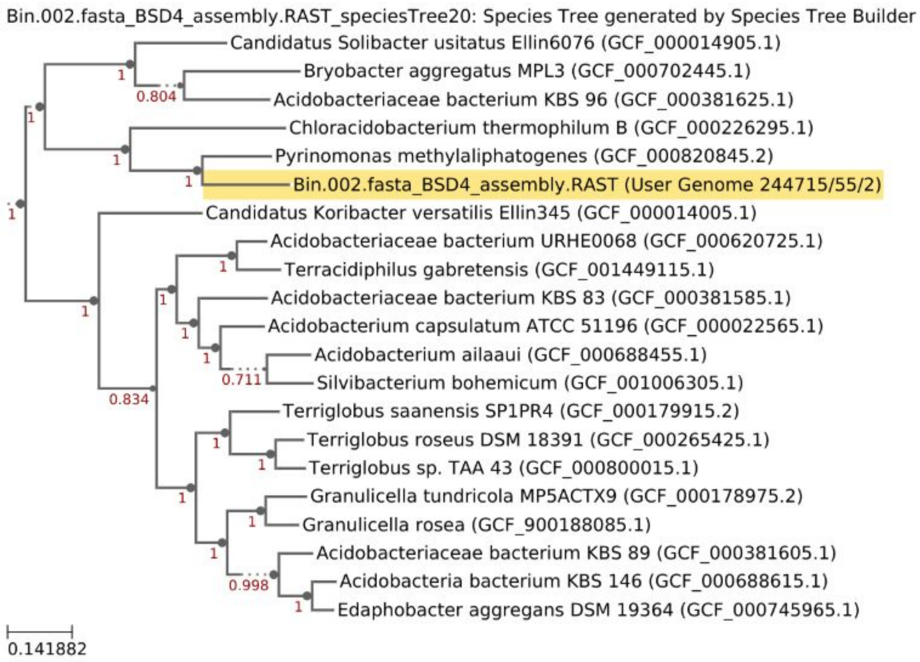

# KBase

## From Reads to Function – A hybrid workshop on metagenomics and the fantastic microbes around us

**Alessandra Caracciolo**
**Maristella Leccese**
**Polina Makarycheva**

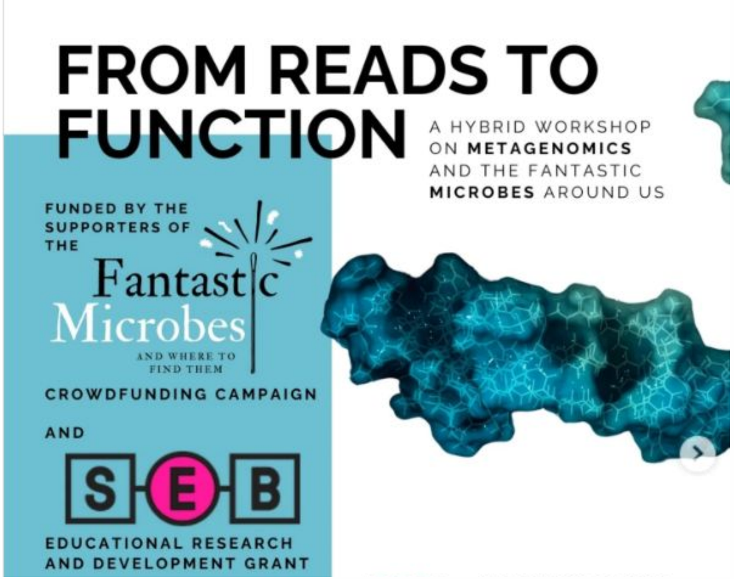

# Metadata

- **The soil samples were collected in June of 2023 in Vivaio-Bicocca**

# Whole genome analysis of soil microbiome

1. Data upload
2. Quality control
3. Read processing
4. Assembly
5. Binning
6. Bin quality control
7. Functional annotation
8. Taxonomic classification

1

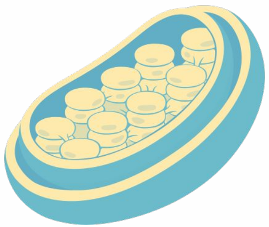

# Data Upload

We have used the BSD1_189B_S138 dataset which is a FASTA file obtained from the Illumina sequencing

2

# Quality control

**Using a FastQC tool we assessed the quality of the initial assembly.**

## Basic Statistics

| Measure | Value |
|---|---|
| Filename | BSD1_189B_S183_244711_6_1.rev.fastq |
| File type | Conventional base calls |
| Encoding | Sanger / Illumina 1.9 |
| Total Sequences | 37353536 |
| Total Bases | 5.2 Gbp |
| Sequences flagged as poor quality | 0 |
| Sequence length | 35-151 |
| %GC | 63 |

# Observations

01 **Reads for each data objects: 3735353**

02 **Initial read quality : Average ***

03 **Overrepresented sequences: Poly G**

*Quality flags: Per tile sequence quality, Per base sequence content, Per sequence GC content, Sequence length distribution

# Reads processing

- Using the **PRINSEQ** for filtering out the low-complexity reads
- Quality control with **FastQC**

## PRINSEQ

- Reads remaining after trimming: 36991733
- Reads dropped: 421024

## FastQC

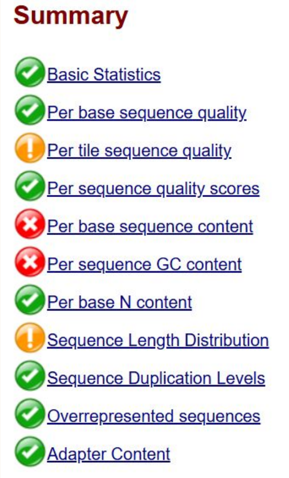

4

# Assembly

**01**

MetaSPAdes

**02**

MEGAHIT

(large)

All statistics are based on contigs of size >= 500 bp, unless otherwise noted (e.g., "# contigs (>= 0 bp)" and "Total length (>= 0 bp)" include all contigs).

Worst Median Best

Show heatmap

| Statistics without reference | BSD1_189B_S183_MEGAHIT-large | BSD1_189B_S183_metaSPAdes |
|---|---|---|
| # contigs | 2808 | 2717 |
| # contigs (>= 0 bp) | 2808 | 2717 |
| # contigs (>= 1000 bp) | 2808 | 2717 |
| # contigs (>= 10000 bp) | 335 | 356 |
| # contigs (>= 100000 bp) | 5 | 1 |
| # contigs (>= 1000000 bp) | 0 | 0 |
| Largest contig | 156 896 | 136 747 |
| Total length | 16 788 398 | 16 098 675 |
| Total length (>= 0 bp) | 16 788 398 | 16 098 675 |
| Total length (>= 1000 bp) | 16 788 398 | 16 098 675 |
| Total length (>= 10000 bp) | 8 633 011 | 7 986 046 |
| Total length (>= 100000 bp) | 618 564 | 136 747 |
| Total length (>= 1000000 bp) | 0 | 0 |
| N50 | 10 499 | 9914 |
| N75 | 3289 | 3442 |
| L50 | 312 | 363 |
| L75 | 1069 | 1078 |
| GC (%) | 52.34 | 51.97 |
| **Mismatches** | | |
| # N's | 0 | 16 910 |
| # N's per 100 kbp | 0 | 105.04 |

# Binning

- The maximum number of bins created was 23 (CONCOCT)
- Minimum number of bins created was 3 (MaxBin2)
- Six bins were created using the MetaBAT2
- Number of contigs binned by the MaxBin2 was equal to 93%

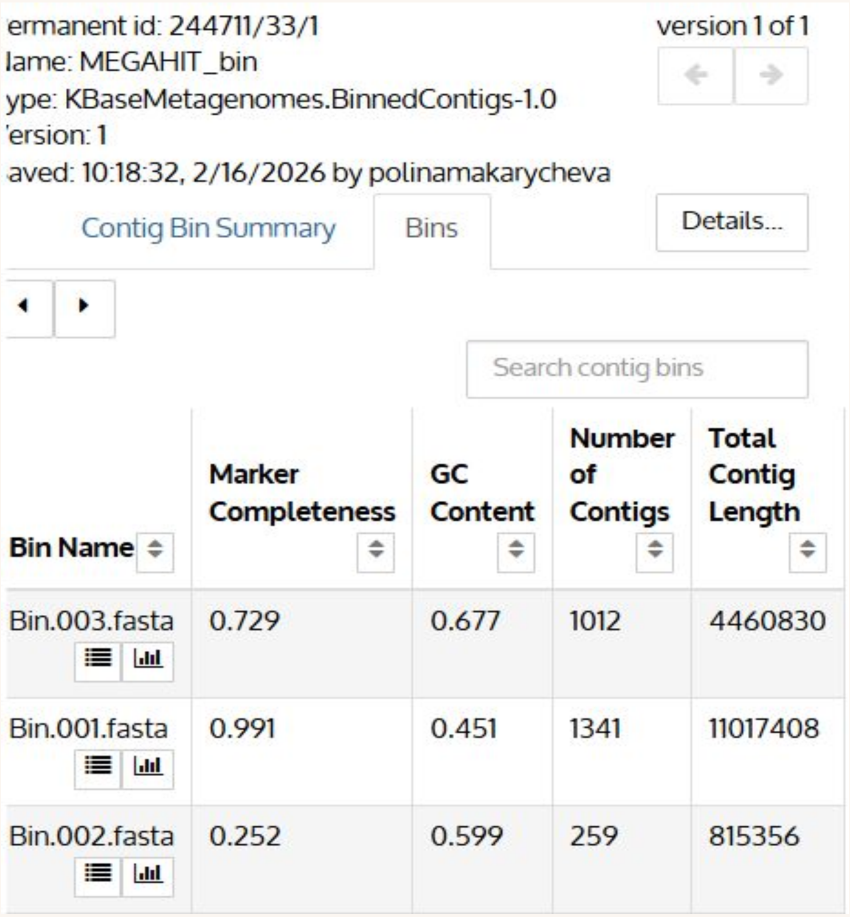
ermanent id: 244711/33/1
lame: MEGAHIT_bin
ype: KBaseMetagenomes.BinnedContigs-1.0
'ersion: 1
aved: 10:18:32, 2/16/2026 by polinamakarycheva

version 1 of 1

Contig Bin Summary | Bins | Details...

Search contig bins

| Bin Name | Marker Completeness | GC Content | Number of Contigs | Total Contig Length |
|---|---|---|---|---|
| Bin.003.fasta | 0.729 | 0.677 | 1012 | 4460830 |
| Bin.001.fasta | 0.991 | 0.451 | 1341 | 11017408 |
| Bin.002.fasta | 0.252 | 0.599 | 259 | 815356 |

6

# Binning quality control

- Bin optimization using DAS-Tool and the output of MaxBin2, Concoct, MetaBAT
- Assessment of genome quality with CheckM
- Best bin is bin2, but bin3 can also be used for further analysis
- Extract Bins as Assemblies from BinnedContigs

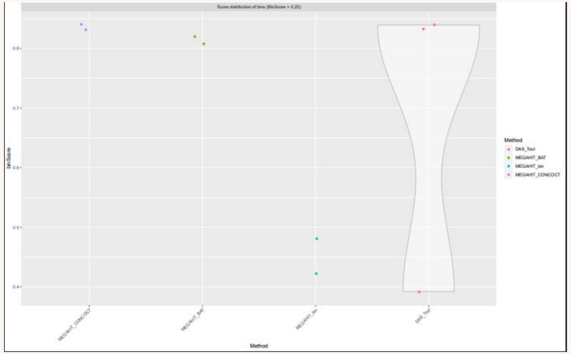

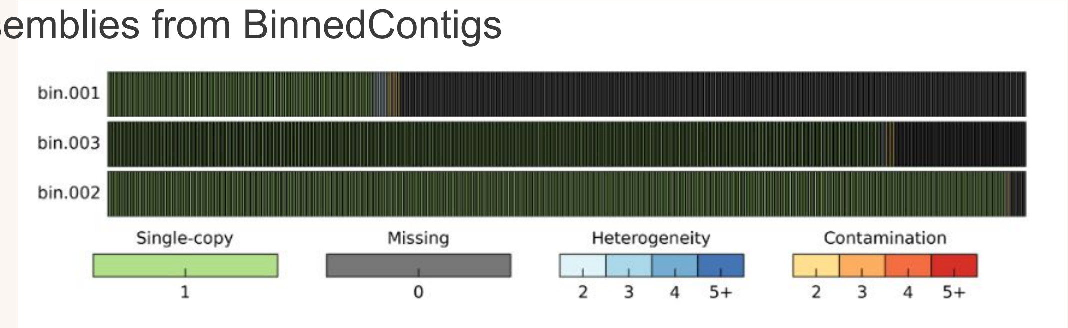


| Bin Name | Marker Lineage | # Genomes | # Markers | # Marker Sets | 0 | 1 | 2 | 3 | 4 | 5+ | Completeness | Contamination |
|---|---|---|---|---|---|---|---|---|---|---|---|---|
| bin.001 | p__Cyanobacteria | 82 | 579 | 450 | 395 | 167 | 17 | 0 | 0 | 0 | 30.38 | 2.19 |
| bin.002 | p__Cyanobacteria | 79 | 583 | 457 | 10 | 570 | 3 | 0 | 0 | 0 | 98.03 | 0.44 |
| bin.003 | f__Xanthomonadaceae | 55 | 659 | 290 | 94 | 554 | 11 | 0 | 0 | 0 | 87.21 | 2.44 |

7

# Functional annotation

- Using DRAM for bin2

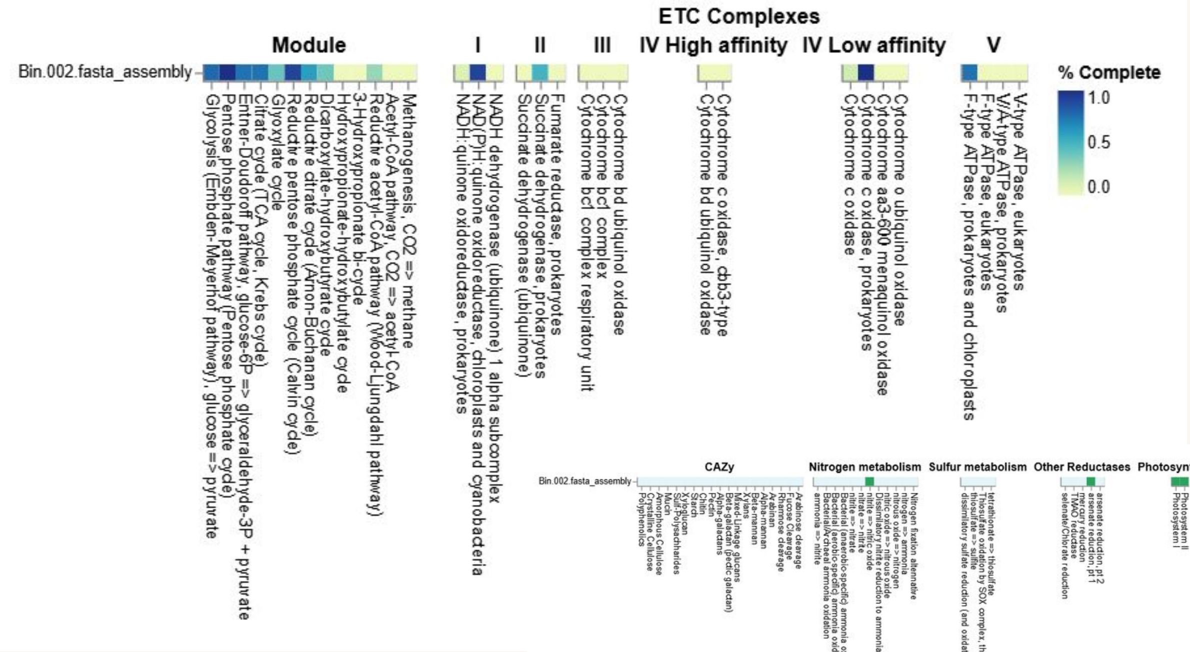


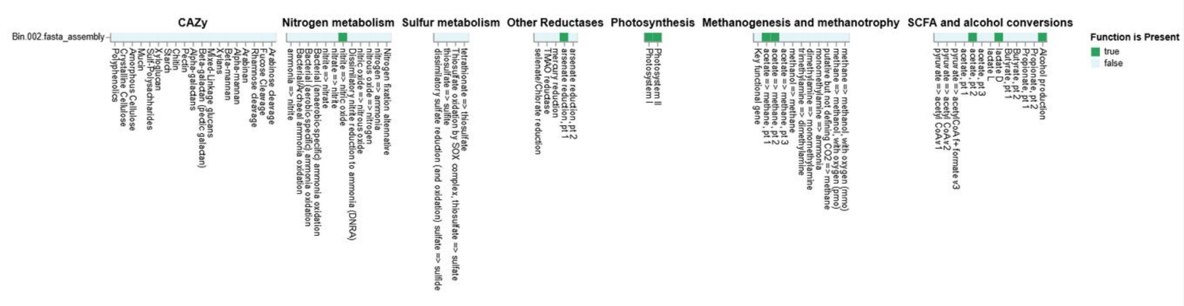


- Developed carbohydrate metabolism
- Genes responsible for the nitrogen metabolism, photosynthesis, methanogenesis and SCFA and alcohol conversions

# Taxonomic identification

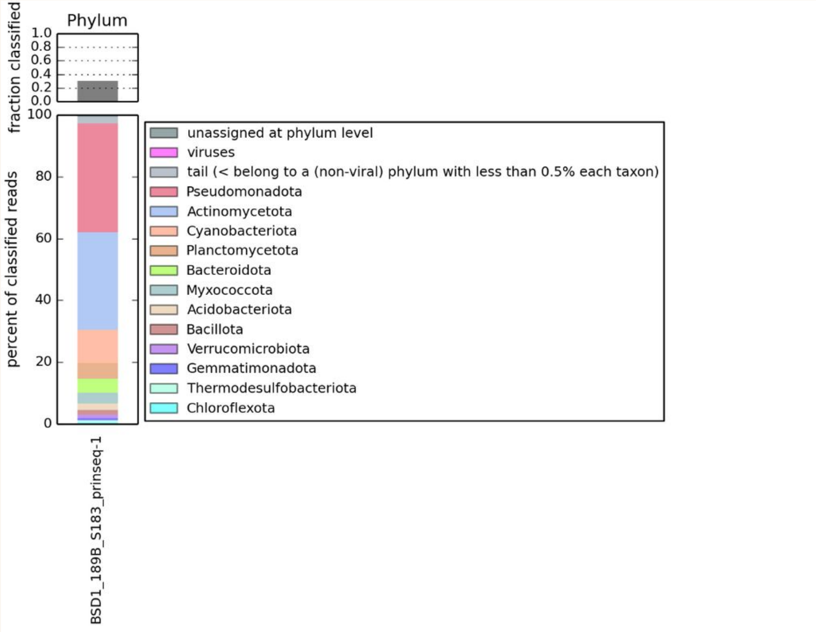


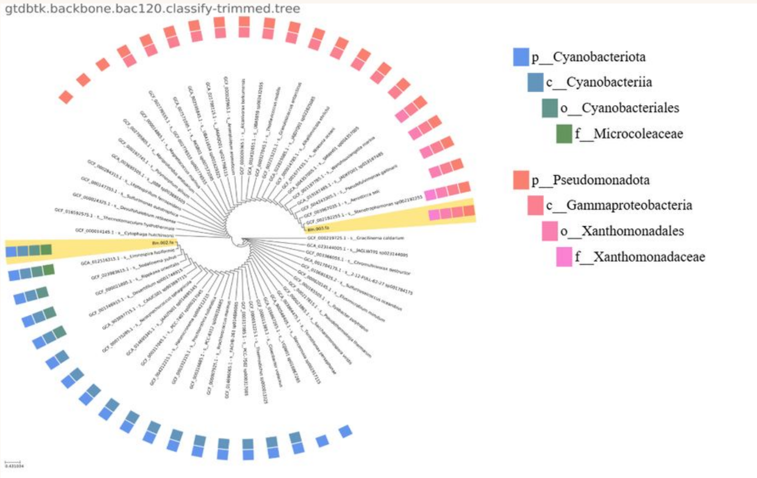


- Kaiju identifies the general phylum and species presence in the genome set. MAGs instead provide the information about the evolutionary association of the annotated genome to the other phyla of bacteria that are already present in the public repositories.
- Two phyla Pseudomonadota and Cyanobacteria are present in the dataset
- The highest unnamed taxonomy are the species, none of the MAGs have this classification.
- The longest string is Cyanobacteriota (phylum), Cyanobacteriia (class), Cyanobacteriales (order), *Microcoleaceae* (family), *Microcoleus* (genus)

7

# Functional annotation

- Prokka and RAST annotation

| | |
|---|---|
| KBase Object Name | Rast_annotetion |
| Source | User |
| Source ID | unknown |
| Size | 16,788,398 |
| GC Content | |
| Number of Features | 29550 |
| Number of Contigs | 2808 |
| Environment | |
| Number of CDSs | 14775 |
| Number of genes | 14775 |

| | |
|---|---|
| KBase Object Name | prokka_annotation |
| Source | User |
| Source ID | unknown |
| Size | 16,788,398 |
| GC Content | |
| Number of Features | 29662 |
| Number of Contigs | 2808 |
| Environment | |
| Number of CDSs | 14720 |
| Number of CRISPRs | 14 |
| Number of genes | 14720 |
| Number of rRNAs | 24 |
| Number of tRNAs | 181 |
| Number of tmRNAs | 3 |

# Taxonomic identification

- Search with dbCAN2 HMMs of CAZy families
- FAMA for nitrogen cycle genes
- Search with HMMs of Phylogenetic Marker families
- Search with HMMs of MicroTrait Bioelement families

Reference data set: nitrogen

## Input object: BSD1_189B_S183_prinseq

Total number of forward reads: 36991733

Total number of reverse reads: 36991733

Number of mapped reads, forward: 37013

Number of mapped reads, reverse: 36772

Predicted average insert size: 321

Predicted average genome size: 6899528

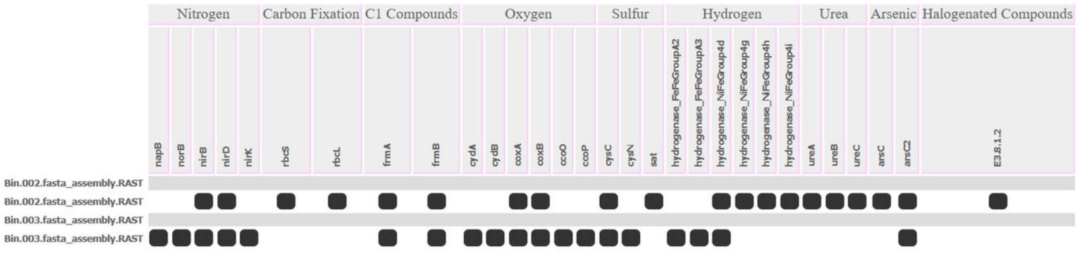

8

# Taxonomic identification

Insert Genome Into SpeciesTree

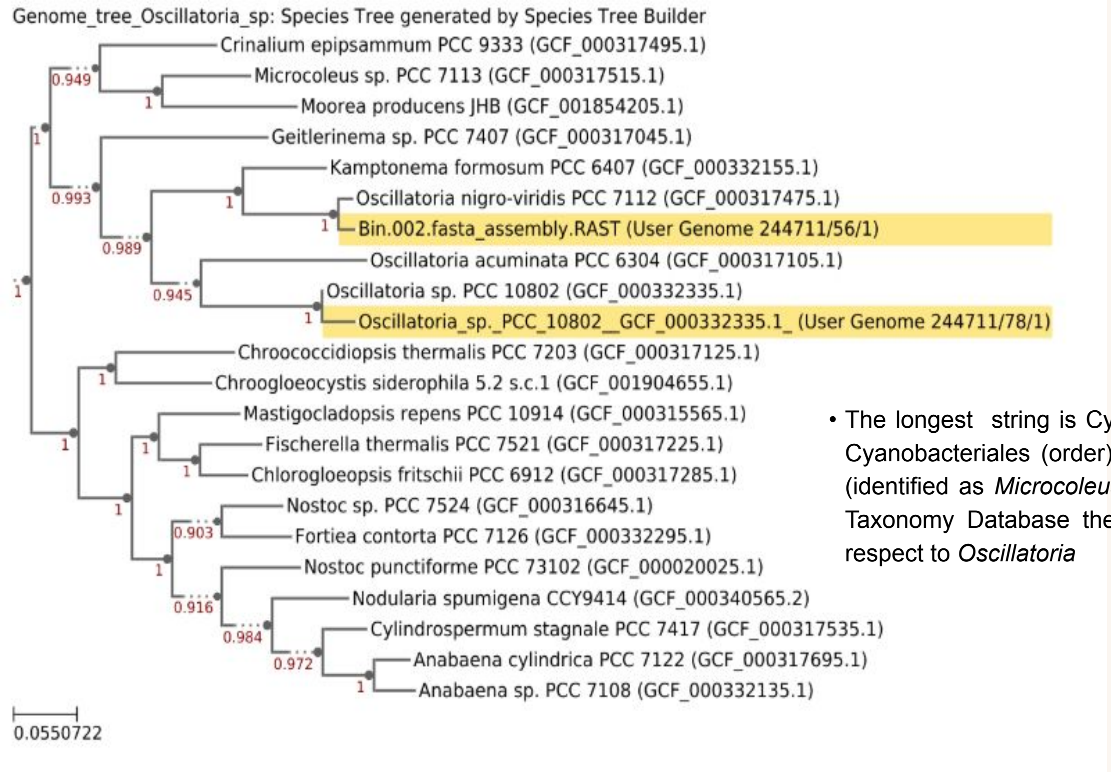


- The longest string is Cyanobacteriota (phylum), Cyanobacteriia (class), Cyanobacteriales (order), *Microcoleaceae* (family), *Microcoleus* (genus) (identified as *Microcoleus* may be due to the Fact that in The Genome Taxonomy Database there are more accessions for *Microcoleus* with respect to *Oscillatoria*

# Conclusions

- The data generally has good quality after running PRINSEQ for trimming the Poly G tale
- The genome analysed has genes responsible for the nitrogen metabolism, photosynthesis, methanogenesis and SCFA and alcohol conversions
- The genome was identified as belonging to ***Oscillatoria nigro-virdis*** species
- This species of bacteria are associated with the health of soil ecosystem. Based on the functional annotation obtained during the workshop and the bibliography search, we may hypothesise that this species of bacteria are able to convert nitrogen to ammonium which may be used by plants as a natural fertilizer*

*Chamizo S, Mugnai G, Rossi F, Certini G and De Philippis R (2018) Cyanobacteria Inoculation Improves Soil Stability and Fertility on Different Textured Soils: Gaining Insights for Applicability in Soil Restoration. Front. Environ. Sci. 6:49. doi: 10.3389/fenvs.2018.00049

# BSD1_189A

Pamela Colman, Andrea Ferrier
and Teresa Iovino

# Introduction

- Bicocca Sampling Days Project
- Sampling session: **June 22, 2023**
- Sampling area: **Vivaio Bicocca**, Piazza della Scienza, and Piazza dell'Ateneo Nuovo
- 3 kind of samples: **soil**, **water**, and **swabs** from diverse surfaces
- Illumina sequencing → raw reads

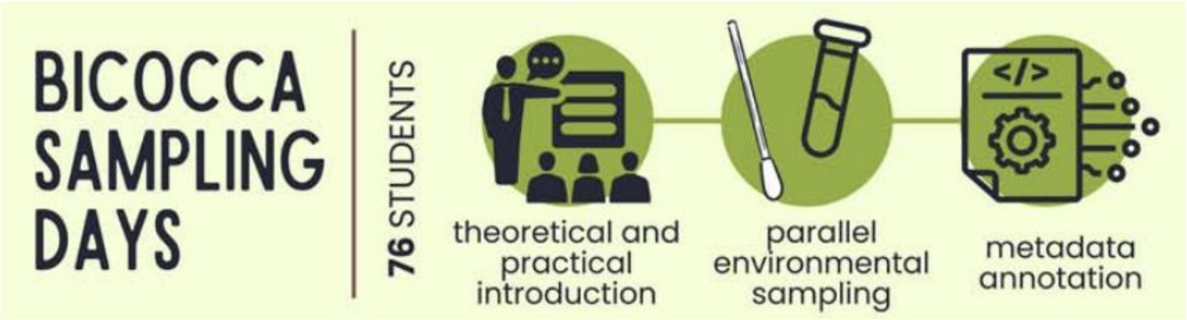

# Who is living within this community?

Poly-G overrepresented seqs on rev. (Illumina sequencing error)

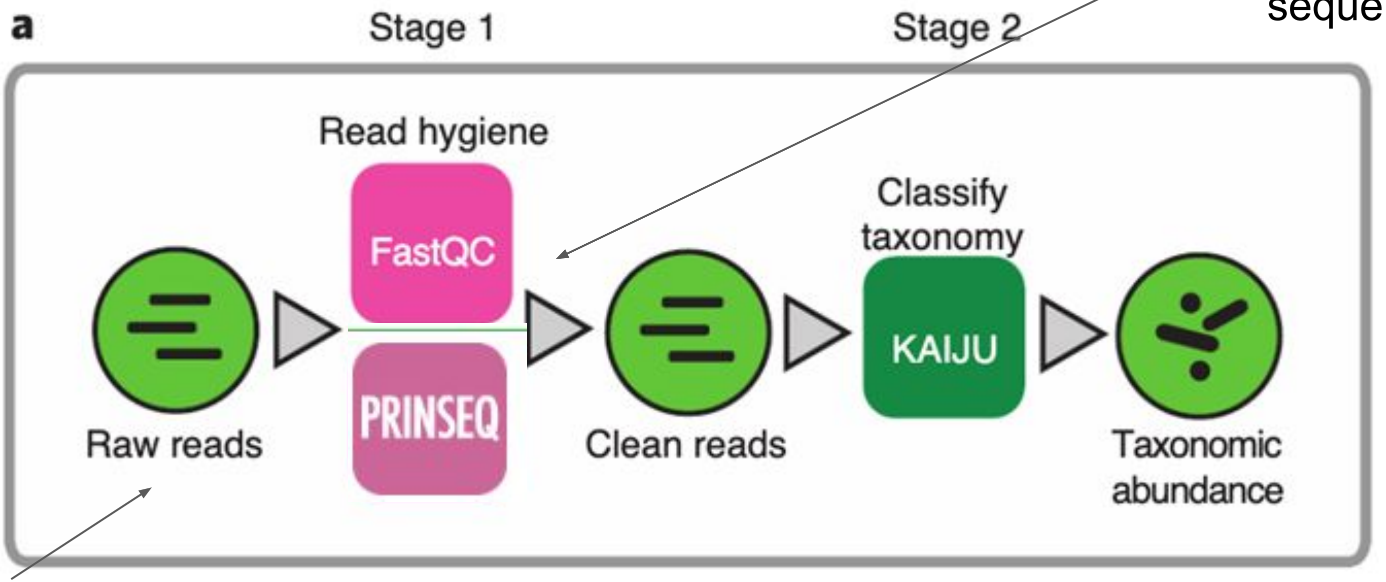


On rev and fwd there are **46931610** reads.

After trimming **46491043** reads remains

83,741 (0.18%) dropped

# How abundant are the various constituents?

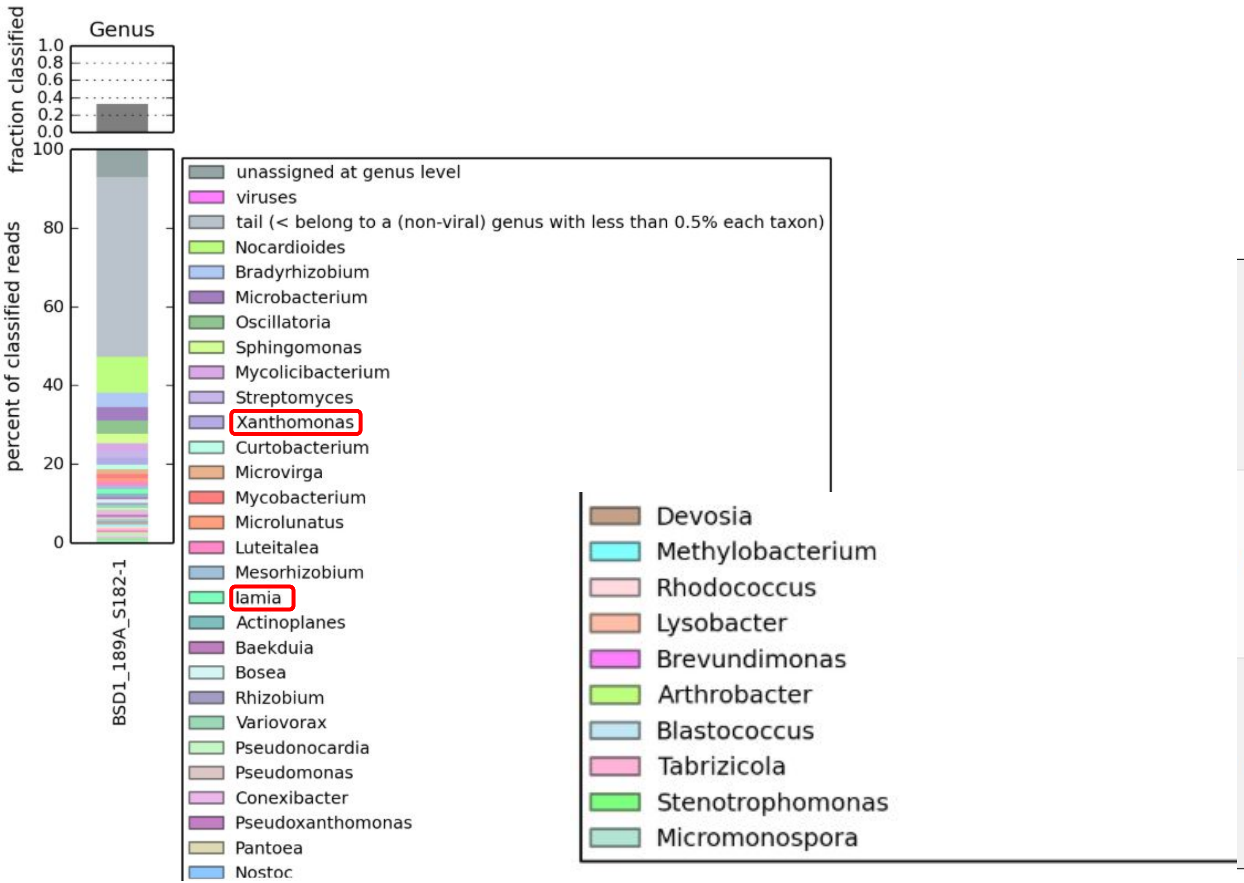


| User Genome | Classification |
|---|---|
| Bin.003.fasta_BSD1_assembly | d__Bacteria; p__Pseudomonadota; c__Gammaproteobacteria; o__Xanthomonadales; f__Xanthomonadaceae; g__Xanthomonas; s__Xanthomonas prunicola |
| Bin.004.fasta_BSD1_assembly | d__Bacteria; p__Pseudomonadota; c__Gammaproteobacteria; o__Xanthomonadales; f__Xanthomonadaceae; g__; s__ |
| Bin.005.fasta_BSD1_assembly | d__Bacteria; p__Actinomycetota; c__Acidimicrobiia; o__Acidimicrobiales; f__JAAYBP01; g__Iamia; s__ |

# What can this organism do?

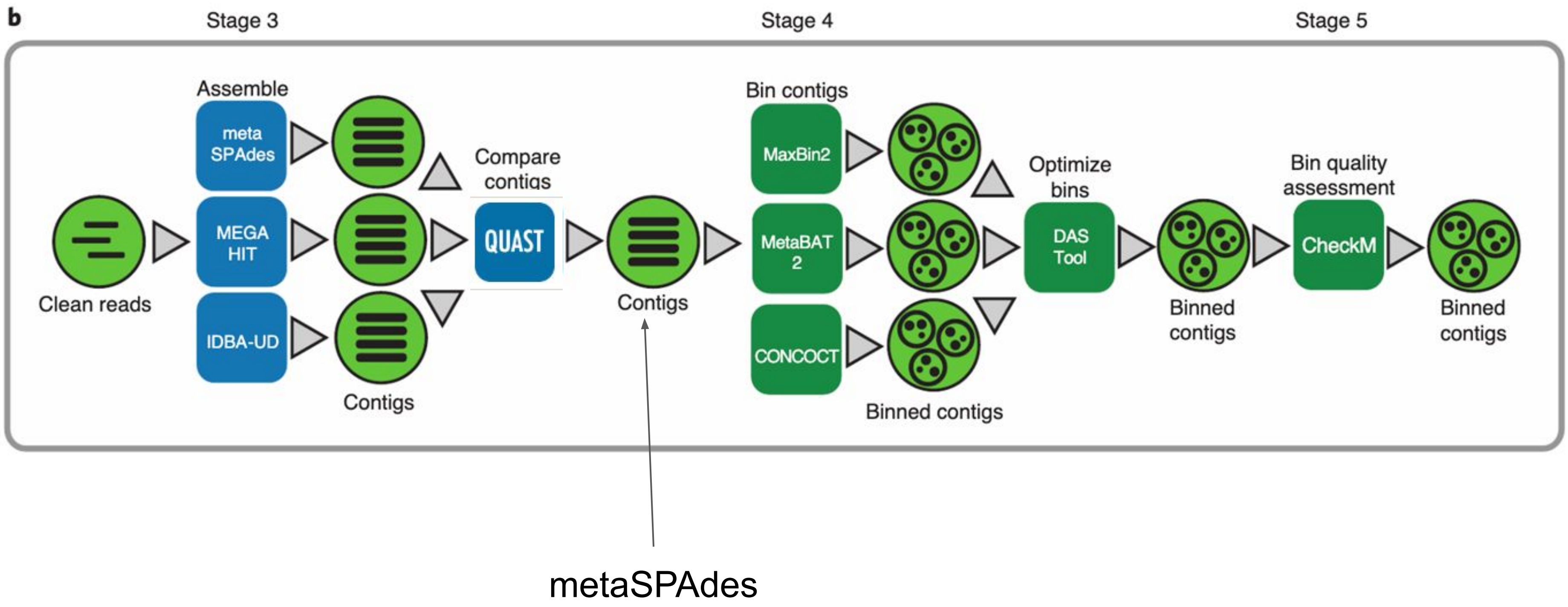

Worst Median Best

☑ Show heatmap

| Statistics without reference | BSD1_189A_prinseq_SENSITIVE_M... | BSD1_189A_prinseq_large_MEGAH... | BSD1_189A_prinseq_IDBA.contigs | BSD1_189A_prinseq_metaSPAdes.... |
|---|---|---|---|---|
| # contigs | 7561 | 8320 | 4117 | 7654 |
| # contigs (>= 0 bp) | 7561 | 8320 | 4117 | 7654 |
| # contigs (>= 1000 bp) | 7561 | 8320 | 4117 | 7654 |
| # contigs (>= 10000 bp) | 496 | 494 | 471 | 279 |
| # contigs (>= 100000 bp) | 0 | 0 | 0 | 1 |
| # contigs (>= 1000000 bp) | 0 | 0 | 0 | 0 |
| Largest contig | 63 186 | 99 834 | 82 147 | 134 817 |
| Total length | 32 732 461 | 35 934 189 | 22 235 382 | 30 568 989 |
| Total length (>= 0 bp) | 32 732 461 | 35 934 189 | 22 235 382 | 30 568 989 |
| Total length (>= 1000 bp) | 32 732 461 | 35 934 189 | 22 235 382 | 30 568 989 |
| Total length (>= 10000 bp) | 9 437 882 | 9 506 752 | 9 237 064 | 5 994 048 |
| Total length (>= 100000 bp) | 0 | 0 | 0 | 134 817 |
| Total length (>= 1000000 bp) | 0 | 0 | 0 | 0 |
| N50 | 4503 | 4485 | 7018 | 3991 |
| N75 | 2725 | 2786 | 3237 | 2684 |
| L50 | 1592 | 1860 | 697 | 1943 |
| L75 | 4008 | 4458 | 1936 | 4324 |
| GC (%) | 59.82 | 60.95 | 58.33 | 61.92 |
| **Mismatches** | | | | |
| # N's | 0 | 0 | 0 | 32 280 |
| # N's per 100 kbp | 0 | 0 | 0 | 105.6 |

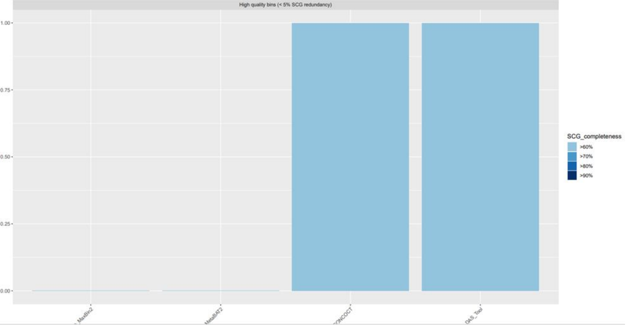


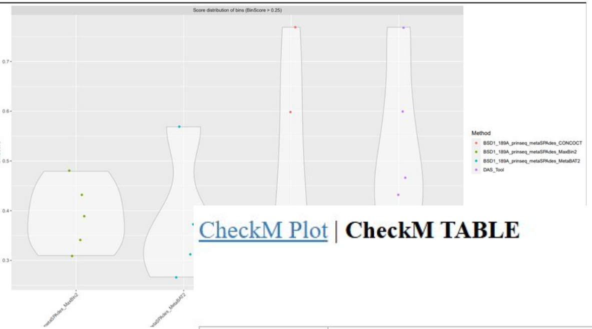


A single set of refined contig bins was generated using **DAS-Tool**

TOT 5 bins

**CheckM**: >50% completeness and <16% of contamination.

And **3 bins** survive the filters.

CheckM Plot | **CheckM TABLE**

| Bin Name | Marker Lineage | # Genomes | # Markers | # Marker Sets | 0 | 1 | 2 | 3 | 4 | 5+ | Completeness (>50%) | Contamination (<16%) |
|---|---|---|---|---|---|---|---|---|---|---|---|---|
| bin.001 | p__Cyanobacteria | 79 | 583 | 457 | 281 | 274 | 25 | 3 | 0 | 0 | 49.31 | 5.65 |
| bin.002 | p__Cyanobacteria | 129 | 472 | 368 | 402 | 69 | 1 | 0 | 0 | 0 | 12.85 | 0.14 |
| bin.003 | c__Gammaproteobacteria | 67 | 481 | 276 | 48 | 369 | 62 | 2 | 0 | 0 | 94.52 | 15.55 |
| bin.004 | f__Xanthomonadaceae | 55 | 659 | 290 | 334 | 286 | 38 | 1 | 0 | 0 | 50.82 | 5.63 |
| bin.005 | k__Bacteria | 901 | 171 | 117 | 66 | 89 | 15 | 1 | 0 | 0 | 67.44 | 14.1 |

# Are there unique functions or metabolism present?

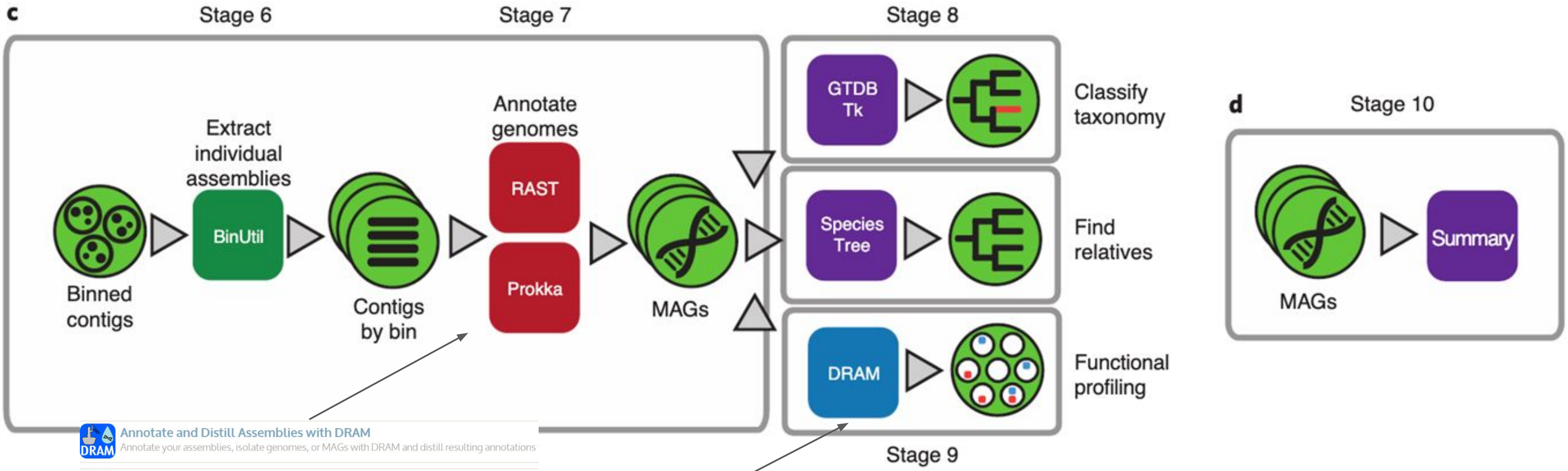


**Annotate and Distill Assemblies with DRAM**
Annotate your assemblies, isolate genomes, or MAGs with DRAM and distill resulting annotations

**Annotate Metagenome Assembly with Prokka - v1.14.5**
Annotate Metagenome Assembly with Prokka annotation pipeline.

**BSD1_189A_metaSPAdes.Assembly_Prokka**
v1 - KBaseMetagenomes.AnnotatedMetagenomeAssembly-1.0

**Annotate Multiple Microbial Assemblies with RASTtk - v1.073**
Annotate bacterial or archaeal assemblies and/or assembly sets using RASTtk (Rapid Annotations

**Annotate and Distill Genomes with DRAM**
Annotate MAGs with DRAM and distill resulting annotations to create an interactive functional sum

**Bin.003.fasta_BSD1_assembly.RAST**
v1 - KBaseGenomes.Genome-11.1

**Bin.004.fasta_BSD1_assembly.RAST**
v1 - KBaseGenomes.Genome-11.1

# Taxonomy classification

| User Genome | Classification | FastANI Reference | FastANI Reference Radius | FastANI Taxonomy | FastANI ANI | FastANI Alignment Fraction | Closest Placement Reference | Closest Placement Taxonomy | Closest Placement ANI |
|---|---|---|---|---|---|---|---|---|---|
| Bin.003.fasta_BSD1_assembly | d__Bacteria; p__Pseudomonadota; c__Gammaproteobacteria; o__Xanthomonadales; f__Xanthomonadaceae; g__Xanthomonas; s__Xanthomonas prunicola | GCF_002846205.1 | 95 | d__Bacteria; p__Pseudomonadota; c__Gammaproteobacteria; o__Xanthomonadales; f__Xanthomonadaceae; g__Xanthomonas; s__Xanthomonas prunicola | 99.28 | 0.922 | GCF_002846205.1 | d__Bacteria; p__Pseudomonadota; c__Gammaproteobacteria; o__Xanthomonadales; f__Xanthomonadaceae; g__Xanthomonas; s__Xanthomonas prunicola | 99.28 |
| Bin.004.fasta_BSD1_assembly | d__Bacteria; p__Pseudomonadota; c__Gammaproteobacteria; o__Xanthomonadales; f__Xanthomonadaceae; g__; s__ | - | - | - | - | - | - | - | - |
| Bin.005.fasta_BSD1_assembly | d__Bacteria; p__Actinomycetota; c__Acidimicrobiia; o__Acidimicrobiales; f__JAAYBP01; g__Iamia; s__ | - | - | - | - | - | GCF_019443745.1 | d__Bacteria; p__Actinomycetota; c__Acidimicrobiia; o__Acidimicrobiales; f__JAAYBP01; g__Iamia; s__Iamia sp019443745 | 83.4 |

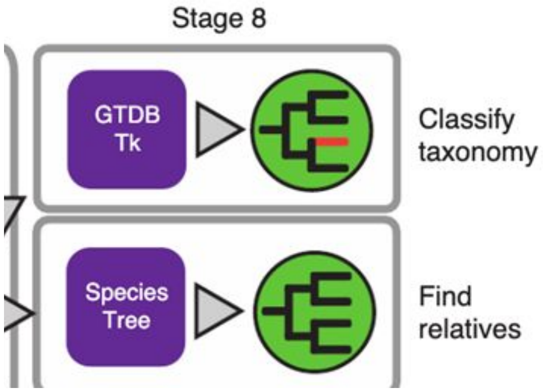


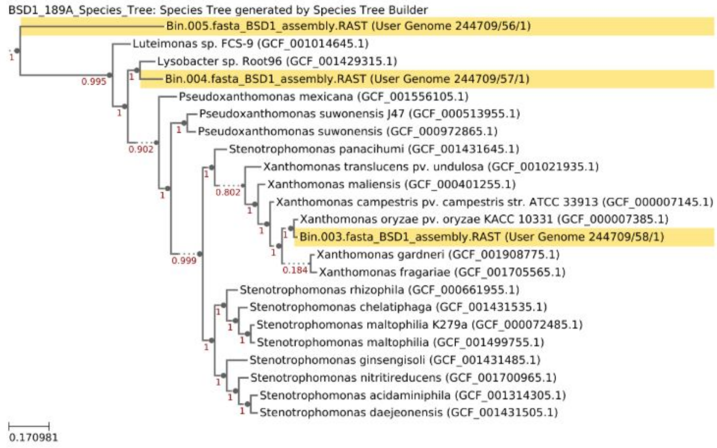


GenomeSet

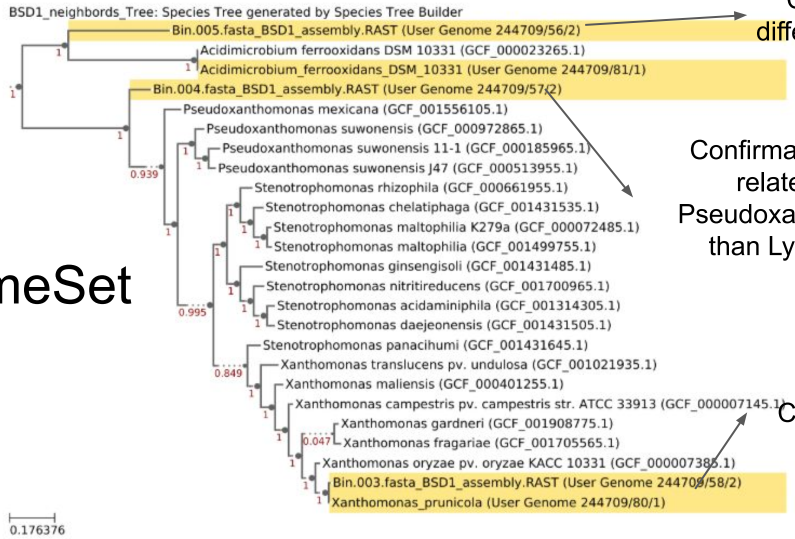


Completely different species

Confirmation: more related to a Pseudoxanthomonas than Lysobacter

Confirmation

Kaiju is important because it predicts the microbial composition based on protein similarities rather than genome assembly and annotation. This prediction can be used to compare the Species Trees against.

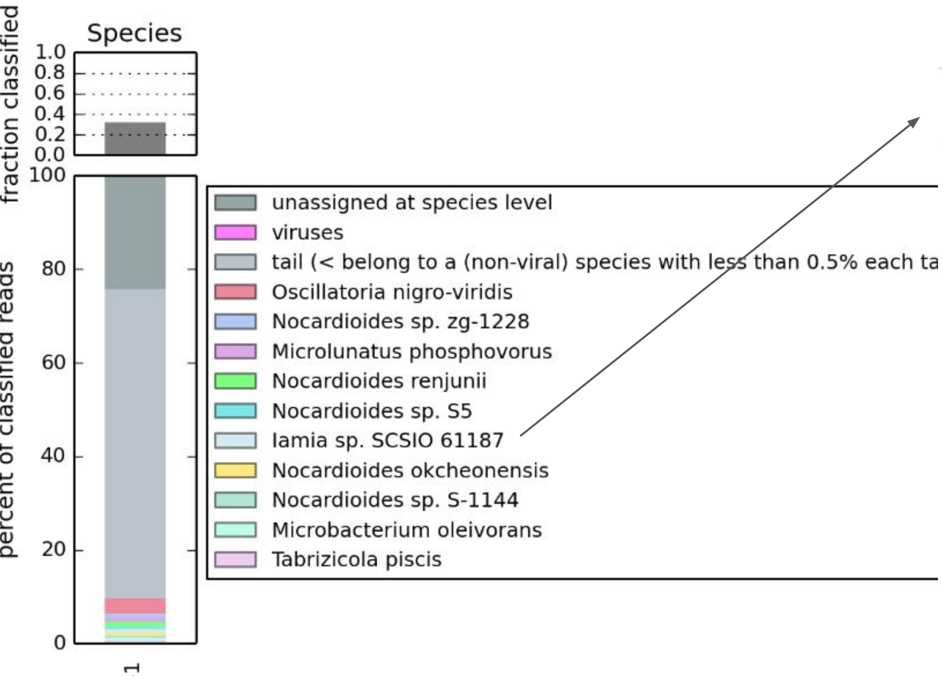


**~67% of completeness and ~14% of contamination**

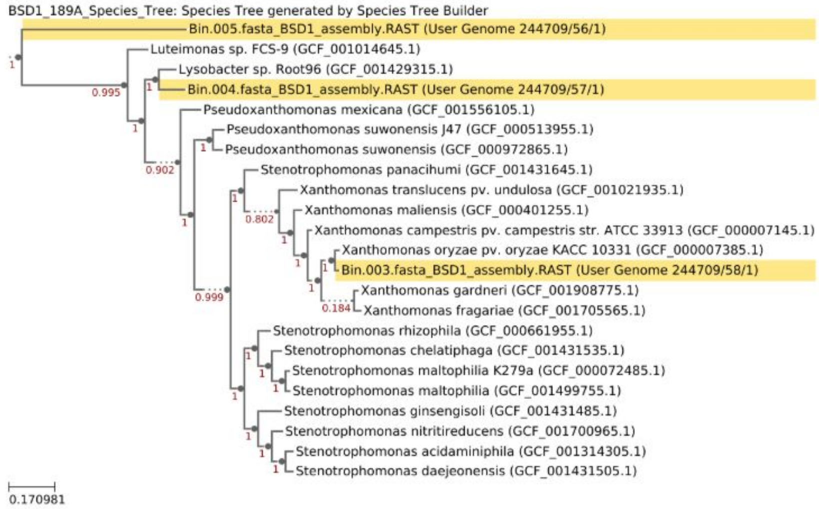

# Functional profiling: DRAM (Genomes)

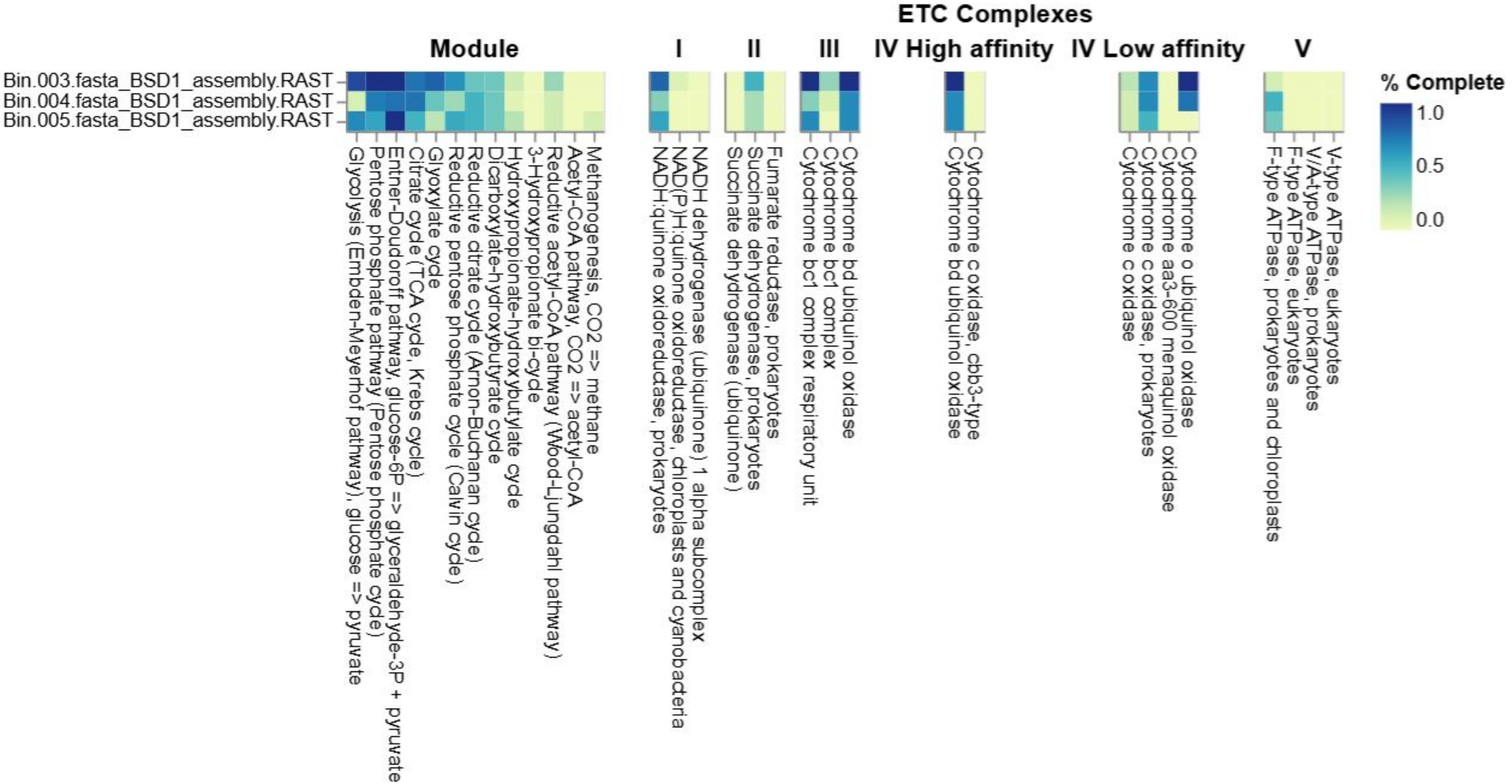

# Functional profiling: DRAM (Genomes)

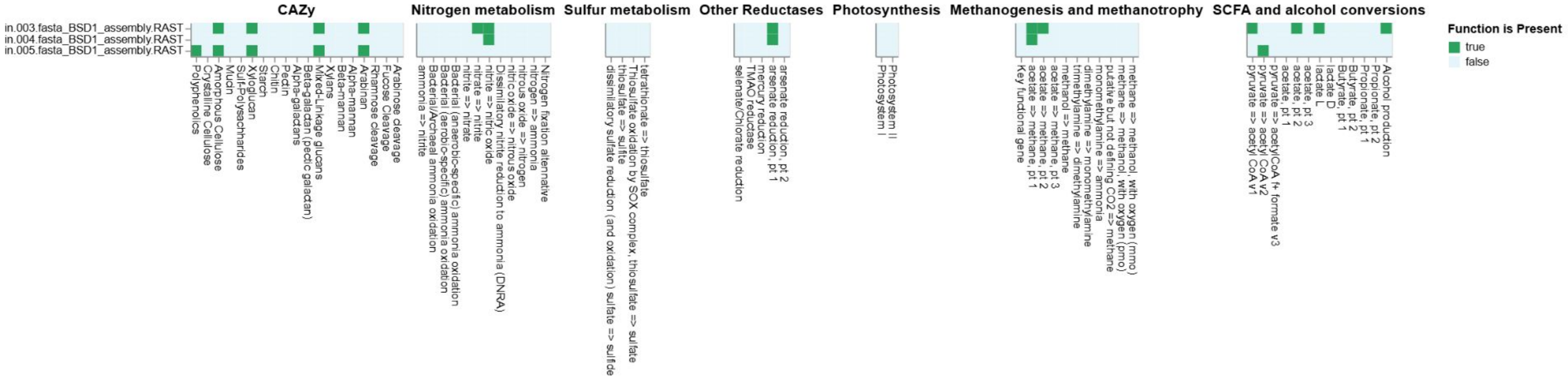

# Functional profiling: Bioelement

arsC and arsC2 are genes for microbial survival in arsenic-contaminated environments, frequently acting through different mechanisms to reduce arsenic toxicity

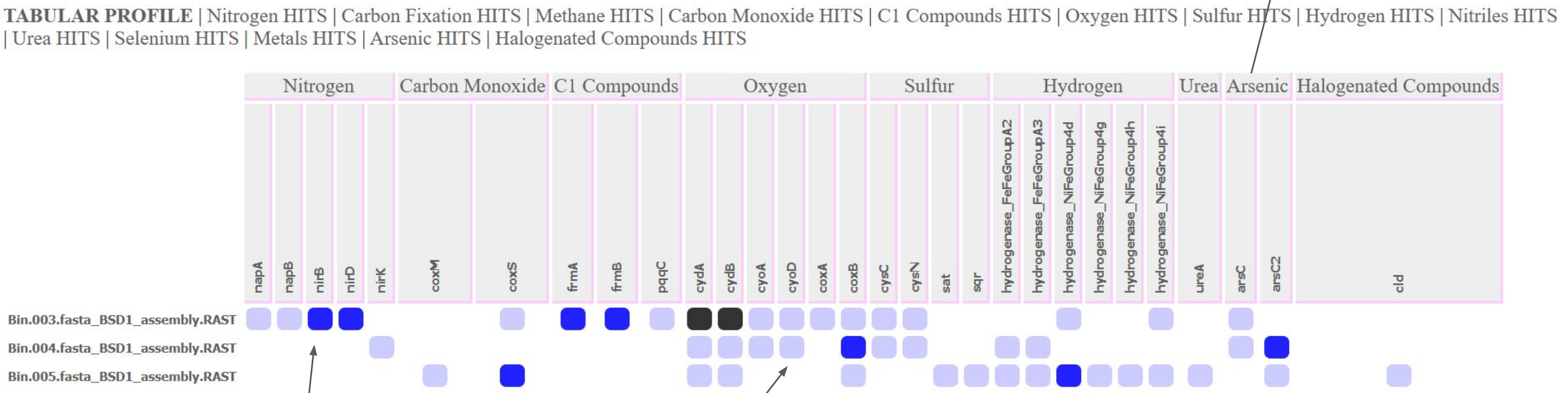


denitrifying organism

oxygen cycle

potential anaerobic hydrogen metabolism

# Functional profiling: PhyloMarker

The absence of archaeal markers confirms that all three bins correspond to bacteria.

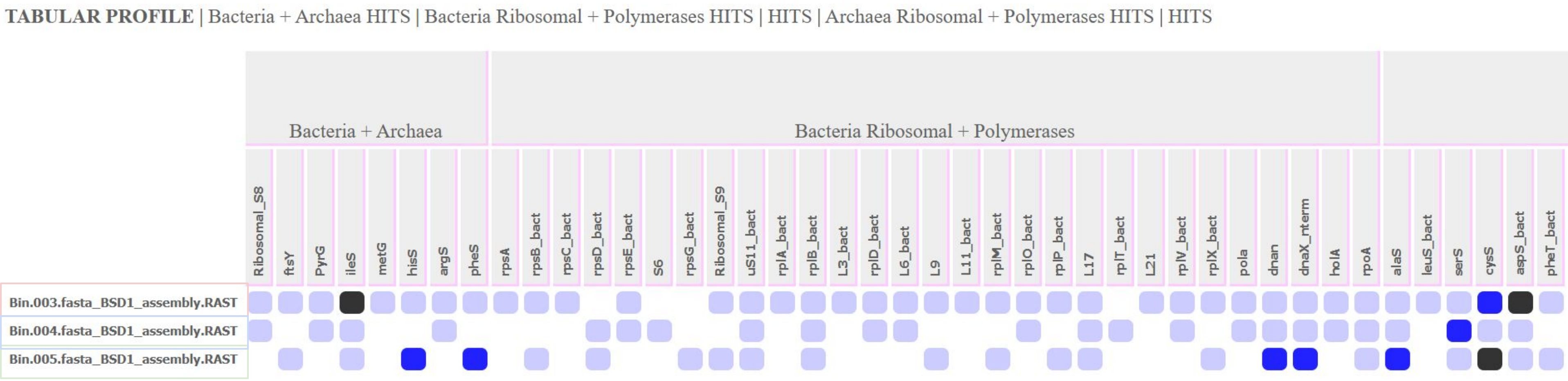

Grazie!!

Gracias!!

Thanks!!

# Functional profiling: FAMA

Run info | BSD1_189A_S182_prinseq Functional profile | BSD1_189A_S182_prinseq Functional groups | BSD1_189A_S182_prinseq Taxonomy profile

| Function category | | efpkg | Raw sequence count | Amino acid identity %, average |
|---|---|---|---|---|
| Ammonification | NrfA, NrfH | 1.25683 | 1715 | 77.38 |
| Ammonium oxidation | AmoA/B/C, HAO | 0.38252 | 374 | 87.99 |
| Anaerobic ammonium oxidation | HAO | 0.03232 | 65 | 86.86 |
| Denitrification | NirK, NirS, cNorB/qNor, NosZ | 3.08900 | 6141 | 79.04 |
| Nitrate assimilatory reduction | NasA, NasB | 4.63306 | 12333 | 79.48 |
| Nitrate dissimilatory reduction | NarG/NxrA, NarH/NxrB, NarI, NarJ, NapA–H | 5.75409 | 10996 | 86.89 |
| Nitrite assimilation | NirA, NirB, NirD, NirK, NirS, NirU | 6.56392 | 13092 | 82.15 |
| Nitrogen fixation | NifB, NifD, NifH, NifK | 0.17028 | 269 | 90.67 |
| Urease | UreA, UreB, UreC | 6.97285 | 8350 | 88.26 |

| Nitrogen Cycle — Key Individual Genes | | | | |
|---|---|---|---|---|
| **NarG_NxrA** | 1.18 | 140,479 | 88.85% | Nitrate reductase α / Nitrite oxidoreductase α |
| **NarH_NxrB** | 1.09 | 160,209 | 89.60% | Nitrate reductase β / Nitrite oxidoreductase β |
| **NasA** | 3.78 | 583,107 | 79.85% | Assimilatory nitrate reductase large subunit |
| **NirB** | 3.06 | 361,878 | 84.25% | Nitrite reductase (NADH) large subunit |
| **NirD** | 2.24 | 400,174 | 78.42% | Nitrite reductase (NADH) small subunit |
| **UreC** | 2.79 | 56,222 | 89.66% | Urease alpha subunit |

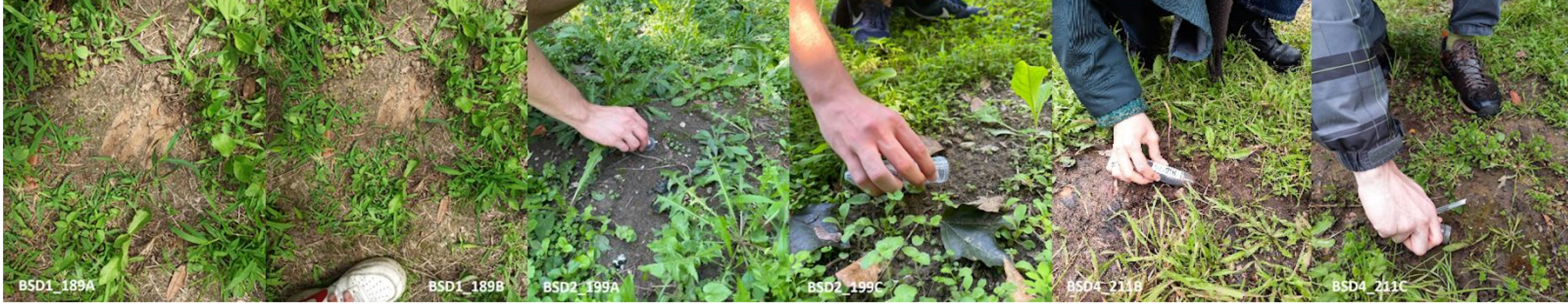


# BSD2_199A_S184

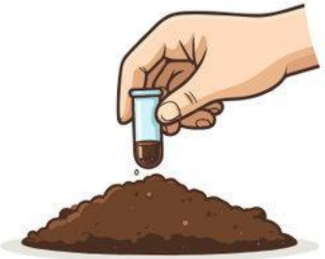

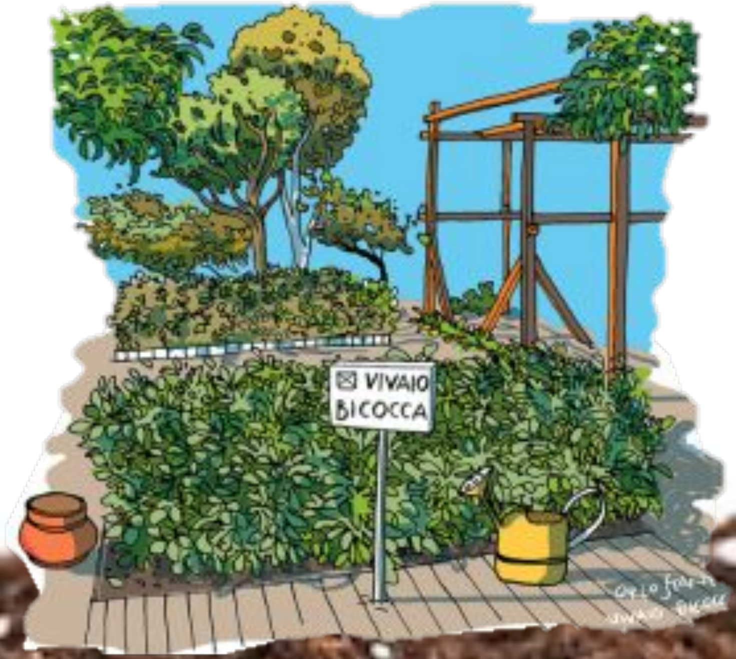


Margherita Emma
Gorla
Sharon Cristal Rubrica

Sampling Area: Vivaio Bicocca
Sample Type: Soil
Sampling Date: 29/09/23

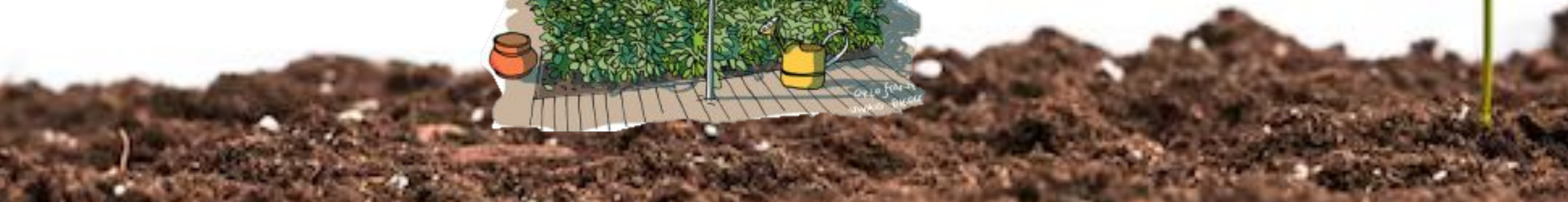

FastQC

## Per base sequence quality

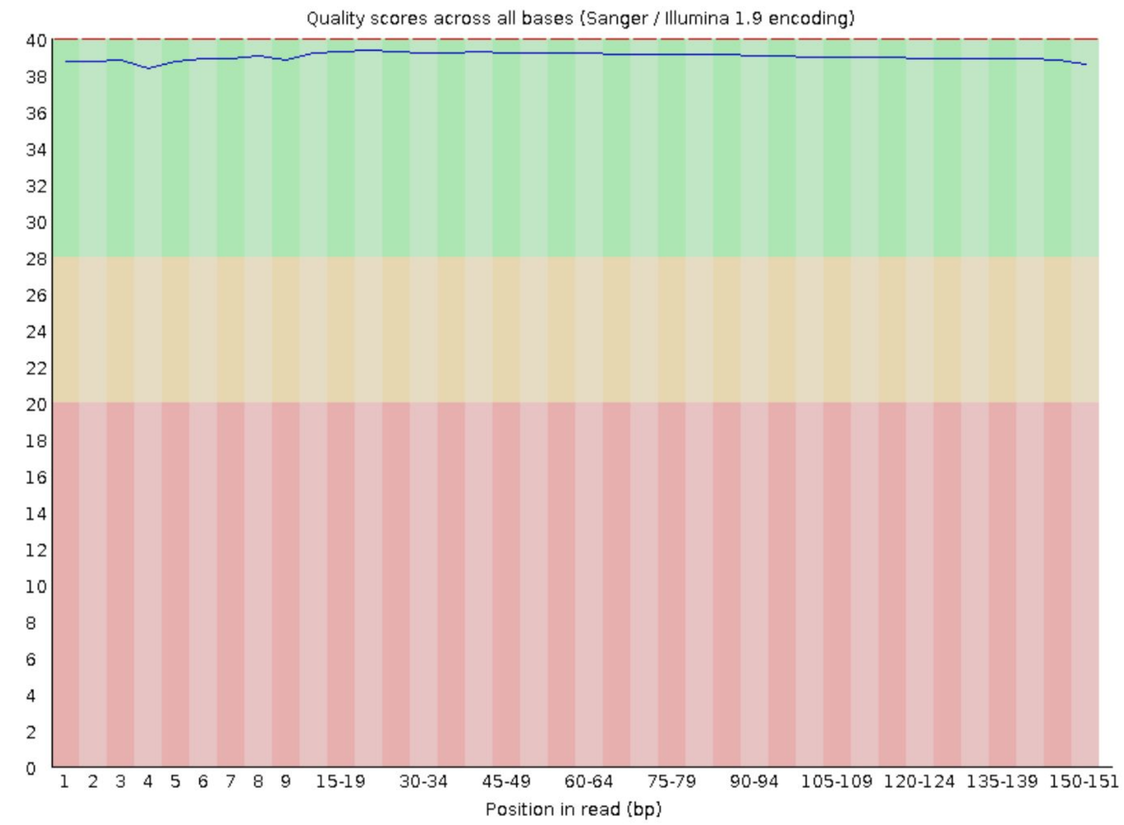


## Per base sequence quality

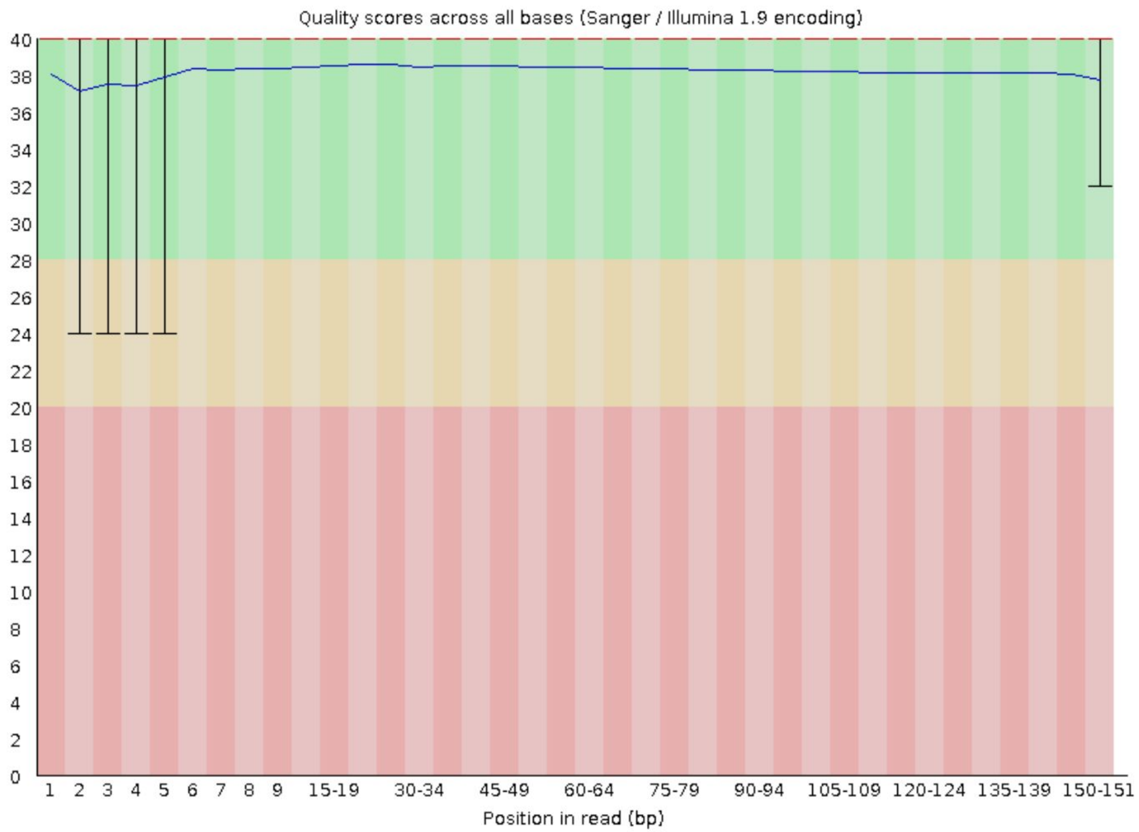

## Adapter Content

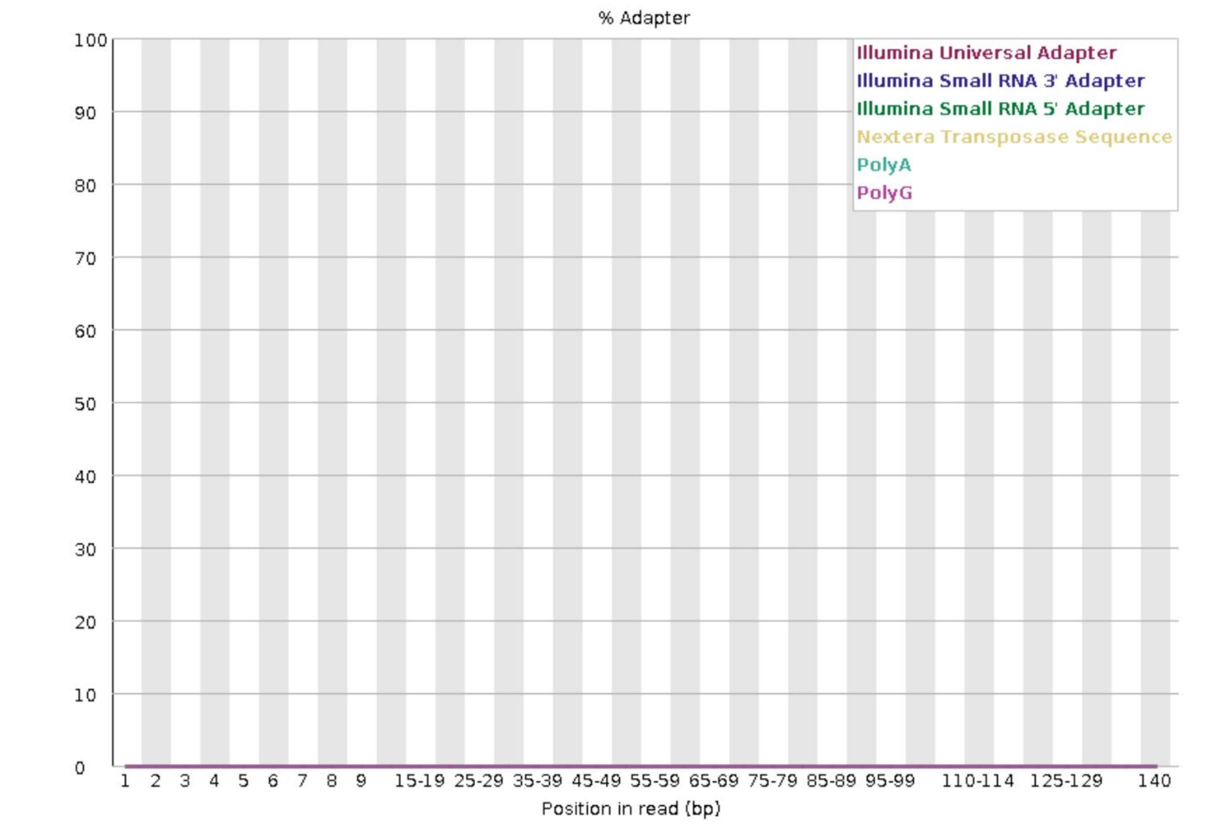


## Adapter Content

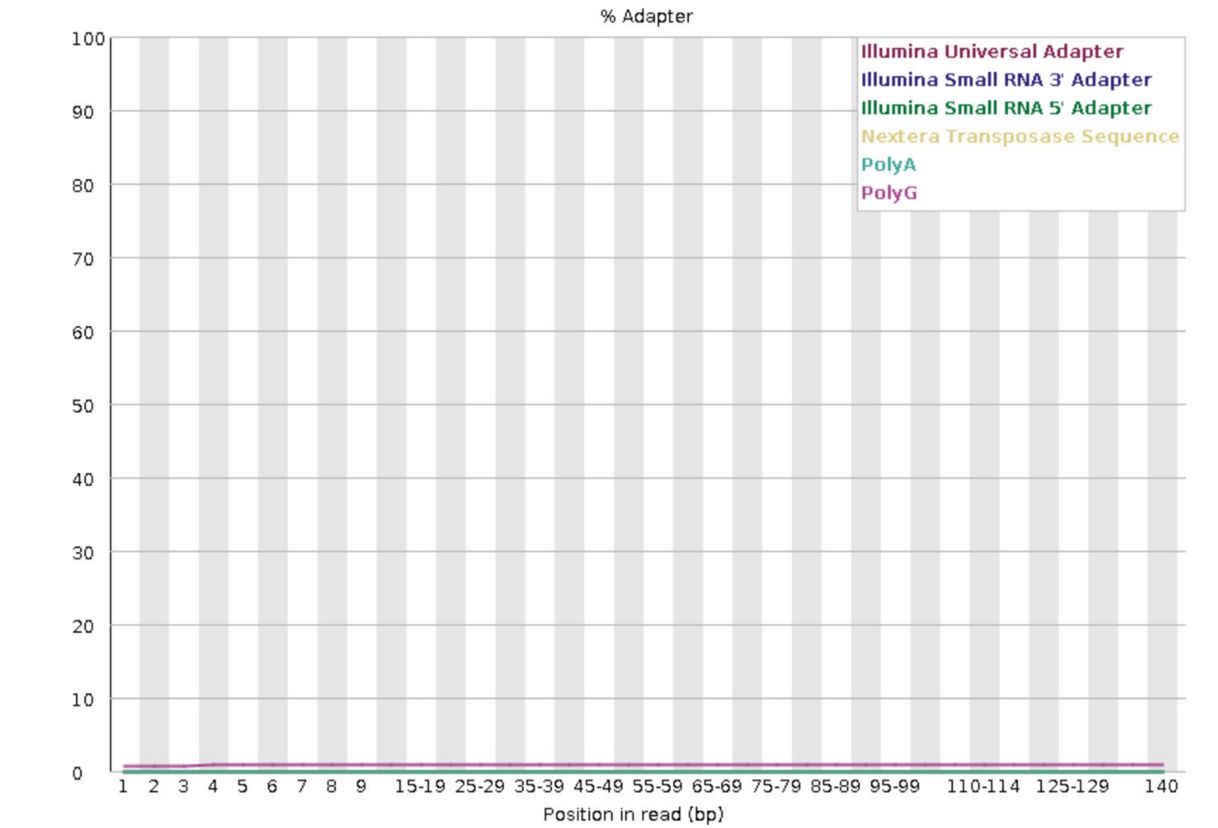


# Overrepresented sequences

| Sequence | Count | Percentage | Possible Source |
|---|---|---|---|
| GGGGGGGGGGGGGGGGGGGGGGGGGGGGGGGGGGGGGGGGGGGGGGGGGG | 278533 | 0.8235301248439063 | No Hit |

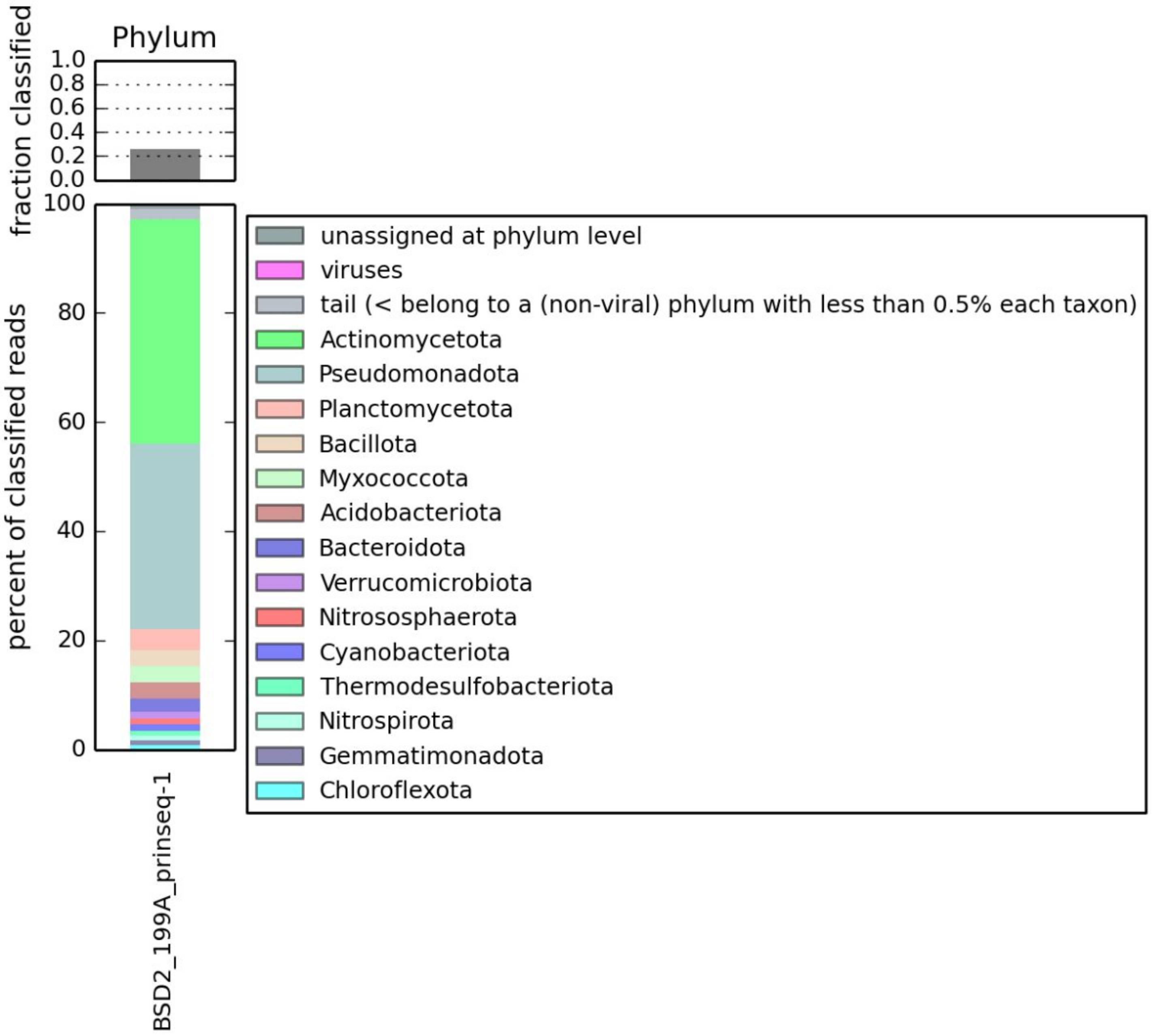

Phylum
fraction classified
1.0
0.8
0.6
0.4
0.2
0.0
percent of classified reads
100
80
60
40
20
0
BSD2_199A_prinseq-1
unassigned at phylum level
viruses
tail (< belong to a (non-viral) phylum with less than 0.5% each taxon)
Actinomycetota
Pseudomonadota
Planctomycetota
Bacillota
Myxococcota
Acidobacteriota
Bacteroidota
Verrucomicrobiota
Nitrososphaerota
Cyanobacteriota
Thermodesulfobacteriota
Nitrospirota
Gemmatimonadota
Chloroflexota

# QUAST

Quality Assessment Tool for Genome Assemblies by CAB

16 February 2026, Monday, 08:42:28

View in Icarus contig browser

All statistics are based on contigs of size >= 500 bp, unless otherwise noted (e.g., "# contigs (>= 0 bp)" and "Total length (>= 0 bp)" include all contigs).

Worst Median Best

Show heatmap

| Statistics without reference | BSD2_199A_MEGAHIT_sensitive | BSD2_199A_MEGAHIT_large | BSD2_199A_prinseq_metaSPAdes | BSD2_199A_IDBA |
|---|---|---|---|---|
| # contigs | 4440 | 4493 | 4082 | 1266 |
| # contigs (>= 0 bp) | 4440 | 4493 | 4082 | 1266 |
| # contigs (>= 1000 bp) | 4440 | 4493 | 4082 | 1266 |
| # contigs (>= 10000 bp) | 15 | 25 | 16 | 4 |
| # contigs (>= 100000 bp) | 0 | 0 | 0 | 0 |
| # contigs (>= 1000000 bp) | 0 | 0 | 0 | 0 |
| Largest contig | 22 599 | 26 425 | 23 854 | 15 741 |
| Total length | 12 817 828 | 13 251 239 | 11 944 477 | 3 948 091 |
| Total length (>= 0 bp) | 12 817 828 | 13 251 239 | 11 944 477 | 3 948 091 |
| Total length (>= 1000 bp) | 12 817 828 | 13 251 239 | 11 944 477 | 3 948 091 |
| Total length (>= 10000 bp) | 195 115 | 342 328 | 208 048 | 49 469 |
| Total length (>= 100000 bp) | 0 | 0 | 0 | 0 |
| Total length (>= 1000000 bp) | 0 | 0 | 0 | 0 |
| N50 | 2726 | 2772 | 2787 | 2989 |
| N75 | 2276 | 2309 | 2303 | 2432 |
| L50 | 1639 | 1629 | 1498 | 457 |
| L75 | 2932 | 2945 | 2684 | 827 |
| GC (%) | 61.21 | 62.29 | 61.77 | 56.62 |
| **Mismatches** | | | | |
| # N's | 0 | 0 | 4710 | 0 |
| # N's per 100 kbp | 0 | 0 | 39.43 | 0 |

# Binning

MaxBin2

JGI JOINT GENOME INSTITUTE

CONCOCT

Binned contigs: 2749

Input contigs: 4493

Number of bins: 3

DAS-Tool

| Bin Name | Marker Lineage | # Genomes | # Markers | # Marker Sets | 0 | 1 | 2 | 3 | 4 | 5+ | Completeness | Contamination |
|---|---|---|---|---|---|---|---|---|---|---|---|---|
| bin.001 | o__Rhizobiales | 107 | 485 | 316 | 401 | 80 | 4 | 0 | 0 | 0 | 15.23 | 0.4 |
| bin.002 | k__Bacteria | 5449 | 104 | 58 | 64 | 34 | 6 | 0 | 0 | 0 | 41.94 | 5.16 |
| bin.003 | k__Archaea | 207 | 145 | 103 | 42 | 73 | 26 | 4 | 0 | 0 | 68.77 | 19.69 |

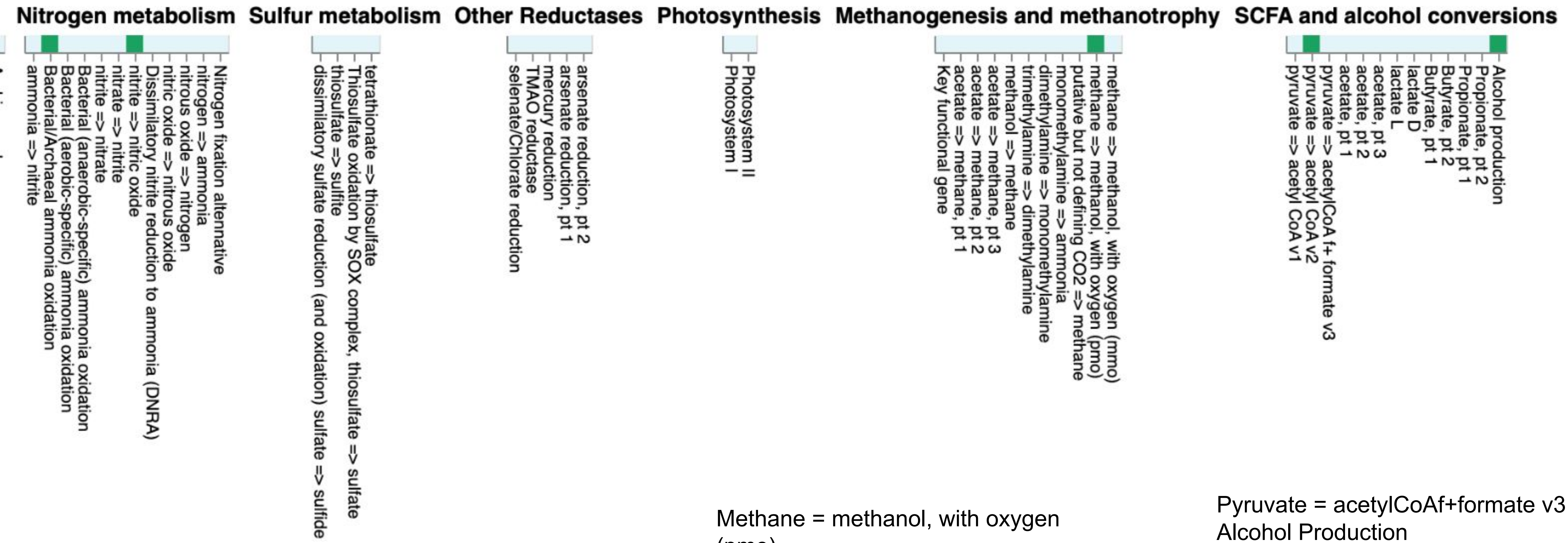


Methane = methanol, with oxygen (pmo)

Pyruvate = acetylCoAf+formate v3
Alcohol Production

Nitrite = nitric oxide
Bacterial/Archaeal amonnia oxidation

GTDB-TK

| | |
|---|---|
| bin.001.fa | d__Bacteria; p__Pseudomonadota; c__Alphaproteobacteria; o__Rhizobiales; f__Xanthobacteraceae; g__VAZQ01; s__ |
| bin.002.fa | d__Bacteria; p__Actinomycetota; c__Actinomycetia; o__Mycobacteriales; f__Pseudonocardiaceae; g__; s__ |
| bin.003.fa | d__Archaea; p__Thermoproteota; c__Nitrososphaeria; o__Nitrososphaerales; f__Nitrososphaeraceae; g__TH5896; s__ |

GCA_014523625.1

Nitrososphaerales archaeon

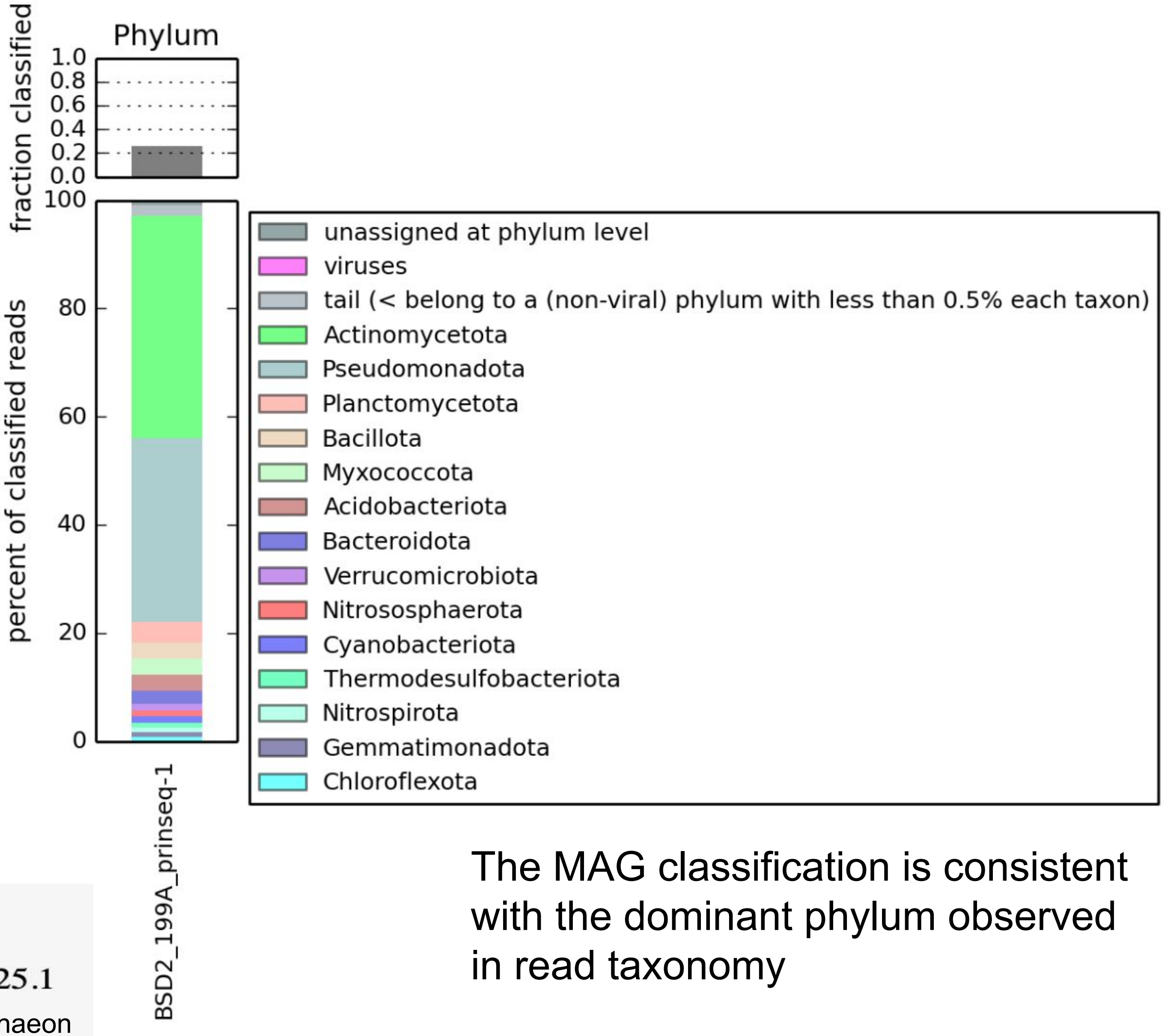


The MAG classification is consistent with the dominant phylum observed in read taxonomy

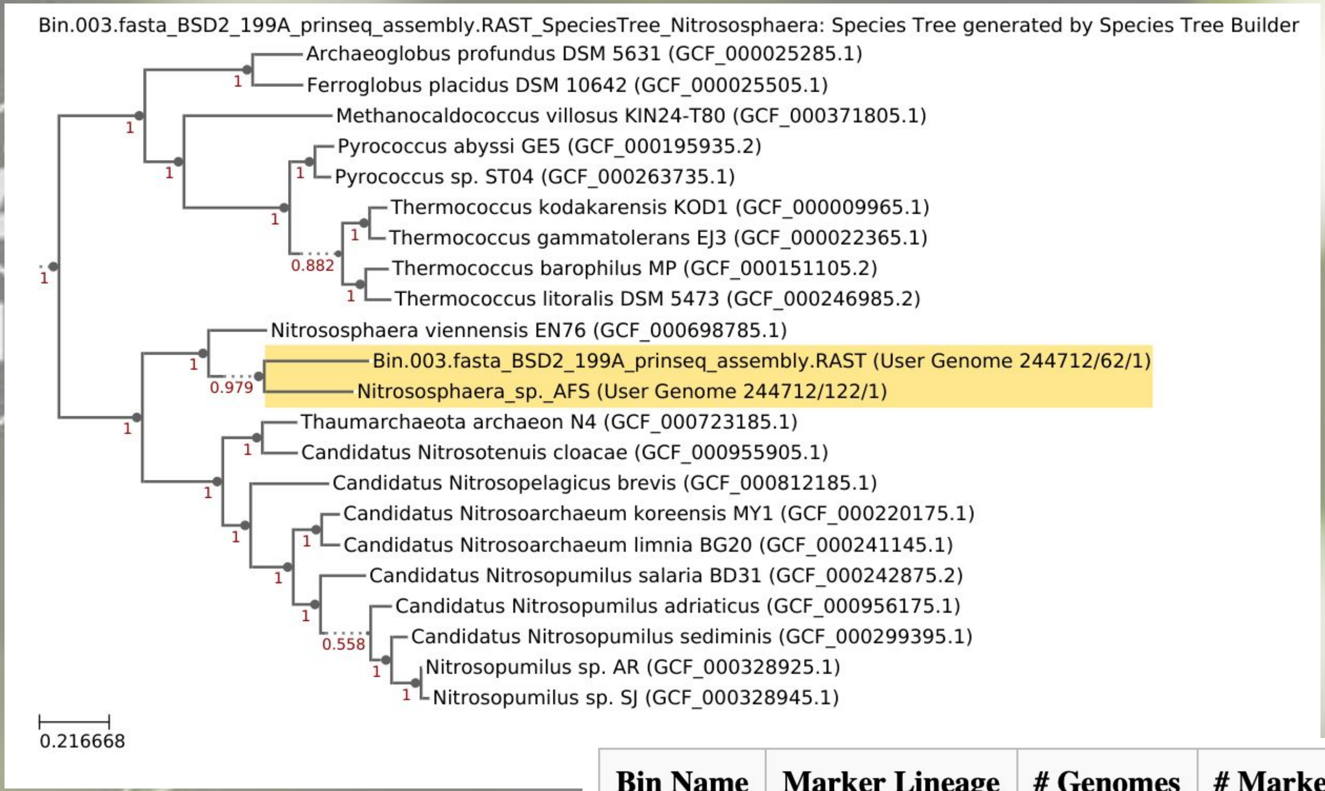


| Bin Name | Marker Lineage | # Genomes | # Markers | # Marker Sets | 0 | 1 | 2 | 3 | 4 | 5+ | Completeness | Contamination |
|---|---|---|---|---|---|---|---|---|---|---|---|---|
| bin.001 | o__Rhizobiales | 107 | 485 | 316 | 401 | 80 | 4 | 0 | 0 | 0 | 15.23 | 0.4 |
| bin.002 | k__Bacteria | 5449 | 104 | 58 | 64 | 34 | 6 | 0 | 0 | 0 | 41.94 | 5.16 |
| bin.003 | k__Archaea | 207 | 145 | 103 | 42 | 73 | 26 | 4 | 0 | 0 | 68.77 | 19.69 |

| Bin Name | Marker Lineage | # Genomes | # Markers | # Marker Sets | 0 | 1 | 2 | 3 | 4 | 5+ | Completeness | Contamination |
|---|---|---|---|---|---|---|---|---|---|---|---|---|
| Nitrososphaera_sp._AFS | k__Archaea | 207 | 145 | 103 | 7 | 135 | 3 | 0 | 0 | 0 | 93.2 | 2.91 |

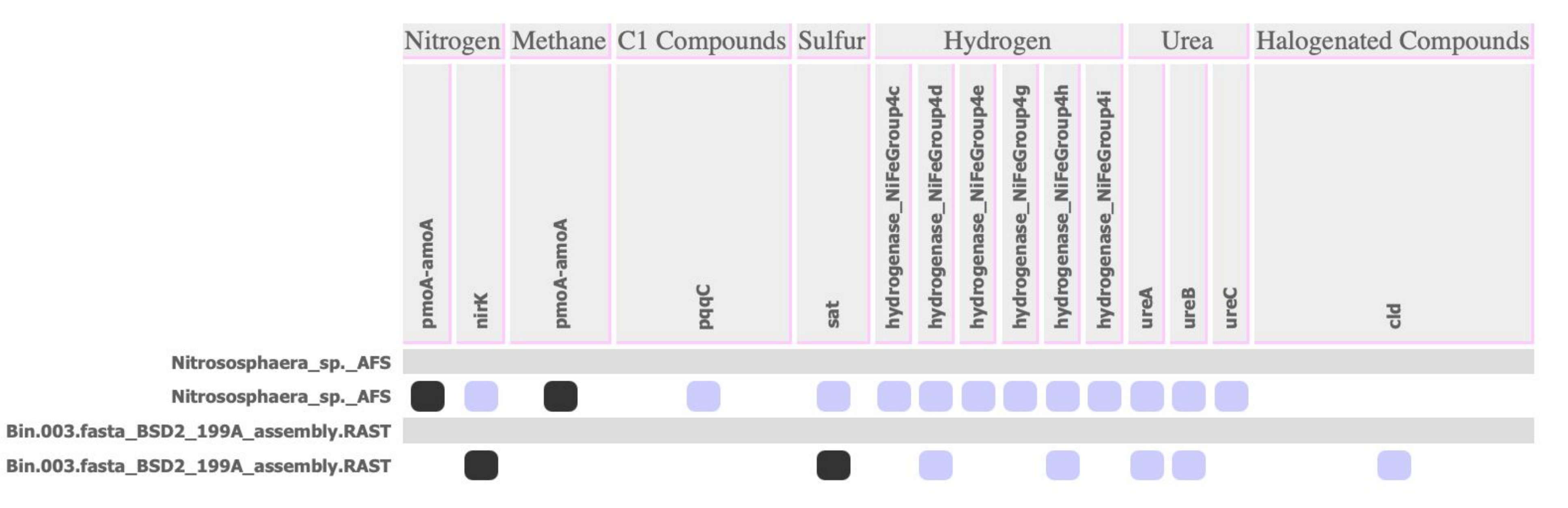


<u>Nitrogen Metabolism</u>

- **pmoA-amoA family** – a key step for ammonia oxidation
- **nirK** – nitrite reductase, in ammonia oxidizing archaea, often linked to nitrifier denitrification

<u>Methane Oxidation</u>: Likely reflects ammonia oxidation because it is homologous to pmoA.

<u>Urea</u>: Presence of ure genes indicated ammonia oxidizing archaea

<u>Sulfur</u>: Involved in sulfate activation

<u>Halogenated Compounds</u>: Suggests detoxification capability

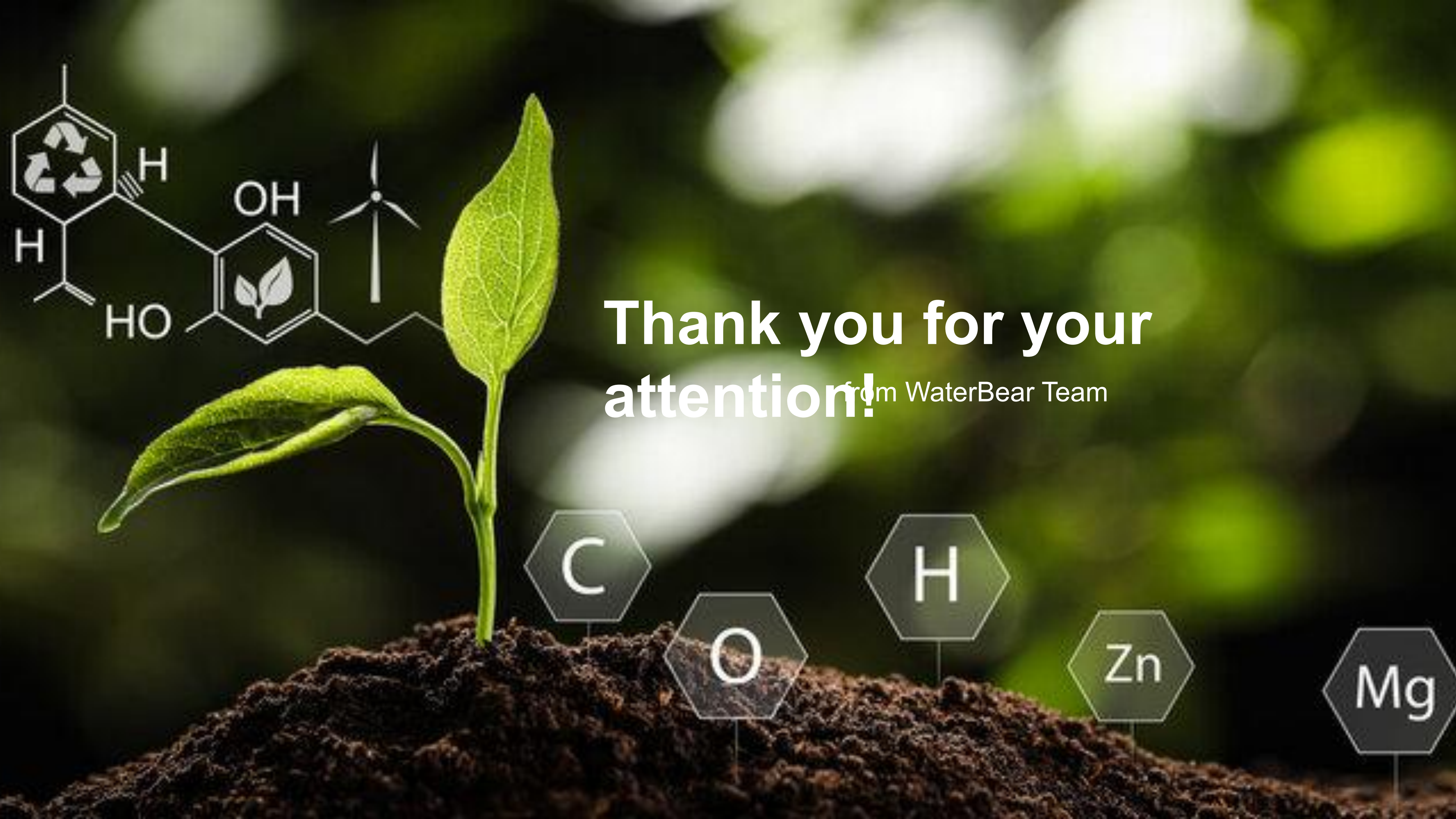


# Thank you for your attention!

from WaterBear Team
