# Supplementary material for: Fantastic microbes and where to find them: evaluating learning-by-doing outcomes in a crowdfunded metagenomics workshop
Source: FEMS Microbiol Lett. 2026 Aug 17;373:fnag093. doi: 10.1093/femsle/fnag093 (PMC13501537; doi:10.1093/femsle/fnag093)
Supplement: Supplementary file 1 [file fnag093_supplemental_files.zip › 2_SupplMat.pdf]

# From Reads to Function Workshop - Hybrid Workshop on Metagenomics and the Fantastic Microbes Around Us

The objective of this questionnaire, which is completely **anonymous**, is to evaluate the educational impact of the Workshop,

improve future training activities, and analyze learning outcomes for potential scientific publication.
The questionnaire does not collect any personal or identifiable information (such as names, email addresses, IP addresses, or institutional identifiers). All responses will be analyzed and reported in aggregated and fully anonymous form. It will not be possible to identify individual participants.

By proceeding with the questionnaire, you indicate your informed consent to participate and to allow the anonymous use of the collected data for the purposes described above.
Estimated time to complete: 5-10 minutes.

This study was conducted in accordance with applicable European data protection regulations (EU Regulation 2016/679) - GDPR.

For any inquiries, you can contact us at: biome.research.team@gmail.com

Thank you for your cooperation!

* Indicates required question

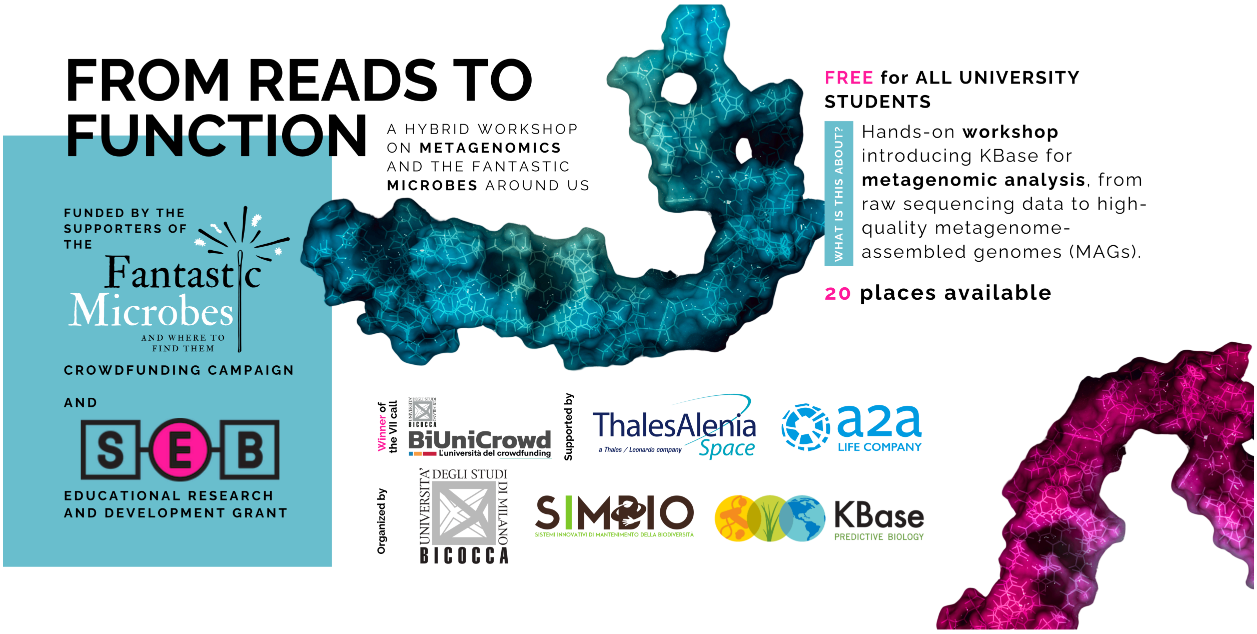

1. I have read the information above and agree to participate. *

*Mark only one oval.*

- Yes
- No

Demographic questions

2. What is your current academic status? *

*Mark only one oval.*

- Undergraduate student (Bachelor's level)
- Graduate student (Master’s level)
- Graduate (non-PhD or PhD candidate)
- PhD level (already completed the PhD)

3. What is your primary field of study or background? *

*Mark only one oval.*

- Biology
- Biotechnology
- Bioinformatics
- Computer Science
- Environmental Science
- Other:

4. How would you describe your previous experience with bioinformatics? *

*Mark only one oval.*

- ( ) None
- ( ) Basic (e.g., attended courses)
- ( ) Intermediate (performed analyses independently)
- ( ) Advanced (regularly perform analyses)

5. Before this workshop, how familiar were you with metagenomic analysis? *

*Mark only one oval.*

| | 1 | 2 | 3 | 4 | 5 | |
|---|---|---|---|---|---|---|
| Not | ( ) | ( ) | ( ) | ( ) | ( ) | Extremely familiar |

6. Before this workshop, had you used KBase? *

*Mark only one oval.*

- ( ) No
- ( ) Yes, once or twice
- ( ) Yes, occasionally
- ( ) Yes, regularly

## Skills of Science Inquiry

7. "I **currently** have the skills necessary to…" *

Please indicate how much you **DISAGREE** or **AGREE** with each of the following statements by selecting the cell in the appropriate column. Please respond as you really feel, rather than how you think "most people" feel.

*Mark only one oval per row.*

| | Strongly Disagree | Disagree | Neutral | Agree | Strongly Agree |
|---|---|---|---|---|---|
| **Inspect and document basic properties of metagenomic datasets within KBase** | ⬭ | ⬭ | ⬭ | ⬭ | ⬭ |
| **Accurately identify taxonomic and/or functional features from metagenomic data with KBase** | ⬭ | ⬭ | ⬭ | ⬭ | ⬭ |
| **Understand the KBase data analysis framework** | ⬭ | ⬭ | ⬭ | ⬭ | ⬭ |
| **Successfully upload and import metagenomic datasets within the KBase platform** | ⬭ | ⬭ | ⬭ | ⬭ | ⬭ |
| **Process metagenomic data in a standardized manner with KBase** | ⬭ | ⬭ | ⬭ | ⬭ | ⬭ |
| **Use the KBase tools to answer a** | ⬭ | ⬭ | ⬭ | ⬭ | ⬭ |

| | | | | | |
|---|---|---|---|---|---|
| biological question | | | | | |
| Interpret the meaning of metagenomic data presented in KBase maps, charts, graphs, etc | | | | | |
| Conduct analyses using the Kbase Workshop data | | | | | |
| Use the Kbase Workshop data as a source of evidence | | | | | |
| Design a study related to the KBase Workshop data | | | | | |
| Communicate the KBase Workshop findings to others | | | | | |
| Train others to participate in KBase Workshops | | | | | |

8. "**Prior to participating** in in the Workshop I had the skills necessary to..." *

Please indicate how much you **DISAGREE** or **AGREE** with each of the following statements by selecting the cell in the appropriate column. Please respond as you really feel, rather than how you think "most people" feel.

*Mark only one oval per row.*

| | Strongly Disagree | Disagree | Neutral | Agree | Strongly Agree |
|---|---|---|---|---|---|
| **Inspect and document basic properties of metagenomic datasets within KBase** | | | | | |
| **Accurately identify taxonomic and/or functional features from metagenomic data with KBase** | | | | | |
| **Understand the KBase data analysis framework** | | | | | |
| **Successfully upload and import metagenomic datasets within the KBase platform** | | | | | |
| **Process metagenomic data in a standardized manner with KBase** | | | | | |
| **Use the KBase tools to answer a** | | | | | |

| | | | | | |
|---|---|---|---|---|---|
| biological question | | | | | |
| Interpret the meaning of metagenomic data presented in KBase maps, charts, graphs, etc | | | | | |
| Conduct analyses using the Kbase Workshop data | | | | | |
| Use the Kbase Workshop data as a source of evidence | | | | | |
| Design a study related to the KBase Workshop data | | | | | |
| Communicate the KBase Workshop findings to others | | | | | |
| Train others to participate in KBase Workshops | | | | | |

Self-Efficacy for Learning and Doing Science

9. These statements are about how you **currently** feel about learning and understanding science topics *

Please indicate how much you **DISAGREE** or **AGREE** with each of the following statements by selecting the cell in the appropriate column. Please respond as you really feel, rather than how you think “most people” feel.

**Currently**...

*Mark only one oval per row.*

| | Strongly Disagree | Disagree | Neutral | Agree | Strongly Agree |
|---|---|---|---|---|---|
| **I think I am pretty good at understanding topics related to metagenomic data analysis.** | ⬭ | ⬭ | ⬭ | ⬭ | ⬭ |
| **Compared to other people my age, I think I can quickly understand new topics related to metagenomic analysis.** | ⬭ | ⬭ | ⬭ | ⬭ | ⬭ |
| **It takes me a long time to understand new topics about metagenomic data analysis.** | ⬭ | ⬭ | ⬭ | ⬭ | ⬭ |
| **I feel confident in my ability to explain metagenomic analysis related topics to others.** | ⬭ | ⬭ | ⬭ | ⬭ | ⬭ |

10. These statements are about how you **currently** feel about doing scientific activities. *

Please indicate how much you **DISAGREE** or **AGREE** with each of the following statements by selecting the cell in the appropriate column. Please respond as you really feel, rather than how you think "most people" feel.

**Currently**...

*Mark only one oval per row.*

| | Strongly Disagree | Disagree | Neutral | Agree | Strongly Agree |
|---|---|---|---|---|---|
| **I think I'm pretty good at following instructions for metagenomic data analysis using KBase.** | ⬭ | ⬭ | ⬭ | ⬭ | ⬭ |
| **Compared to other people my age, I think I can perform metagenomic analysis using KBase pretty well** | ⬭ | ⬭ | ⬭ | ⬭ | ⬭ |
| **It takes me a long time to understand how to perform metagenomic analysis using KBase.** | ⬭ | ⬭ | ⬭ | ⬭ | ⬭ |
| **I feel confident about my ability to explain how to perform metagenomic analysis using KBase to others.** | ⬭ | ⬭ | ⬭ | ⬭ | ⬭ |

11. These statements are about how you felt about learning and understanding science topics **prior to participating** in the workshop. *

Please indicate how much you **DISAGREE** or **AGREE** with each of the following statements by selecting the cell in the appropriate column. Please respond as you really feel, rather than how you think "most people" feel.

**Prior to participating** in the workshop...

*Mark only one oval per row.*

| | Strongly Disagree | Disagree | Neutral | Agree | Strongly Agree |
|---|---|---|---|---|---|
| **I think I was pretty good at understanding topics related to metagenomic data analysis with KBase.** | ◯ | ◯ | ◯ | ◯ | ◯ |
| **Compared to other people my age, I think I was able to quickly understand new topics related to metagenomic analysis.** | ◯ | ◯ | ◯ | ◯ | ◯ |
| **It took me a long time to understand new topics about metagenomic data analysis.** | ◯ | ◯ | ◯ | ◯ | ◯ |
| **I felt confident in my ability to explain metagenomic analysis related topics to others.** | ◯ | ◯ | ◯ | ◯ | ◯ |

12. These statements are about how you felt about doing scientific activities **prior to participating** in the workshop *

Please indicate how much you **DISAGREE** or **AGREE** with each of the following statements by selecting the cell in the appropriate column. Please respond as you really feel, rather than how you think “most people” feel.

**Prior to participating** in the workshop...

*Mark only one oval per row.*

| | Strongly Disagree | Disagree | Neutral | Agree | Strongly Agree |
|---|---|---|---|---|---|
| **I think I was pretty good at following instructions for metagenomic data analysis using KBase.** | ⬭ | ⬭ | ⬭ | ⬭ | ⬭ |
| **Compared to other people my age, I think I was able to perform metagenomic analysis using KBase pretty well** | ⬭ | ⬭ | ⬭ | ⬭ | ⬭ |
| **It took me a long time to understand how to perform metagenomic analysis using KBase.** | ⬭ | ⬭ | ⬭ | ⬭ | ⬭ |
| I felt confident about my ability to explain how to perform metagenomic analysis using KBase to others. | | | | | |

| | | | | | |
|---|---|---|---|---|---|
| I felt confident about my ability to explain how to perform metagenomic analysis using KBase to others. | ⬭ | ⬭ | ⬭ | ⬭ | ⬭ |

Understanding of the metagenomic analysis process

13. When you hear or read the term *metagenomic data analysis using KBase*, you have: *

*Mark only one oval.*

- ⬭ Little understanding of what it means
- ⬭ A general sense of what it means
- ⬭ A clear understanding of what it means

14. Please describe in your own words what "*metagenomic data analysis using KBase*" means and what steps it involves. *

_______________________________________________

_______________________________________________

_______________________________________________

_______________________________________________

_______________________________________________

Additional Comments (optional)

15. Please use the space below to share any feedback, suggestions, or general impressions about the workshop and your experience with metagenomic analysis using KBase.

This content is neither created nor endorsed by Google.

Google Forms
